# Boron-Containing Analogs of Fosmidomycin: Benzoxaborole Derivatives Exhibit Promising Activity Against Resistant Pathogens

**DOI:** 10.1021/acsomega.5c02701

**Published:** 2025-07-19

**Authors:** James M. Gamrat, Christopher L. Orme, Giulia Mancini, Sarah J. Burke, Latifah M. Alhthlol, Rebecca C. Colandrea, Bryan C. Figula, Dylan T. Tomares, Jason E. Heindl, John W. Tomsho

**Affiliations:** † 6557 Saint Joseph’s University, University City Campus, Department of Chemistry & Biochemistry, 600 S. 43rd St, Philadelphia, Pennsylvania 19104, United States; ‡ Rowan University, Department of Biological & Biomedical Sciences, 201 Mullica Hill Rd, Glassboro, New Jersey 08028, United States

## Abstract

The rise of antimicrobial resistance presents an urgent challenge that necessitates the development of novel therapeutic agents with distinct mechanisms of action. This research explores boron-containing compounds as potential neutral phosphate/phosphonate isosteres of fosmidomycin, a potent inhibitor of 1-deoxy-d-xylulose-5-phosphate reductoisomerase (IspC) within the nonmevalonate isoprenoid biosynthesis (MEP) pathway, with limited clinical utility due to poor pharmacokinetics. We report the synthesis of a library of 15 boron-containing analogs of fosmidomycin and their comprehensive evaluation as IspC inhibitors and antimicrobial agents. The compounds did not demonstrate significant activity against the intended IspC target, thus providing evidence that these boron moieties may have limited utility as phosphonate isosteres in this system. However, our investigation yielded unexpected and valuable antimicrobial discoveries. Several benzoxaborole compounds demonstrated significant activity against pathogenic microbes, including methicillin-resistant *Staphylococcus aureus* (MRSA), *E. coli*, and *C. albicans*. Mechanistic studies confirmed that these compounds operate through alternative pathways distinct from MEP pathway inhibition. These results provide a foundation for the rational design of next-generation boron-containing antimicrobials with enhanced potency and selectivity against resistant pathogens, including MRSA.

## Graphical abstract

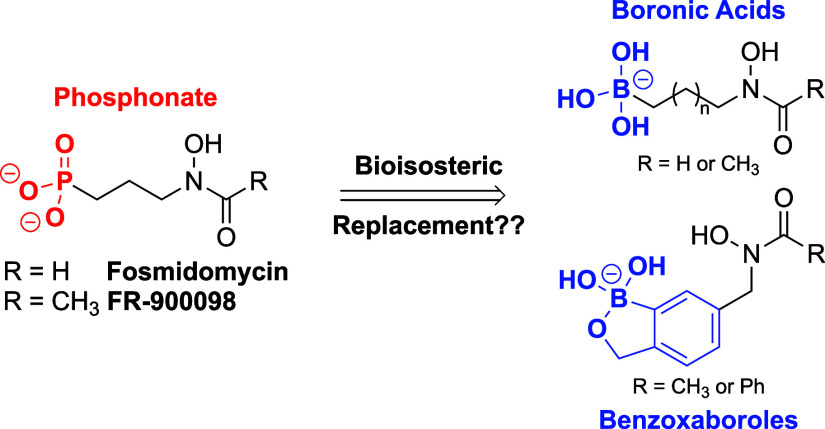

## Introduction

Infectious diseases have continued to be a major public health concern and are estimated to cause a quarter of the annual deaths worldwide.^1^ Although many infections are common and successfully treated, more serious infections caused by microorganisms can result in severe illness and death. Two particularly deadly infections are tuberculosis (TB), caused by the bacterium *Mycobacterium tuberculosis* (Mtb), and malaria, caused by the parasite *Plasmodium falciparum* (*P. falciparum*). These pathogens continue to plague developing countries and result in over a million deaths annually.^2,3^ Also, the emergence of antimicrobial resistance, such as in methicillin-resistant *Staphylococcus aureus* (MRSA), has complicated the treatment of some pathogens which then require more intensive treatment.^4^ Though there are currently drugs available that are effective against infections caused by resistant parasites and bacteria, it is essential to continually develop new antimicrobial agents to decrease the burden of these diseases and to adapt to the evolution of these pathogens.

The nonmevalonate isoprenoid biosynthesis or 2-*C*-methyl-d-erythritol 4-phosphate (MEP) pathway has emerged as a promising target for the treatment of infectious diseases, especially those caused by Mtb and *P. falciparum*.^5–7^ Both of these pathogens and many other bacteria utilize the MEP pathway to synthesize isopentenyl pyrophosphate (IPP) and dimethylallyl pyrophosphate (DMAPP), isoprenoids that are essential for synthesis of compounds that make up the cell wall and other essential compounds for pathogen survival. This pathway is particularly attractive due to its absence from mammalian systems, which utilize the mevalonate pathway for isoprenoid biosynthesis. Additionally, the discovery of a phosphonate-containing natural product, fosmidomycin (Fos, Figure 1), has fueled medicinal chemistry efforts due to its potent activity against 1-deoxy-d-xylulose-5-phosphate reductoisomerase (IspC or DXP reductoisomerase), which catalyzes the first committed step in the MEP pathway.^5,8^

**Figure 1:**
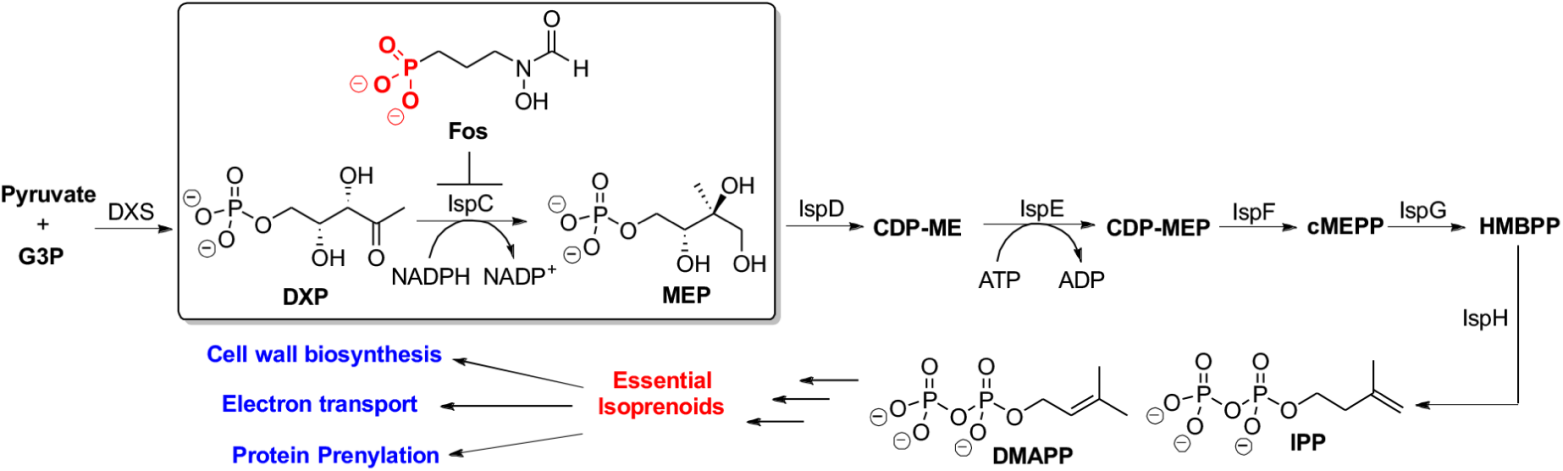
MEP Pathway: The first committed step catalyzed by IspC, synthesis of IPP and DMAPP, and function of isoprenoids in pathogens. Fosmidomycin (Fos) acts as a potent inhibitor of IspC (*Ec*IspC IC_50_ 32–35 nM) and is therefore an attractive target for anti-infective drug design.

Though Fos exhibits potent inhibitory activity (IC_50_ 32–35 nM in *E. coli* IspC), it suffers from poor bioavailability, likely due to its highly charged nature at physiological pH rendering it membrane impermeable.^5,7^ Prodrugs have been investigated as well as α-substituted analogues and reverse chelating analogues to improve membrane permeability and target binding.^9–11^ In recent years, boron containing compounds have been increasingly observed as important contributors to drug discovery and development.^12,13^ The research reported herein focuses on replacement of the phosphonate moiety with a suitable isostere, specifically incorporating boronic acid and benzoxaborole moieties to investigate their ability to mimic in vivo activity of phosphate/phosphonate moieties while improving pharmacological properties.

Boron moieties possess some favorable chemical and physical properties that warrant their investigation as phosphate isosteres.^14^ Boronic acids act as Lewis acids rather than Brønsted acids, so they possess the ability to convert between a neutral trigonal planar geometry and an anionic tetrahedral geometry upon coordination to the empty p-orbital.^15^ Since p*K*_a_ values typically range from 8 to 10, boronic acids will exist predominately in the trigonal planar geometry which would be uncharged at physiological pH.^15^ This could lead to more favorable absorption and membrane permeability compared to that of a phosphate or phosphonate group. Depending on the pH or the chemical environment, boronic acids can readily convert to the anionic tetrahedral geometry that may mimic the shape, size, and charge of a phosphate moiety (Figure 2a). Though the charge density around phosphate is significantly higher than that of the boronic acid, the singly charged boronate species could be sufficiently negative to be recognized as a phosphate/phosphonate group. Additionally, boronic acids are known to react with active site Lewis bases such as serine and lysine to act as dynamic covalent inhibitors.^16–18^ In the case of IspC, the phosphate binding pocket contains several Lewis basic residues, particularly two serine and one lysine, that could be targeted with a boronic acid warhead and could act as a suitable model system for investigating this isosteric relationship (Figure 2b).^5^ Such a binding mode has been observed by Zervosen et al. within the active site of a penicillin-binding protein (PBP) where tight, tricovalent binding was observed to occur between a boronic acid containing molecule and the PBP via two serine residues and a coordinating lysine residue.^16^ This tetravalent, anionic complex about the boron atom was captured by X-ray crystallography (Figure 2c).^16^ This demonstrates the potential of boronic acids to produce tightly bound complexes with proteins with highly polar binding pockets and that IspC could act as a suitable model system to probe their use as phosphate isosteres.

**Figure 2:**
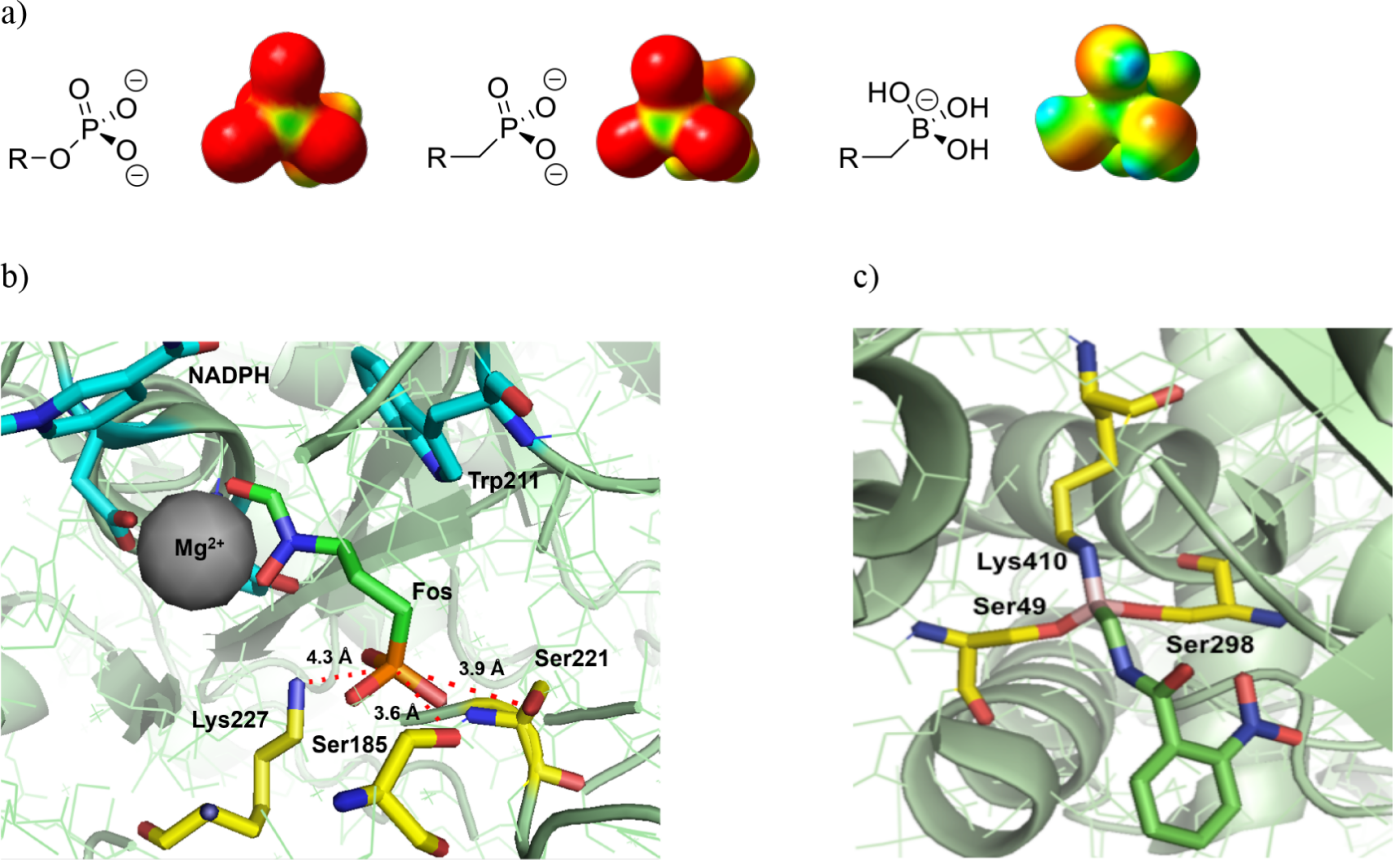
Examining molecular interactions of phosphonates and boronic acids with protein targets. (a) Comparison of geometry and electron density of phosphate, phosphonate, and boronate (made in Gaussian); (b) crystal structure of IspC with fosmidomycin (Fos) bound in the active site; (c) crystal structure of a penicillin-binding protein with a tricovalent boronic acid inhibitor bound.

Given the above considerations, it was hypothesized that boronic acids or benzoxaborole moieties may be utilized as direct, bioisosteric replacements for phosphonate/phosphate moieties. To investigate this hypothesis, we envisioned that the Fos/IspC inhibitor/enzyme pair could be utilized as a model system for biological evaluation of novel, boron-containing fosmidomycin analogs. This system allows us to test for effective isosteric replacement through both an isolated enzyme assay and in whole bacterial cells in culture. The IspC enzyme assay provides a direct measure of the ability of the designed compounds to compete with substrate for the IspC active site thus elucidating the bioisosteric potential of the compounds. In contrast, the whole cell antimicrobial assays can provide a rapid measure of the compounds’ ability to affect microbial growth, typically by entering the cell and disrupting a critical biochemical pathway, i.e., the MEP pathway. Overall, we report the synthesis of a library of boron-containing Fos analogs, evaluation of their activity as IspC inhibitors to probe their effectiveness as bioisosteres for the replacement of the phosphonate group on Fos, and an assessment of the antimicrobial activities of these novel compounds.

## Results and Discussion

### Compound Design Rationale

We rationally designed and synthesized a library of boron-containing analogs of fosmidomycin and FR-900098 (Figure 3). A series of alkyl boronic acids were designed to maintain the important interactions between Fos and IspC as illustrated in Figure 2. With these compounds, the boron-containing moieties (Figure 3b, in blue) are directly replacing the phosphonate groups (Figure 3a, in red) while otherwise maintaining the core structures of Fos or FR-900098 including the retrohydroxamic acid group connected via a short linker. It was decided to vary the alkyl chain length to probe for additional potential active site interactions (**1a-c, 2a-c**). In addition to alkyl boronic acids, we designed trifluoroborate-containing analogs, **3a**–**b**, to ensure the presence of a tetrahedral, anionic species in the IspC inhibition assay. Finally, we envisioned a series of compounds incorporating the benzoxaborole moiety (Figure 3c, **4–10**) to provide more lipophilicity to these compounds and a more reactive boron center due to the introduction of ring strain.^19–22^ As above, the boron centers (blue) are intended to substitute for the phosphonate group (red) while the remainder of the benzoxaborole structure is replacing the linker to either a retrohydroxamic acid or hydroxamic acid group intended to maintain metal chelation in the active site.

**Figure 3:**
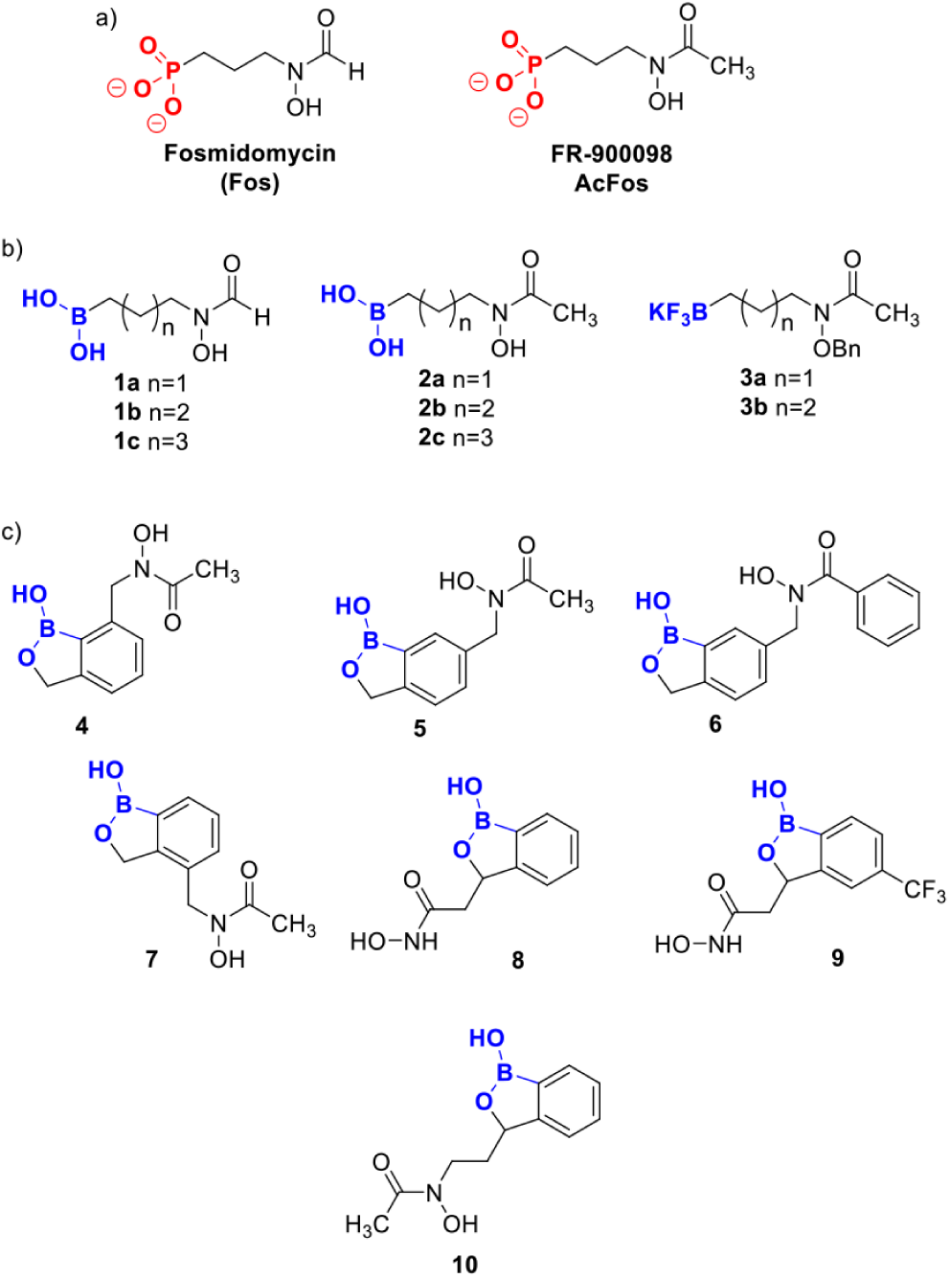
(a) Fosmidomycin and FR-900098. (b) Proposed alkylboronic acid and trifluoroborate libraries. (c) Proposed benzoxaborole library.

### Syntheses

#### Syntheses of Alkylborono Compounds (**1–3**)

The syntheses of the proposed alkyl boronic acid and trifluoroborate libraries were facilitated by boronic acid protecting group interconversion and substitution chemistry developed in our lab.^23^ The synthetic route starts with the two-pot hydroboration reaction with halogenated alkenes **11a**–**c** and treatment with KHF_2_ to produce trifluoroborate salts **12a**–**c** in 60–80% yield (Scheme 1).^24^ These electrophiles were then subjected to nucleophilic substitution with *t*-butyl *N*-(benzyloxy)­carbamate in the presence of sodium hydride followed by our ligand switch conditions to produce **13a**–**c** in 60–81% yield over two steps after chromatography.^23^ The free amine could then be unveiled by removal of the Boc protecting group with TFA in nearly quantitative yields and could be used directly to install the retro-hydroxamic acids. Formylation was achieved with carbonyldiimidazole (CDI) and formic acid to form **14a**–**c** in 73–83% yield over two steps from TFA deprotection. Acetic anhydride was used to synthesize acetyl hydroxamic acids **15a**–**c** in 76–82% yield over two steps from TFA deprotection. The free boronic acids were unveiled via basic hydrolysis of the MIDA boronate with sodium hydroxide and purified via extraction. Alkyl boronic acids **1a**–**c** and **2a**–**c** were then produced by hydrogenation of the free boronic acid intermediates in 43–61% and 18–67% yields, respectively, over two steps. The trifluoroborate library could also be synthesized from **15a**–**b** by hydrolysis of the MIDA boronate followed by reaction with KHF_2_ to produce **3a**–**b** in 39–46% yield over two steps. We maintained the O-benzyl protecting group on **3a**–**b** due to the instability of the benzyl deprotected product.

**Scheme 1:**
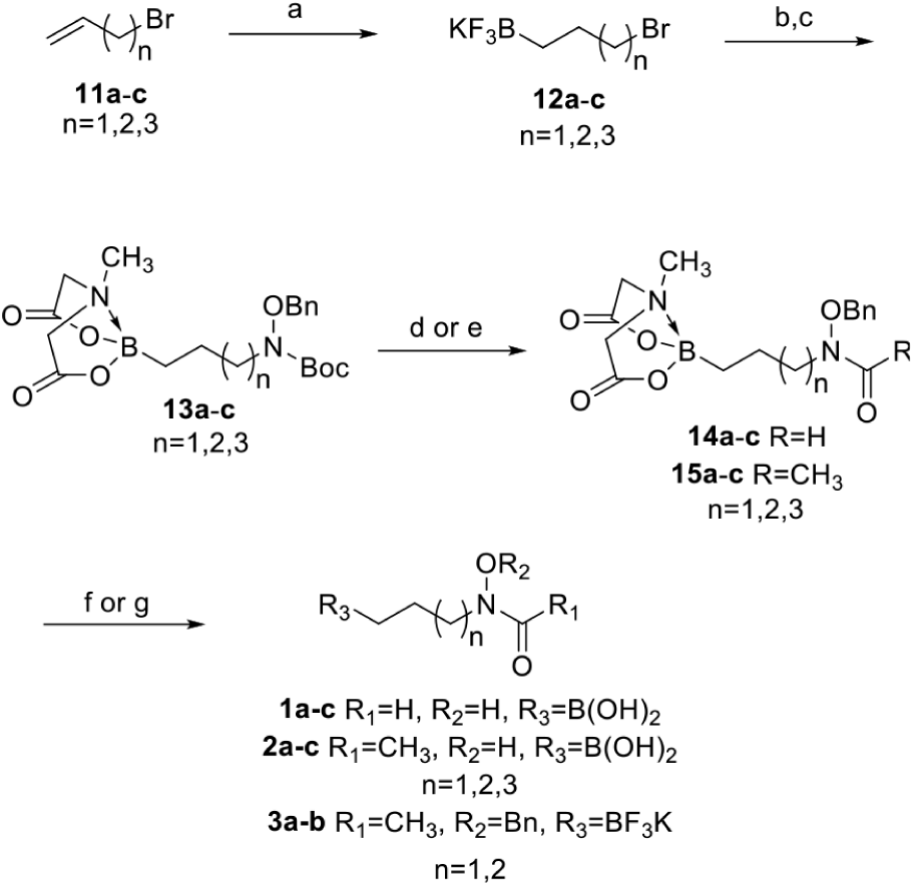
Synthesis of Alkylboronic Acid Library^a^

a Reagents and conditions: (a) BCl_3_, Et_3_SiH, Hexanes, −78 °C then KHF_2_, H_2_O, Et_2_O 60–80% over two steps; (b) *t*-butyl *N*-(benzyloxy)­carbamate, NaH, NaI, DMF, 0–50 °C; (c) *N*-methyliminodiacetic acid, PhCH_3_, DMSO, reflux, 60–81% yield over two steps; (d) trifluoroacetic acid, DCM, rt then formic acid, carbonyldiimidazole, DCM, 73–85% over two steps; (e) trifluoroacetic acid, DCM, rt then acetic anhydride, triethylamine, DCM, rt, 76–82% over two steps; (f) 1 M NaOH, THF, rt then H_2_, Pd/C, MeOH, rt, 18–67% over two steps; (g) 1 M NaOH, THF, rt then KHF_2_, H_2_O, MeOH, rt, 39–46% over two steps.

#### Syntheses of Aryl-Substituted Benzoxaborole Compounds (**4–7**)

The synthesis of the proposed 4-, 6-, and 7-substituted benzoxaborole library was achieved starting from bromo-substituted xylenes **16a**–**c** (Scheme 2). We employed a convenient oxidative bromination procedure with xylenes **16a**–**c** and NBS in the presence of AIBN to produce dibromides **17a**–**c** in yields varying from 38% to quantitative after purification via precipitation or washing with hexanes. We then performed substitution reactions with potassium acetate directly followed by basic hydrolysis of the esters to form the respective diols **18a**–**c** in 18–75% yield over two steps. The diols were then protected as the THP ethers via dihydropyran and p-toluenesulfonic acid to form **19a**–**c** in 62–68% yield after chromatography. The aryl bromides were then subjected to a halogen-lithium exchange/borylation sequence followed by THP deprotection with p-TSA in MeOH to form benzoxaboroles **20a**–**c** in 40–62% yield (Scheme 2).^25,26^ Using a benzoxaborole protection strategy developed in our lab with 3-dimethylamino-1-propanol,^25^ benzoxaboroles **20a**–**c** were protected and then converted to their respective benzylic iodides **21a**–**c** in the presence of sodium iodide and TMSCl in 70–83% yield over two steps. Iodides **21a**-**c** smoothly reacted under substitution conditions with either *N*-((tetrahydro-2*H*-pyran-2-yl)­oxy)­acetamide or *N*-((tetrahydro-2*H*-pyran-2-yl)­oxy)­benzamide (Scheme 2) activated with sodium hydride and the crude material was directly deprotected by p-TSA in methanol. Extraction and purification by column chromatography, crystallization, or solid phase extraction provided **4–7** in 5.7–27% yield over two steps.

**Scheme 2:**
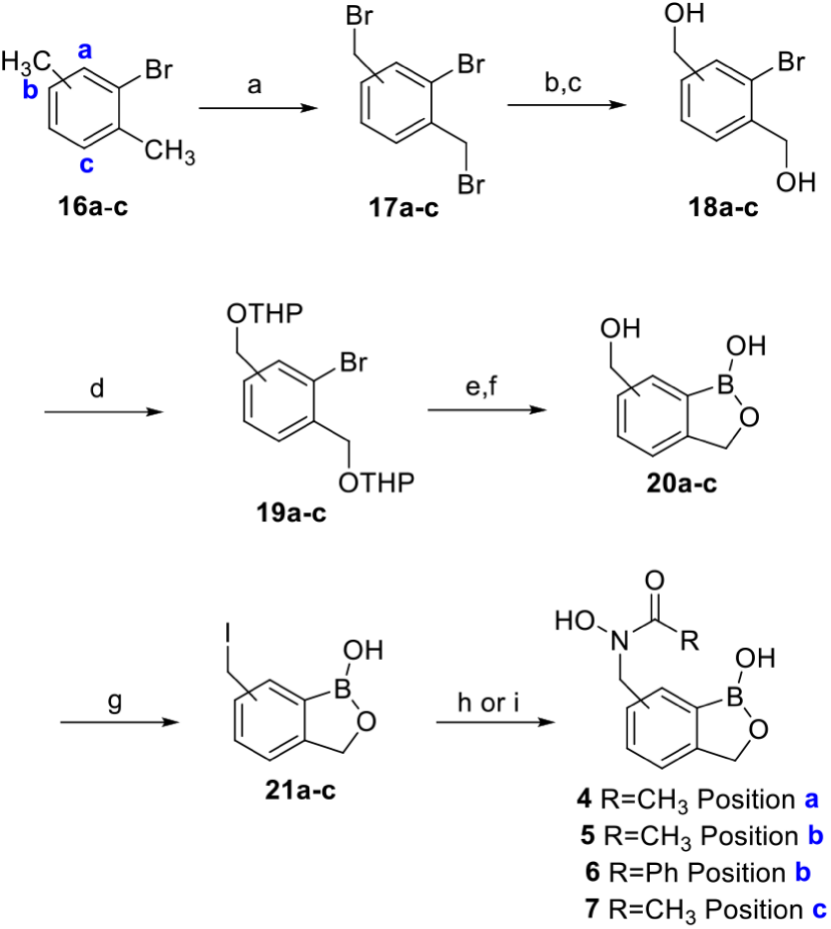
Synthesis of 4-, 6-, and 7-Substituted Benzoxaborole Library^a^

a Reagents and conditions: (a) *N*-bromosuccinimide, CHCl_3_ or CCl_4_, reflux, 38%-quant; (b) Potassium acetate, Me_2_CO:ACN, reflux; (c) KOH or NaOH, THF, 50 °C, 18–75% over two steps; (d) 3,4-dihyropyran, *p*-toluenesulfonic acid, DCM, rt, 62–68%; (e) *n*-BuLi, hexanes, THF, −78 °C then B­(OiPr)_3_, rt; (f) *p*-toluenesulfonic acid, MeOH, rt, 40–62% over three steps; (g) 3-dimethylamino-1-propanol, Na_2_SO_4_, Me_2_CO:Et_2_O, rt then sodium iodide, trimethylsilyl chloride, ACN, 0 °C −rt, 70–83% over two steps; (h) *N*-((tetrahydro-2*H*-pyran-2-yl)­oxy)­acetamide, NaH, DMF, 0 °C then *p*-toluenesulfonic acid, MeOH, rt, 19–27% over two steps; (i) *N*-(tetrahydro-2*H*-pyran-2-yl)­oxy)­benzamide, NaH, DMF, 0 °C then *p*-toluenesulfonic acid, MeOH, rt, 5.7% over two steps.

#### Syntheses of 3-Substituted Benzoxaborole Compounds (**8–10**)

The synthesis of the 3-substituted benzoxaborole analogs began with the synthesis of 2-formylphenyl trifluoroborates **23a** and **23b** (Scheme 3). These aldehydes were prepared from either their the commercially available boronic acid (**22a**) or from 2-bromobenzaldehyde diethyl acetal. These aldehydes were then subjected to condensation with *t*-butyl acetate using LDA followed by deprotection of the trifluoroborate with 0.5 M HCl and SiO_2_ to form the intermediate *t*-butyl esters. Cleavage of the *t*-butyl ester with TFA provided carboxylic acids **24a** and **24b** in 41% and 25% yields over two steps, respectively. Benzoxaboroles **24a** and **24b** were then protected with 3-dimethylamino-1-propanol and subjected to nucleophilic acyl substitution with *O*-tetrahydropyranylhydroxylamine. The crude THP ethers were deprotected with p-toluene sulfonic acid in methanol and majority of impurities were able to be removed by aqueous extraction. Further purification provided **8** and **9** in 27% and 8.5% yield over three steps, respectively.

**Scheme 3:**
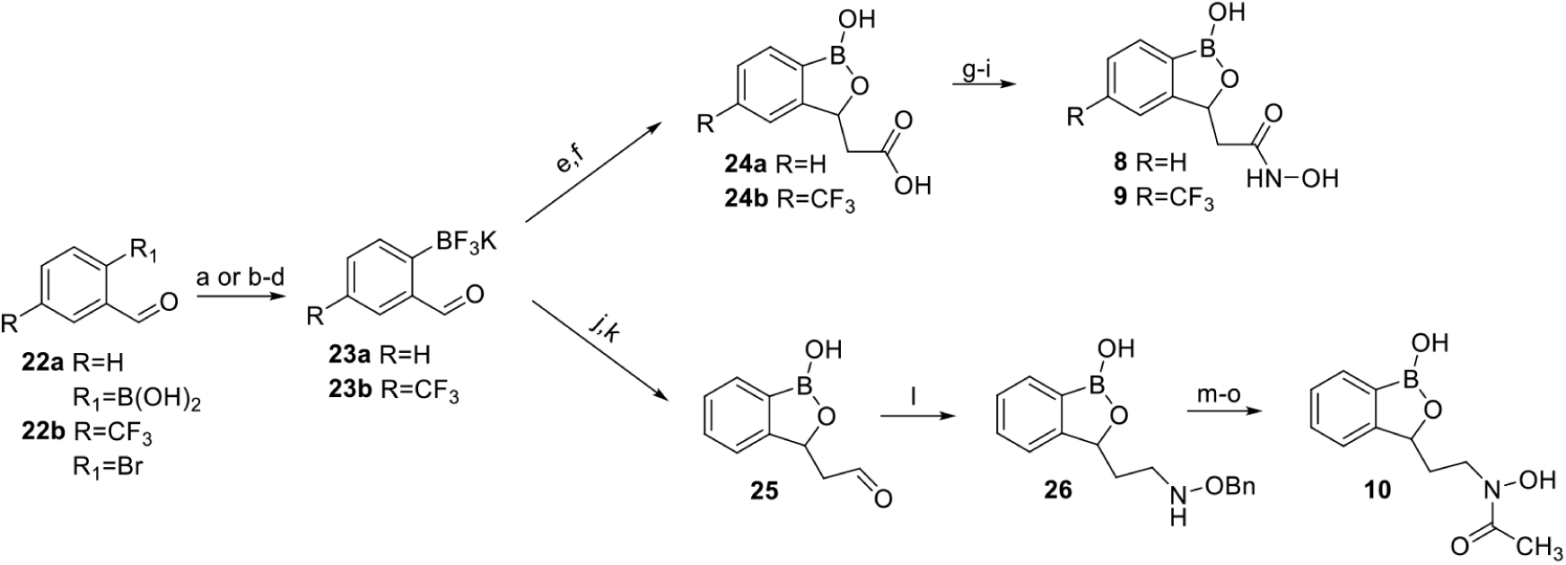
Synthesis of 3-Substituted Benzoxaborole Library^a^

a Reagents and Conditions: (a) KHF_2_, H_2_O, Et_2_O, 95%; (b) CH­(OEt)_3_, p-TSA, EtOH, reflux, 91%; (c) *n*-BuLi, THF, B­(OiPr)_3_, −78 °C-rt; (d) KHF_2_, H_2_O, Et_2_O, 75% over two steps; (e) LDA, *t*-BuOAc, THF, −78 °C-rt then 0.5 M HCl, SiO_2_, rt; (f) TFA, DCM, rt,; 25–41% over two steps; (h) 3-dimethylamino-1-propanol, Na_2_SO_4_, Me_2_CO:Et_2_O, rt; (h) *O*-tetrahydropyranylhydroxylamine, CDI, DCM, rt; (i) p-TSA, MeOH, rt, 8.5–27% over three steps; (j) Bromomethyl-1,3-dioxolane, Mg^0^, THF, reflux-rt, then 1 M HCl, SiO_2_, rt; (k) 3 M HCl, THF, rt, three cycles, 23% over two steps; (l) *O*-benzylhydroxylamine hydrochloride, TEA, THF, rt then PyBH_3_, HCl, MeOH, 0 °C-rt, 76%; (m) 3-dimethylamino-1-propanol, Na_2_SO_4_, Me_2_CO:Et_2_O, rt; (n) Ac_2_O, TEA, DCM, rt; (o) H_2_, Pd/C, MeOH, rt, 36% over three steps.

For the synthesis of **10**, we envisioned the introduction of an aldehyde via a Grignard reaction between **23a** and commercially available bromomethyl-1,3-dioxolane followed by deprotection of the acetal with acid (Scheme 3). We successfully formed a Grignard reagent with bromomethyl-1,3-dioxolane and magnesium which reacted smoothly with **23a** to form an intermediate dioxolane. We were able to selectively deprotect the trifluoroborate to form the benzoxaborole via hydrolysis with 1 M HCl and SiO_2_. The medium proved acidic enough to cyclize to the benzoxaborole without deprotecting the dioxolane, which allowed for removal of unreacted aldehyde with a sodium bisulfite extraction. After this purification, we attempted to deprotect the acetal with 3 M HCl to form aldehyde **25**. Unfortunately, the acetal deprotection proved very troublesome and often resulted in the reisolation of the dioxolane. It required several attempts and purifications in order to isolate reasonable quantities of **25** (23% yield over three steps) to carry forward.

Aldehyde **25** was then subjected to condensation with *O*-benzylhydroxylamine HCl and triethyl amine to form an intermediate imine which was purified by column chromatography prior to reduction. The imine was then reduced with pyridine-borane complex under acidic conditions to form **26** in 76% yield. Using our benzoxaborole protection strategy, we were able to transiently protect **26** with 3-dimethylamino-1-propanol and acetylate with acetic anhydride to form the intermediate O-protected hydroxamate. The substrate was smoothly hydrogenated in the presence of Pd/C to provide **10** in 36% yield over three steps.

### Biological Evaluation of Compounds

#### In Vitro IspC Inhibition Assay

Since this compound library was designed to mimic the competitive inhibitor Fos, this assay provides a direct measure of the ability of the designed compounds to compete with substrate for the IspC active site thus elucidating the bioisosteric potential of the compounds. The assay utilizes IspC from *E. coli* (*Ec*IspC) and consists of a spectrophotometric assay that monitors the consumption of NADPH (Figure 4).^27,28^ Kinetic parameters were optimized at 10 nM *Ec*IspC with a DXP concentration of 100 μM. Under normal conditions, the binding of the phosphate of DXP induces a structural change that results in a closed conformation of IspC that allows for the conversion to MEP.^29,30^ Since Fos contains the phosphonate moiety, it directly mimics the natural substrate and locks the enzyme in the closed conformation.^29^ We therefore allow for this structural change by preincubation of the potential inhibitors and NADPH with IspC before initiating the reaction with DXP to measure the residual enzyme activity.

**Figure 4:**
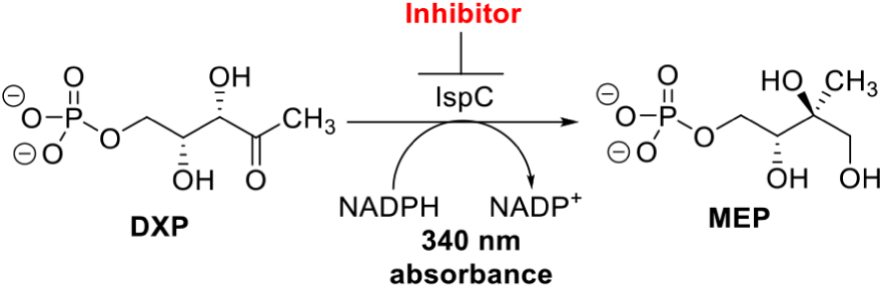
Enzyme inhibition assay: Conversion of DXP to MEP via *Ec*IspC.

Compounds were initially screened at a single, high concentration against *Ec*IspC. We selected 100 μM as the screening concentration, which is 10-fold higher than many known IspC inhibitors, and the rates were compared with the uninhibited DXP reaction control. None of the compounds exhibited any significant inhibition of *Ec*IspC (Table S2). To confirm these screening results, all compounds were retested at various concentrations, up to 400 μM. Fos was utilized as a positive control and we determined an IC_50_ of 25 ± 4 nM which compared well, under comparable conditions, with other reports in the literature.^31–33^ All other compounds resulted in IC_50_ values significantly higher than 100 μM. These results indicate that, at least in the Fos/IspC model system, neither boronic acids nor benzoxaboroles are suitable phosphonate isosteres.

#### *Compound **5** Showed Significant Antimicrobial Activity Against Both* *E. coli* *WT and ΔGlpT Strains*

The compound library was initially screened for antibacterial activity against *E. coli* WT (BW25113) at a high concentration of 100 μg/mL (Figure 5). The alkyl boronic acids were the least active of the compounds exhibiting 8–21% inhibition. We observed a similar result with the trifluoroborate compounds, **3a** and **3b**. We identified a hit compound within the benzoxaborole library, **5**, which exhibited 97% inhibition of *E. coli* WT. None of the remaining benzoxaboroles fully inhibited *E. coli* WT, but we did observe partial inhibition from **6** (44%) and **7** (25%). MIC assays performed on all compounds confirmed the initial screening results (Table S1). Compound **5** was the most potent compound with an MIC of 31 ± 2 μg/mL (Table 1).

**Figure 5:**
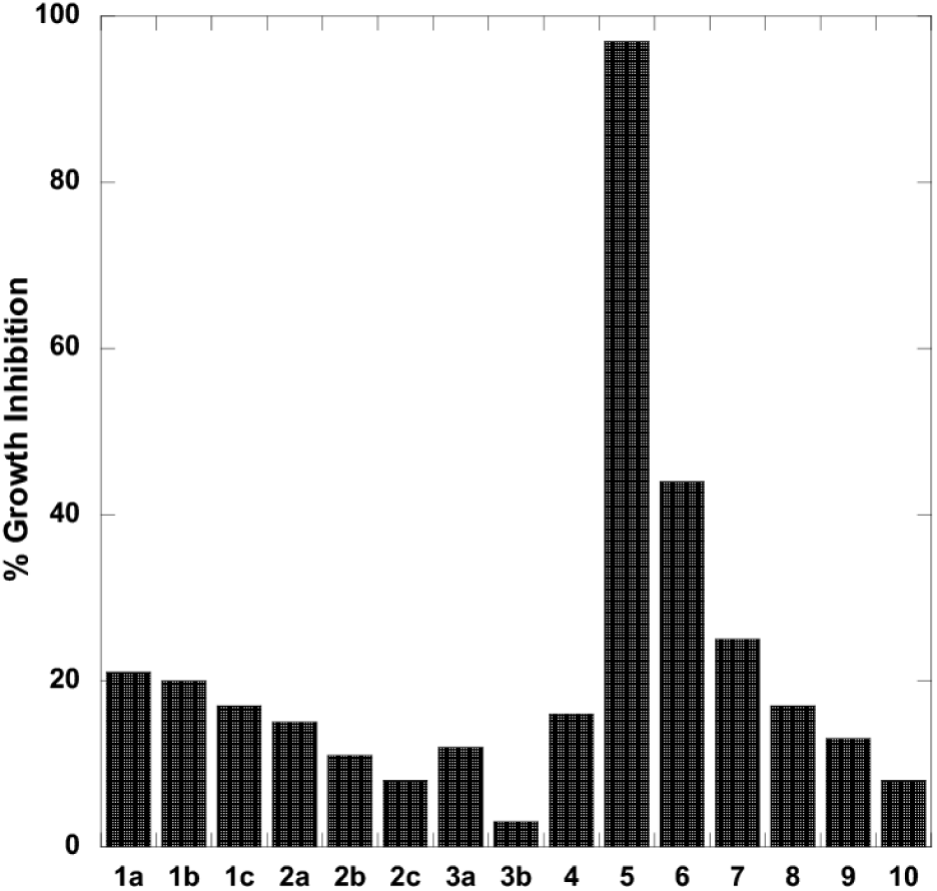
Growth inhibitory effects of compound library against *E. coli* WT at 100 μg/mL.

**Table 1:** Microdilution Assays for Compounds **5** and **6** ^a^

	MIC ± SEM (μg/mL)			
compd	*E. coli* WT^b^	*E. coli* ΔGlpT^c^	*S. aureus* (Wichita)^d^	*S. aureus* (MRSA)^e^
**Fos**	1.0 ± 0.1	>400	N.D.	N.D.
**5**	31 ± 2	30 ± 2	47 ± 2	25 ± 2
**6**	>100	N.D.	40 ± 1	14 ± 4

a N.D.= Not determined.

b BW25113.

c JW2234–2.

d ATCC 29213.

e ATCC 43300.

Since *E. coli* WT possess the GlpT transporter that is necessary for the activity of Fos, we tested **5** against *E. coli* ΔGlpT (JW2234–2) to determine if activity was maintained independently of this transporter. Fosmidomycin was used as a negative control since it is not able to cross the cell membrane by passive diffusion and *E. coli* ΔGlpT is therefore inherently resistant to this agent. As expected, Fos was inactive against *E. coli* ΔGlpT up to 200 μg/mL. To our pleasure, **5** exhibited identical activity against *E. coli* ΔGlpT with an MIC of 30 ± 2 μg/mL which suggests that this compound enters the cell via passive diffusion or an entirely different mechanism (Table 1).

#### Several Benzoxaborole Compounds Exhibited Significant Antimicrobial Activity Against Several ESKAPE Pathogens

To provide an independent evaluation and further characterization of these compounds, the Community for Open Antimicrobial Drug Design (CO-ADD, www.co-add.org) was provided with our compound library for testing against five different ESKAPE pathogens and two fungi.^34^ This evaluation by CO-ADD provided valuable information about these compounds; significant activities are summarized in Table 2 while the full data set has been provided in Tables S3–S12. Consistent with our screening results, compound **5** was identified as the most potent inhibitor of *E. coli*, followed by compound **6**. Of significant interest is the identification of anti-MRSA activities of compounds **4, 5, 6** and **7**. Compounds **4** and **6** exhibited notable activity against *A. baumannii*. Compound **9** was our most potent antimicrobial with near total inhibition of *C. albicans* growth at 32 μg/mL and a reported MIC of 12 μg/mL.

**Table 2:** Summary of Compounds with Significant Antimicrobial Activity Against Select ESKAPE Pathogens as Identified by CO-ADD^a^ ^g^

compd	MRSA^b^	*A. baumannii* ^c^	*E. coli* ^d^	*C. albicans* ^e^
**4**	71%	63%	29%	-
**5**	82%	26%	65%	-
**6**	71%	76%	48%	-
**7**	58%	-	-	-
**9**	-	-	-	>95%^f^

a Data Reported as % Growth Inhibition at 32 μg/ml.

b methicillin-resistant (ATCC 43300).

c *Acinetobacter baumannii* (ATCC 19606).

d *Escherichia coli* (ATCC 25922).

e *Candida albicans* (ATCC 90028).

f MIC = 12 μg/mL.

g “-“inhibition <25%.

An initial toxicity screening of the compounds listed in Table 2 was also completed. The compounds were screened at 32 μg/mL for cytotoxicity against a human cell line (HEK293) and for hemolysis of red blood cells. Stringent thresholds for both cytotoxicity and hemolytic activity, <50% and <10% maximal response respectively, were utilized. These stringent thresholds were established to flag any partial toxicity, which would show well-defined toxicity (CC50 or HC10) at higher concentrations. All of our submitted compounds exhibited less than 10% response in this screening (Tables S10 and S11).

In order to provide independent confirmation of the results provided by CO-ADD, we chose to evaluate the 6-substituted benzoxaboroles **5** and **6** against MRSA and a susceptible strain of *S. aureus*. Compounds **5** and **6** were chosen, in part, due to their structural similarity, differing by only the substitution of a methyl vs phenyl group, respectively, on the retrohydroxamide linker. With the assistance of Dr. Jason Heindl to enable BSL2 work, we were able to confirm the results obtained by CO-ADD for **5** and **6**. MRSA growth inhibition MICs were determined to be 25 ± 2 μg/mL and 14 ± 4 μg/mL, respectively (Table 1). Interestingly, **6** exhibited at least 2-fold more potent anti-MRSA activity than against susceptible *S. aureus*. These results are of particular interest since **5** provides broad spectrum activity with about equal growth inhibition against *S. aureus*, MRSA, *E. coli* WT and *E. coli* ΔGlpT. On the other hand, **6** is more potent than **5** against MRSA while having significantly less activity against *E. coli* thus providing an opportunity to develop a selective anti-MRSA agent.

#### MEP Pathway Inhibition Evaluation

It is known in the literature that the charge of the phosphonate group of Fos is important for binding within the phosphate binding pocket of IspC and it is possible that these boron functionalities did not possess sufficient charge for inhibition.^35^ Previously reported attempts at replacing the phosphonate moiety, even with charged species, resulted in loss of affinity for IspC.^36,37^ Though we did not observe inhibition of *Ec*IspC with any of the antimicrobial compounds, we could further confirm that no part of the MEP pathway is involved with their mechanism of action via an IPP rescue assay with *E. coli* as previously reported in our lab.^33^ Given that **5** displayed the most potent inhibition of *E. coli* growth, it was chosen for this study.

In the case that **5** inhibits bacterial growth via any part of the MEP pathway, supplementing the medium with IPP would rescue bacterial growth and lead to a significant increase to its apparent MIC. If **5** works through another mechanism of action, supplementing with IPP would have no effect on growth inhibition and MIC. As expected, the MIC of Fos increases 5-fold when growth medium is supplemented with IPP and growth seems to be partially rescued overall (Table 3 and Figure S66). In the case of **5**, we did not observe any significant difference in the growth curves or MIC in the presence of IPP supplementation (Table 3 and Figure S67). Finally, it is known that *S. aureus* utilizes the mevalonate pathway, rather than the MEP pathway, for the synthesis of isoprenoids.^38^ Therefore, the combined results of the *Ec*IspC inhibitory assays, IPP rescue experiments, and the anti-*S. aureus* activities strongly suggest that **5** acts independently of IspC and MEP pathway inhibition.

**Table 3:** MICs of Fos and **5** Against *E. coli* WT with and without IPP

	MIC ± SEM (μg/mL)	
compd	*E. coli* WT (−) IPP	*E. coli* WT (+) IPP
**Fos**	1 ± 0.1	5 ± 0.4
**5**	31 ± 2	35 ± 4

#### Structure–Activity Relationship Considerations for Active Compounds

Our results indicate that benzoxaboroles **4**, **5**, **6**, and **7** with benzylic retro-hydroxamic acid moieties are important to antibacterial activity while a methylenehydroxamide at the 3-position (**9**) provides activity against yeast. Within the antibacterial compounds, there appears to be a difference in potency based on the position of the retro-hydroxamic acid. Our findings combined with results from CO-ADD show that compounds substituted at the 6-position (**5, 6**) seem to possess the most potent anti-MRSA activity, followed closely by compound **4** substituted at the 7-position. When considering the identity of the substituent on the retrohydroxamide in **5** vs **6**, the substitution at this position may provide an avenue for enhancing species selectivity. Overall, these results provide intriguing structure–activity relationship data that will inform the design of a targeted library of compounds to improve potency and selectivity against MRSA and other bacterial pathogens.

## Conclusions

The initial drug design goal was to explore a potential isosteric relationship between phosphate/phosphonate moieties and boron-containing moieties. Using fosmidomycin and IspC as a suitable model system, it was hypothesized that replacement of the phosphonate in Fos with boron-containing moieties could lead to comparable biological activity while potentially acting as covalent warheads within the binding site of IspC. Enzyme inhibition studies conclusively demonstrated that the synthesized boronic acids and benzoxaboroles do not function as effective phosphate/phosphonate bioisosteres for IspC inhibition. Although these compounds were originally designed based on their predicted interaction with IspC binding site, analogous to fosmidomycin, no such interaction was evidenced. Therefore, either the compounds do not bind to IspC or such binding does not inhibit IspC activity.

Despite the absence of IspC inhibition, this work has identified several novel benzoxaborole compounds with significant antimicrobial properties against important pathogens. Compound **9** demonstrated potent activity against *Candida albicans* with an MIC of 12 μg/mL, while compounds **5** and **6** exhibited substantial inhibition of methicillin-resistant *Staphylococcus aureus* (MRSA) with MICs of 25 μg/mL and 14 μg/mL, respectively. Notably, both compounds **5** and **6** showed greater potency against MRSA than against methicillin-susceptible *S. aureus*, suggesting potential selectivity for resistant strains. Compound **5** also exhibited activity against *E. coli* WT and *E. coli* ΔGlpT with nearly identical MICs (31 μg/mL and 30 μg/mL, respectively). This consistent activity in the glycerol-3-phosphate transporter-deficient strain strongly suggests that **5** enters bacterial cells via passive diffusion, overcoming a key limitation of phosphonate-based antimicrobials like fosmidomycin. These findings position **5** as a potential broad spectrum antibacterial lead, while **6** represents a potential selective anti-MRSA lead.

Mechanistic studies, including IspC inhibition assays and IPP rescue experiments, confirm that these compounds act through targets distinct from the MEP pathway. This represents an unexpected but valuable outcome, as it has led to the discovery of novel antimicrobial scaffolds with promising activity profiles. In order to explain the observed antimicrobial activities, there must be one or more targets to which these compounds bind resulting in the observed phenotypes. Future work will focus on identifying the mechanism of action of these compounds and optimizing their structures to increase potency and selectivity. Structure–activity relationship data suggest that the position of substitution on the benzoxaborole scaffold and the identity of the retrohydroxamide substituent significantly influence antimicrobial activity and selectivity. These insights will guide the development of targeted compound libraries to further explore these promising antimicrobial leads.

## Methods

### General Methods

Moisture- and/or air-sensitive reactions were performed under an argon atmosphere in flame- or oven-dried glassware. Solvents and reagents were purchased from commercial sources and used without further purification. For reactions that required dry solvent, the respective solvent was dried over 3 Å molecular sieves for a minimum of 72 h as per literature procedure.^39^ All other reactions were performed under ambient air at the specified temperature and time. Concentration refers to solvent removal on a rotary evaporator and drying refers to further evacuation with a two-stage mechanical pump. NMR spectra data were obtained on a Bucker Avance III 400 MHz NMR spectrometer. Chemical shifts are reported in parts per million (ppm) against the tetramethylsilane (TMS) standard or residual solvent signal. ^11^B NMR spectra are reported in ppm using BF_3_·OEt_2_ as an external standard. ^13^C NMR resonances next to boron are typically not observed due to quadrupolar relaxation. Positive mode mass spectra were obtained on a Thermo-Fisher Exactive Orbitrap Mass Spectrometer using either Matrix-Assistant Inlet Ionization (MAII),^40^ Atmospheric Solid Analysis Probe (ASAP),^41^ or Electrospray Ionization (ESI). Negative mode mass spectra were performed by the Biological and Small Molecule Mass Spectrometry Core at the University of Tennessee or the Mass Spectrometry Facility at the University of Pennsylvania. Purity of all compounds was determined by ^1^H NMR with dimethyl sulfone (99.4%) as an internal standard in either D_2_O or CD_3_OD.^42^ An *E. coli* BL21 strain harboring an IPTG inducible plasmid for the overexpression of His-tagged *E. coli* IspC was provided by the Freel Meyers lab at Johns Hopkins University. Overexpression and purification were performed according to literature procedures.^27,28^ Kinetic assays were performed in 1 mL quartz cuvettes and absorbance was recorded at 340 nm on an Agilent Cary 300 UV–visible spectrometer. Minimum inhibitory concentrations (MICs) were determined by microdilution assay^43^ in Corning 96-well plates with flat bottoms and low evaporation lids that were read on either a BioTek Synergy H1 microplate reader or a Molecular Devices SpectraMax Plus 384 microplate reader. Initial OD_600_ readings were obtained on an Eppendorf BioPhotometer. *E. coli* strains BW25113 (*E. coli* WT, CGSC# 7636) and JW2234–2 (*E. coli* ΔGlpT, CGSC# 11875) were purchased from the Coli Genetic Stock Center (CGSC) at Yale University. *S. aureus* strains ATCC 29213 (*S. aureus* Wichita, Methicillin susceptible) and ATCC 43300 (*S. aureus* F-182, MRSA) were purchased from ATCC. Compounds were dissolved in sterile DMSO and diluted to 2 mg/mL to make a final solvent ratio of 90% ddH_2_O:10% DMSO. Serial dilutions were performed with 10% DMSO (final assay concentration 1% DMSO). Antimicrobial screening was also performed by the Community for Open Antimicrobial Drug Design (CO-ADD), funded by the Wellcome Trust (UK) and The University of Queensland (Australia).^34^

### Synthetic Methods

#### Synthesis of Haloalkyl Trifluoroborate Salts (**12a–c**)

All trifluoroborate salts were synthesized by our published two-pot synthesis method.^23^ To a flame-dried round-bottom flask and stir bar was added halo-alkene (1 equiv) and triethylsilane (1.04 equiv) on a dry ice/acetone bath). To the stirring mixture was slowly added boron trichloride (BCl_3_) (1 M solution in hexane, 1.1 equiv) via syringe and allowed to stir for 30 min. The mixture was warmed to rt and stirred for 2 h. When complete, the reaction was placed on an ice bath, quenched with water (∼10 mL), and stirred for 10 min. The mixture was transferred to a separatory funnel along with the addition of ether (10 mL). Phases were separated and aqueous phase was extracted with Et_2_O (3 × 20 mL). Combined organics were dried over Na_2_SO_4_, filtered, and concentrated on a rotary evaporator. Et_2_O (15–25 mL) and potassium hydrogen difluoride (KHF_2_) (5 equiv) were added to the crude residue and stirred at rt with the slow addition of water (2–3 mL) over 1 h. Insoluble salts were filtered and washed with acetone. The filtrate was concentrated to dryness and then dissolved in a minimal volume of acetone and added to Et_2_O to promote crystallization. Precipitate was filtered, washed with Et_2_O, and dried *in vacuo* to afford trifluoroborate salts **12a–c**.

#### Potassium 3-Bromopropyl Trifluoroborate (**12a**)

Allyl bromide (1.5 mL, 17.3 mmol), triethylsilane (3.0 mL, 18.1 mmol), and BCl_3_ (1 M solution in hexanes, 19 mL, 19 mmol) were stirred together and quenched with water. Stirring in the presence of water and KHF_2_ (5.60 g, 71.7 mmol) followed by crystallization with Et_2_O afforded **12a** (2.76 g, 70%) as a white fluffy solid. ^1^H NMR (400 MHz, acetone-d_6_): δ 3.41 (t, 2H, *J* = 7.6 Hz), 1.72–1.63 (m, 2H), 0.00 to −0.11 (br, 2H); ^13^C NMR (100 MHz, acetone-d_6_): δ 38.4, 30.6. ^11^B NMR (128 MHz, Acetone-*d*_6_) δ 5.2. HRMS (ESI) *m*/*z* calcd for C_3_H_6_BF_3_Br (M – K)^−^ 188.9698, found: 188.9692.

#### Potassium 4-Bromobutyl Trifluoroborate (**12b**)

4-Bromo-1-butene (1.7 mL, 16.7 mmol), triethylsilane (2.80 mL, 17.5 mmol), and BCl_3_ (1 M solution in hexanes, 18 mL, 18 mmol) were stirred together and quenched with water. Stirring in the presence of water and KHF_2_ (5.10 g, 65.3 mmol) followed by crystallization with Et_2_O afforded **12b** (2.63 g, 65%) as a white fluffy solid. ^1^H NMR (400 MHz, acetone-d_6_): δ 3.46 (t, 2H, *J* = 7.0 Hz), 1.76–1.68 (m, 2H), 1.26–1.19 (m, 2H), 0.00 to −0.08 (m, 2H); ^13^C NMR (100 MHz, acetone-d6): δ 36.6, 34.9, 24.2. ^11^B NMR (128 MHz, acetone-d_6_) δ 4.6. HRMS (ESI) *m*/*z* calcd for C_6_H_11_BNF_2_Br [M – (KF) + (CH_3_CN)]^−^ 225.0136, found 225.0127.

#### Potassium 5-Bromopentyl Trifluoroborate (**12c**)

5-Bromo-1-pentene (2.3 mL, 19.4 mmol), triethylsilane (2.30 mL, 20.6 mmol), and BCl_3_ (1 M solution in hexanes, 21 mL, 21 mmol) were stirred together and quenched with water. Stirring in the presence of water and KHF_2_ (6.01 g, 76.9 mmol) followed by crystallization with Et_2_O afforded **12c** (2.98 g, 60%) as a white fluffy solid. ^1^H NMR (400 MHz, acetone-d_6_): δ 3.48 (t, 2H, *J* = 6.8 Hz), 1.78–1.721 (m, 2H), 1.33–1.27 (m, 2H), 1.16–1.11 (m, 2H), −0.04 to −0.09 (m, 2H); ^13^C NMR (100 MHz, acetone-d_6_): δ 34.4, 33.3, 31.8, 24.7. ^11^B NMR (128 MHz, acetone-d_6_) δ 4.5. HRMS (ESI) *m*/*z* calcd for C_7_H_13_BNF_2_Br [M – (KF) + (CH_3_CN)]^−^ 239.0287, found 239.0292.

### General Procedure for Carbamate Substitution and Ligand Replacement with MIDA (**13a–c**)

Pure NaH (1.3 equiv) and NaI (10 mol %) were weighed into a round-bottom flask in a glovebox under a nitrogen atmosphere. After removing from the glovebox, DMF was added, followed by *t*-butyl *N*-(benzyloxy)­carbamate (1.1 equiv) dissolved in DMF at 0 °C. The mixture was stirred at this temperature until evolution of H_2_ gas ceased (∼10 min), then the potassium haloalkyltrifluoroborate salt (1.0 equiv) dissolved in DMF was added. The reaction was stirred at 40 °C overnight. The reaction was filtered, concentrated, and the crude product was carried through the MIDA ligand replacement without further purification. To the round-bottom flask containing the crude trifluoroborate salt was added MIDA (1.5–2.0 equiv) and SiO_2_ (5–8 equiv). Toluene and DMSO were added and the flask was fitted with a Dean–Stark trap charged with toluene. The reaction was refluxed overnight, filtered to remove insoluble solids, and aqueous sat’d ammonium chloride (NH_4_Cl) was added to the filtrate. The organic layer was separated, and the aqueous layer was extracted 3× with EtOAc. The combined organics were washed with brine, dried over Na_2_SO_4_, filtered, and concentrated to yield the crude product which was purified via column chromatography (SiO_2_, EtOAc) to afford pure MIDA boronates **13a**–**c**.

#### 3-(*tert*-Butyl *N*-(Benzyloxy)­carbamate)­propyl Boronic Acid MIDA Ester (**13a**)

Use of the general procedure with **12a** (1.50 g, 6.56 mmol) provided pure MIDA boronate **13a** (2.08 g, 76%) as a white, glassy solid. mp (transition): 73.1–75.0 °C. ^1^H NMR (400 MHz, CDCl_3_) δ 7.43–7.28 (m, 5H), 4.80 (s, 2H), 3.88 (d, *J* = 16.7 Hz, 2H), 3.62 (d, *J* = 16.6 Hz, 2H), 3.52–3.41 (m, 2H), 2.77 (s, 3H), 1.72–1.60 (m, 2H), 1.48 (s, 8H), 0.59–0.50 (m, 2H). ^13^C NMR (100 MHz, CDCl_3_) δ 167.74, 156.61, 135.67, 129.59, 128.69, 128.61, 81.31, 76.81, 62.05, 51.52, 45.85, 28.49, 21.84. ^11^B NMR (128 MHz, CDCl_3_): δ 13.0. HRMS (ASAP) *m*/*z* calcd for C_20_H_30_BN_2_O_7_ (M+H)^+^ 421.2141, found 421.2137.

#### 4-(*tert*-Butyl *N*-(Benzyloxy)­carbamate)­butyl Boronic Acid MIDA Ester (**13b**)

Use of the general procedure with **12b** (1.51 g, 6.21 mmol) provided pure MIDA boronate **13b** (2.19 g, 81%) as a white, glassy solid. mp: 117.1–118.2 °C. ^1^H NMR (400 MHz, CDCl_3_) δ 7.44–7.28 (m, 5H), 4.81 (s, 2H), 3.82 (d, *J* = 16.4 Hz, 2H), 3.61 (d, *J* = 16.4 Hz, 2H), 3.43 (t, *J* = 7.1 Hz, 2H), 2.80 (s, 3H), 1.66 (m, 2H), 1.49 (s, 9H), 1.43–1.31 (m, 2H), 0.64–0.56 (m, 2H). ^13^C NMR (100 MHz, CDCl_3_) δ 167.32, 156.65, 135.70, 129.56, 128.67, 128.60, 81.32, 76.90, 61.97, 49.20, 45.75, 30.08, 28.48, 21.24. ^11^B NMR (128 MHz, CDCl_3_): δ 13.3. HRMS (ASAP) *m*/*z* calcd for C_21_H_32_BN_2_O_7_ (M+H)^+^ 435.2297, found 435.2292.

#### 5-*(tert*-Butyl *N*-(Benzyloxy)­carbamate)­pentyl Boronic Acid MIDA Ester (**13c**)

Use of the general procedure with potassium **12c** (2.20 g, 8.57 mmol) provided pure MIDA boronate **13c** (2.28 g, 60%) as a white, glassy solid. mp: 129.5–130.5 °C. ^1^H NMR (400 MHz, CDCl_3_) δ 7.44–7.28 (m, 5H), 4.81 (s, 2H), 3.83 (d, *J* = 16.4 Hz, 2H), 3.64 (d, *J* = 16.4 Hz, 2H), 3.41 (t, *J* = 7.2 Hz, 2H), 2.84 (s, 3H), 1.64–0.55 (m, 2H), 1.49 (s, 9H), 1.40–1.30 (m, 4H), 0.63–0.54 (m, 2H). ^13^C NMR (100 MHz, CDCl_3_) δ 167.25, 156.69, 135.77, 129.53, 128.64, 128.59, 81.29, 76.95, 62.04, 49.52, 45.78, 29.84, 28.49, 26.90, 23.71. ^11^B NMR (128 MHz, CDCl_3_): δ 13.4. HRMS (ASAP) *m*/*z* calcd for C_22_H_34_BN_2_O_7_ (M+H)^+^: 449.2454, found 449.2456.

### General Procedure for Boc Deprotection and Formylation (**14a–c**)

In a round-bottom flask, MIDA ester (1 equiv) was dissolved in anhydrous DCM (20–30 mL) and TFA (10 equiv) was added via syringe at rt. The mixture was stirred for 1–2 h at rt and then cooled to 0 °C. The reaction was carefully quenched with sat’d NaHCO_3_ until the pH reached just above 7 with pH strips. The mixture was transferred to a separatory funnel and extracted with DCM (3 × 50 mL). The combined organics were washed with brine, dried over Na_2_SO_4_, and concentrated to afford the intermediate amine as a hygroscopic solid. The amine was used directly without purification. To a dry round-bottom flask was added 1,1’-carbonyldiimidazole (CDI, 2.0 equiv) and a slurry was made with anhydrous DCM (10–20 mL). Formic acid (3.0 equiv) was slowly added at rt over 10 min and the mixture was stirred for 15 min at rt. The intermediate amine was dissolved in anhydrous DCM (10–15 mL) and added to the reaction in one portion. The reaction was stirred for 24 h at rt and then transferred to a separatory funnel. Water (50 mL) and DCM (50 mL) were added and the mixture was extracted. The aqueous layer was extracted further with DCM (2 × 50 mL) and the combined organics were washed with 0.5 M HCl and brine, dried over Na_2_SO_4_, and concentrated. Minor impurities were removed by suspending the hygroscopic solid several times in Et_2_O and decanting the solvent. Drying under high vacuum afforded **14a–c.**

#### 3-(*N*-Benzyloxy formamido)­propyl Boronic Acid MIDA Ester (**14a**)

MIDA Ester **13a** (2.0 g, 4.75 mmol), DCM (20 mL), and TFA (3.6 mL, 5.39 g, 47.2 mmol) were stirred for 1 h. Intermediate amine (1.52 g, 4.74 mmol, CDI (1.54 g, 9.55 mmol), formic acid (0.55 mL, 671 mg, 14.5 mmol), and DCM (10 mL) were stirred, worked up, and dried under high vacuum to afford **14a** (1.37 g, 83% over two steps) as a hygroscopic, white solid. NMR showed a mixture of rotational isomers. ^1^H NMR (400 MHz, CDCl_3_) δ 8.20 + 7.95 (2*s*, 1H), 7.48–7.31 (m, 5H), 4.95 + 4.83 (2*s*, 1H), 3.84 (d, *J* = 16.4 Hz, 2H), 3.67 (d, *J* = 16.4 Hz, 2H), 3.62 + 3.35 (2*m*, 2H), 2.84 (s, 3H), 1.83–1.68 (m, 2H), 0.64–0.46 (m, 2H). ^13^C NMR (100 MHz, CDCl_3_) δ 168.49, 163.08, 158.35, 134.90, 134.41, 129.57, 129.14, 128.80, 77.59, 76.00, 68.19, 65.85, 61.92, 60.41, 50.36, 46.31, 45.88, 22.00, 21.66, 15.30. ^11^B (128 MHz, CDCl_3_) δ 13.4. HRMS (ASAP) *m*/*z* calcd for C_16_H_22_BN_2_O_6_ (M+H)^+^: 349.1565, found 349.1569.

#### 4-(*N*-Benzyloxy formamido)­butyl Boronic Acid MIDA Ester (**14b**)

MIDA Ester **13b** (2.53 g, 5.82 mmol), DCM (25 mL), and TFA (4.4 mL, 6.55 g, 57.4 mmol) were stirred for 1 h. Intermediate amine (1.85 g, 5.53 mmol), CDI (1.67 g, 10.3 mmol), formic acid (0.55 mL, 671 mg, 14.5 mmol), and DCM (15 mL) were stirred, worked up, and dried under high vacuum to afford **14b** (1.74 g, 86% over two steps) as a hygroscopic, white solid. NMR showed a mixture of rotational isomers. ^1^H NMR (400 MHz, CDCl_3_) δ 8.15 + 7.90 (2*s*, 1H), 7.42–7.30 (m, 5H), 4.91 + 4.81 (2*s*, 1H), 3.97 (d, *J* = 16.8 Hz, 2H), 3.68 (d, *J* = 16.8 Hz, 2H), 3.56 + 3.32 (2*s*, 2H), 2.81 (s, 3H), 1.71–1.59 (m, 2H), 1.39–1.25 (m, 2H), 0.63–0.50 (m, 2H). ^13^C NMR (100 MHz, CDCl_3_) δ 168.46, 134.50, 129.60, 129.21, 128.87, 77.73, 67.84, 61.98, 43.69, 39.02, 29.50, 24.10, 23.06, 21.12, 14.14. ^11^B (128 MHz, CDCl_3_) δ 12.9. HRMS (ESI) *m*/*z* calcd for C_17_H_24_BN_2_O_6_ (M+H)^+^ 363.1722, found 363.1732.

#### 5-(*N*-Benzyloxy formamido)­pentyl Boronic Acid MIDA Ester (**14c**)

MIDA Ester **13c** (3.00 g, 6.69 mmol), DCM (30 mL), and TFA (5.2 mL, 7.74 g, 67.8 mmol) were stirred for 2 h. Intermediate amine (2.33 g, 6.69 mmol), CDI (2.16 g, 13.3 mmol), formic acid (0.75 mL, 915 mg, 21.7 mmol), and DCM (20 mL) were stirred, worked up, and dried under high vacuum to afford **14c** (0.84 g, 73% over two steps) as a hygroscopic, white solid. NMR showed a mixture of rotational isomers. ^1^H NMR (400 MHz, CDCl_3_) δ 8.15 + 7.90 (2*s*, 1H), 7.45–7.27 (m, 5H), 4.91 + 4.80 (2*s*, 2H), 3.97 (d, *J* = 16.8 Hz, 2H), 3.68 (d, *J* = 16.8 Hz, 2H), 3.53 + 3.29 (2*s*, 2H), 2.83 (s, 3H), 1.61 (s, 2H), 1.32 (s, 4H), 0.54 (t, *J* = 7.2 Hz, 2H). ^13^C NMR (100 MHz, CDCl_3_) δ 168.40, 134.40, 129.51, 129.15, 128.81, 77.67, 61.93, 45.85, 43.81, 29.48, 26.50, 23.52. ^11^B (128 MHz, CDCl_3_) δ 13.1. HRMS (ESI) *m*/*z* calcd for C_18_H_26_BN_2_O_6_ (M+H)^+^ 377.1878, found 377.1893.

### General Procedure for Boc Deprotection and Acylation (**15a–c**)

In a round-bottom flask, MIDA Ester **13a-c** (1 equiv) was dissolved in anhydrous DCM (25–30 mL) and TFA (10 equiv) was added via syringe at rt. The mixture was stirred for 1–2 h at rt and then cooled to 0 °C. The reaction was carefully quenched with sat’d NaHCO_3_ until the pH reached just above 7 with pH strips. The mixture was transferred to a separatory funnel and extracted with DCM (3 × 50 mL). The combined organics were washed with brine, dried over Na_2_SO_4_, and concentrated to afford the intermediate amine as a hygroscopic solid. The amine was used directly without purification. The intermediate amine was directly dissolved in anhydrous DCM (25–30 mL) in a round-bottom flask and TEA (1.1 equiv) and Ac_2_O (1.5–1.6 equiv) were added at rt. The mixture was stirred for 10–16 h and then quenched with sat’d NH_4_Cl (30 mL). The mixture was transferred to a separatory funnel and the aqueous layer was extracted with DCM (2 × 50 mL). The combined organics were washed with brine, dried over Na_2_SO_4_, and concentrated to a hygroscopic solid. Minor impurities were removed by trituration several times with Et_2_O. Drying under high vacuum afforded **15a–c**.

#### 3-(*N*-Benzyloxy acetamido)­propyl Boronic Aacid MIDA Ester (**15a**)

MIDA Ester **13a** (2.50 g, 5.94 mmol), DCM (25 mL), and TFA (4.6 mL, 6.89 g, 60.4 mmol) were stirred for 1 h. Intermediate amine (1.81 g, 5.65 mmol), TEA (0.91 mL, 660 mg, 6.52 mmol), Ac_2_O (0.90 mL, 972 mg, 9.52 mmol), and DCM (10 mL) were stirred, worked up, and dried under high vacuum to afford **15a** (1.65 g, 76% over two steps) as a hygroscopic, white solid. ^1^H NMR (400 MHz, CDCl_3_) δ 7.46–7.29 (m, 5H), 4.80 (s, 2H), 3.97 (d, *J* = 16.8 Hz, 2H), 3.69 (d, *J* = 16.8 Hz, 2H), 3.64 (t, *J* = 7.6 Hz, 2H), 2.83 (s, 3H), 2.05 (s, 3H), 1.76–1.64 (m, 2H), 0.59–0.50 (m, 2H). ^13^C NMR (100 MHz, CDCl_3_) δ 168.24, 134.59, 129.34, 129.03, 128.82, 76.30, 62.01, 47.66, 45.92, 21.81, 20.65. ^11^B (128 MHz, CDCl_3_) δ 13.2. HRMS (ASAP) *m*/*z* calcd for C_17_H_24_BN_2_O_6_ (M+H)^+^: 363.1722, found 363.1722.

#### 4-(*N*-Benzyloxy acetamido)­butyl Boronic Acid MIDA Ester (**15b**)

MIDA Ester **13b** (3.05 g, 7.02 mmol), DCM (30 mL), and TFA (5.3 mL, 7.9 g, 69.1 mmol) were stirred for 1 h. Intermediate amine (2.34 g, 7.02 mmol), TEA (1.02 mL, 740 mg, 7.31 mmol), Ac_2_O (1.20 mL, 1.29 g, 12.7 mmol), and DCM (30 mL) were stirred, worked up, and dried under high vacuum to afford **15b** (1.65 g, 76% over two steps) as a hygroscopic, white solid. ^1^H NMR (400 MHz, CDCl_3_) δ 7.41–7.32 (m, 5H), 4.80 (s, 2H), 3.93 (d, *J* = 16.6 Hz, 2H), 3.69 (d, *J* = 16.6 Hz, 2H), 3.63 (t, *J* = 7.6 Hz, 2H), 2.85 (s, 3H), 2.06 (s, 3H), 1.73–1.60 (m, 2H), 1.46–1.29 (m, 2H), 0.65–0.56 (m, 2H). ^13^C NMR (100 MHz, CDCl_3_) δ 167.97, 134.66, 129.35, 129.31, 129.09, 128.88, 76.33, 62.02, 45.88, 44.87, 29.71, 21.19, 20.65, 14.32. ^11^B (128 MHz, CDCl_3_) δ 13.4. HRMS (ASAP) *m*/*z* calcd for C_18_H_26_BN_2_O_6_ (M+H)^+^: 377.1878, found 377.1890.

#### 5-(*N*-Benzyloxy cetamido)­pentyl Boronic Aacid MIDA Ester (**15c**)

MIDA Ester **13c** (3.00 g, 6.69 mmol), DCM (30 mL), and TFA (5.2 mL, 7.74 g, 67.8 mmol) were stirred for 2 h. Intermediate amine (2.33 g, 6.69 mmol), TEA (1.02 mL, 740 mg, 7.31 mmol), Ac_2_O (0.95 mL, 1.02 g, 9.99 mmol), and DCM (30 mL) were stirred, worked up, and dried under high vacuum to afford **15c** (1.65 g, 76% over two steps) as a hygroscopic, white solid. ^1^H NMR (400 MHz, CDCl_3_) δ 7.43–7.32 (m, 5H), 4.80 (s, 2H), 3.95 (d, *J* = 16.7 Hz, 2H), 3.70 (d, *J* = 16.7 Hz, 2H), 3.61 (t, *J* = 7.1 Hz, 2H), 2.87 (s, 3H), 2.06 (s, 3H), 1.69–1.58 (m, *J* = 7.1 Hz, 2H), 1.41–1.28 (m, 4H), 0.57 (t, *J* = 7.7 Hz, 2H). ^13^C NMR (100 MHz, CDCl_3_) δ 168.02, 134.66, 129.31, 129.07, 128.86, 76.32, 62.05, 45.91, 29.66, 26.72, 23.62, 20.67. ^11^B (128 MHz, CDCl_3_) δ 13.4. HRMS (ASAP) *m*/*z* calcd for C_19_H_28_BN_2_O_6_ (M+H)^+^: 391.2035, found 391.2050.

### General Procedure for MIDA Deprotection and Hydrogenation

In a round-bottom flask containing **14a**–**c** or 15 a–**c** (1 equiv) was added THF (10–22 mL) and 1 M NaOH (10–22 mL). The mixture was vigorously stirred for 15 min and then quenched with 3 M HCl (10–22 mL). The mixture was transferred to a separatory funnel and Et_2_O (20 mL) was added to extract. The aqueous layer was extracted further with Et_2_O (2 × 20 mL) and the combined organics were washed with brine, dried over Na_2_SO_4_, and concentrated. Drying afforded the intermediate boronic acid, which was used without further purification. Pd/C (10% on carbon, 10 mol %) was added to a round-bottom flask and MeOH (5–10 mL) was carefully added. A solution of the intermediate boronic acid dissolved in MeOH (5–25 mL) was added and the round-bottom flask was sealed with a septum. The atmosphere was exchanged with H_2_ gas via balloon three times via bubbling through the solution and then the reaction was allowed to stir under H_2_ (1 atm) for 3 h. Pd/C was then removed by filtration and the filtrate was concentrated. The residue was resuspended in ddH_2_O (∼5 mL), filtered through a 0.45 μm syringe filter, and the filter was washed with ddH_2_O (2 × 5 mL). The product was lyophilized to afford **1a–c** and **2a–c.**

#### 3-(*N*-Hydroxylformamido)­propyl Boronic Acid (**1a**)

MIDA boronate **14a** (876 mg, 2.51 mmol), 1 M NaOH (10 mL, 10 mmol), THF (10 mL), and 3 M HCl (10 mL, 30 mmol) were used for MIDA deprotection. The intermediate boronic acid (418 mg, 1.76 mmol) in MeOH (5 mL) was added to Pd/C (200 mg, 0.187 mmol) in MeOH (5 mL) for hydrogenation to afford **1a** (171 mg, 46%, over two steps) as a hygroscopic, light orange solid. NMR showed a mixture of rotational isomers. Purity by ^1^H NMR: 98.1%. ^1^H NMR (400 MHz, D_2_O) δ 8.25 + 7.90 (2*s*, 1H), 3.49 + 3.13 (2*m*, 2H), 1.83–1.63 (m, 2H), 0.83–0.52 (m, 2H). ^13^C NMR (100 MHz, D_2_O) δ 163.47, 159.38, 52.36, 48.29, 21.15, 20.54. ^11^B (128 MHz, D_2_O) δ 31.3. HRMS (ASAP) *m*/*z* calcd for C_4_H_9_BNO_3_ (M-H_2_O)^+^: 130.0670, found 130.0669.

#### 4-(*N*-Hydroxylformamido)­butyl Boronic Acid (**1b**)

MIDA boronate **14b** (1.74 mg, 4.99 mmol), 1 M NaOH (20 mL, 20 mmol), THF (20 mL), and 3 M HCl (20 mL, 60 mmol) were used for MIDA deprotection. The intermediate boronic acid (751 mg, 2.99 mmol) in MeOH (10 mL) was added to Pd/C (400 mg, 0.375 mmol) in MeOH (10 mL) for hydrogenation to afford **1b** (349 mg, 43% over two steps) as a hygroscopic, white solid. NMR showed a mixture of rotational isomers. Purity by ^1^H NMR: 97.6%. ^1^H NMR (400 MHz, D_2_O) δ 8.26 + 7.92 (2*s*, 1H), 3.61–3.48 (m, 2H), 1.69–1.56 (m, 2H), 1.46–1.31 (m, 2H), 0.79 (t, *J* = 7.9 Hz, 2H). ^13^C NMR (100 MHz, D_2_O) δ 159.40, 50.34, 46.45, 28.58, 28.09, 20.59, 20.20. ^11^B (128 MHz, D_2_O) δ 32.6. HRMS (ASAP) *m*/*z* calcd for C_5_H_11_BNO_3_ (M-H_2_O)^+^: 146.0827, found 146.0825.

#### 5-(*N*-Hydroxylformamido)­pentyl Boronic Acid (**1c**)

MIDA boronate **14c** (1.82 mg, 4.83 mmol), 1 M NaOH (20 mL, 20 mmol), THF (20 mL), and 3 M HCl (20 mL, 60 mmol) were used for MIDA deprotection. The intermediate boronic acid (1.11 g, 4.18 mmol) in MeOH (20 mL) was added to Pd/C (500 mg, 0.469 mmol) in MeOH (5 mL) for hydrogenation to afford **1c** (513 mg, 61%, over two steps) as a hygroscopic, off-white solid. NMR showed a mixture of rotational isomers. Purity by ^1^H NMR: 99.1%. ^1^H NMR (400 MHz, D_2_O) δ 8.26 + 7.92 (2*s*, 1H), 3.59–3.49 (m, 1H), 1.69–1.57 (m, 1H), 1.45–1.34 (m, 1H), 1.31–1.21 (m, 1H), 0.77 (t, *J* = 7.8 Hz, 1H). ^13^C NMR (100 MHz, D_2_O) δ 163.47, 159.37, 50.46, 46.56, 28.41, 28.00, 25.74, 25.26, 23.10, 23.01. ^11^B (128 MHz, D_2_O) δ 32.7. HRMS (ASAP) *m*/*z* calcd for C_6_H_13_BNO_3_ (M-H_2_O)^+^: 158.0983, found 158.0980.

#### 3-(*N*-Hydroxylacetamido)­propyl Boronic Acid (**2a**)

MIDA boronate **15a** (1.17 g, 3.23 mmol), 1 M NaOH (15 mL, 15 mmol), THF (15 mL), and 3 M HCl (13 mL, 39 mmol) were used for MIDA deprotection. The intermediate boronic acid (811 mg, 3.23 mmol) in MeOH (15 mL) was added to Pd/C (300 mg, 0.281 mmol) in MeOH (5 mL) for hydrogenation to afford **2a** (513 mg, 61%, over two steps) as a hygroscopic, light orange solid. Purity by ^1^H NMR: 99.0%. ^1^H NMR (400 MHz, D_2_O) δ 3.57 (t, *J* = 6.8 Hz, 2H), 2.10 (s, 3H), 1.80–1.61 (m, 2H), 0.79–0.66 (m, 2H). ^13^C NMR (100 MHz, D_2_O) δ 173.45, 49.51, 20.76, 19.14. ^11^B (128 MHz, D_2_O) δ 31.5. HRMS (ASAP) *m*/*z* calcd for C_5_H_11_BNO_3_ (M-H_2_O)^+^: 144.0827, found 144.0826.

#### 4-(*N*-Hydroxylacetamido)­butyl Boronic Acid (**2b**)

MIDA boronate **15b** (1.50 g, 3.98 mmol), 1 M NaOH (16 mL, 16 mmol), THF (16 mL), and 3 M HCl (16 mL, 48 mmol) were used for MIDA deprotection. The intermediate boronic acid (1.00 g, 3.77 mmol) in MeOH (20 mL) was added to Pd/C (400 mg, 0.375 mmol) in MeOH (5 mL) for hydrogenation to afford **2b** (277 mg, 40%, over two steps) as a hygroscopic, light orange solid. Purity by ^1^H NMR: 96.4%. ^1^H NMR (400 MHz, D_2_O) δ 3.84–3.53 (m, 2H), 2.12 (s, 3H), 1.73–1.54 (m, 2H), 1.47–1.29 (m, 2H), 0.86–0.74 (m, 2H). ^13^C NMR (100 MHz, D_2_O) δ 173.51, 51.48, 47.64, 29.11, 28.40, 20.61, 19.20. ^11^B (128 MHz, D_2_O) δ 32.7. HRMS (ASAP) *m*/*z* calcd for C_6_H_13_BNO_3_ (M-H_2_O)^+^: 158.0983, found 158.0980.

#### 5-(*N*-Hydroxylacetamido)­pentyl Boronic Acid (**2c**)

MIDA boronate **15c** (2.07 g, 5.30 mmol), 1 M NaOH (22 mL, 22 mmol), THF (16 mL), and 3 M HCl (22 mL, 48 mmol) were used for MIDA deprotection. The intermediate boronic acid (1.48 g, 5.30 mmol) in MeOH (20 mL) was added to Pd/C (500 mg, 0.469 mmol) in MeOH (5 mL) for hydrogenation to afford **2c** (185 mg, 18%, over two steps) as a hygroscopic, white solid. Purity by ^1^H NMR: 99.6%. ^1^H NMR (400 MHz, D_2_O) δ 3.58 (t, *J* = 7.0 Hz, 2H), 2.10 + 2.08 (2*s*, 1H), 1.69–1.53 (m, 2H), 1.42–1.32 (m, 2H), 1.32–1.18 (m, 2H), 0.80–0.71 (m, 2H). ^13^C NMR (100 MHz, D_2_O) δ 173.45, 51.57, 47.78, 28.49, 28.30, 26.26, 25.56, 23.18, 19.51, 19.19. ^11^B (128 MHz, D_2_O) δ 32.9. HRMS (ASAP) *m*/*z* calcd for C_7_H_15_BNO_3_ (M-H_2_O)^+^: 172.1140, found 172.1136.

### General Procedure for the Preparation of the Trifluoroborate Salts

In a round-bottom flask containing the MIDA boronate **15a** or **15b** (1 equiv), THF was added THF (6–14 mL) and 1 M NaOH (4 equiv). The mixture was vigorously stirred for 15 min and then quenched with 3 M HCl (12 equiv). The mixture was transferred to a separatory funnel and Et_2_O (25–50 mL) was added to extract. The aqueous layer was extracted further with Et_2_O (2 × 25–50 mL) and the combined organics were washed with brine, dried over Na_2_SO_4_, and concentrated. Drying afforded the intermediate boronic acid, which was used without further purification. The intermediate boronic acid was dissolved in MeOH (5–10 mL) and KHF_2_ (5 equiv) was added followed by ddH_2_O (2–4 mL). The mixture was stirred vigorously at rt for 8 h and the reaction mixture was concentrated to dryness. To the crude solid was added acetone and the mixture was filtered to remove insoluble salts. The salts were washed with acetone (2 × 10 mL) and the filtrate was concentrated to provide a solid. The solid was dissolved in a minimum amount of acetone and Et_2_O was added to promote precipitation. The precipitate was collected by filtration, washed with Et_2_O (3 × 5–10 mL), and dried on a high vacuum to provide **3a** and **3b**.

#### Potassium 3-(*N*-Benzyloxyacetamido)­propyl Trifluoroborate (**3a**)

**15a** (1.3 g, 3.58 mmol), THF (14 mL), 1 M NaOH (14 mL, 14 mmol), and 3 M HCl (14 mL, 42 mmol) were used for deprotection. The intermediate boronic acid (770 mg, 3.06 mmol) in MeOH (10 mL) was treated with KHF_2_ (1.47 g, 18.8 mmol) followed by ddH_2_O (4 mL). After reaction and workup, **3a** (512 mg, 46% over two steps) was recovered as a white, fluffy solid. Purity by ^1^H NMR: 99.4%. ^1^H NMR (400 MHz, Acetone-d_6_) δ 7.50–7.43 (m, 2H), 7.43–7.31 (m, 3H), 4.89 (s, 2H), 3.62–3.53 (m, 2H), 2.95–2.90 (m, 1H), 1.99 (s, 3H), 1.67–1.55 (m, 2H), 0.17–0.03 (m, 2H). ^13^C NMR (100 MHz, Acetone-d_6_) δ 171.51, 136.08, 129.95, 129.08, 129.04, 76.05, 48.90, 23.97, 20.71. ^11^B NMR (128 MHz, Acetone-d_6_) δ 5.0. HRMS (ESI) *m*/*z* calcd for C_12_H_16_BF_3_NO_2_ (M – K)^−^: 274.1232, found 274.1240.

#### Potassium 4-(*N*-Benzyloxyacetamido)­butyl Trifluoroborate (**3b**)

**15b** (550 mg, 1.46 mmol), THF (6 mL), 1 M NaOH (5.8 mL, 5.8 mmol), and 3 M HCl (5.8 mL, 17.4 mmol) were used for deprotection. The intermediate boronic acid (387 mg, 1.46 mmol) in MeOH (5 mL) was treated with KHF_2_ (711 mg, 9.10 mmol) followed by ddH_2_O (1.9 mL). After reaction and workup, **3b** (185 mg, 39% over two steps) was recovered as a white, fluffy solid. Purity by ^1^H NMR: 97.6%. ^1^H NMR (400 MHz, Acetone) δ 7.50–7.43 (m, 2H), 7.43–7.32 (m, 3H), 4.89 (s, 2H), 3.60 (t, *J* = 7.5 Hz, 2H), 2.01 (s, 3H), 1.64–1.53 (m, 2H), 1.31–1.21 (m, 2H), 0.23–0.12 (m, 2H). ^13^C NMR (100 MHz, Acetone-d_6_) δ 172.13, 136.31, 130.21, 129.37, 129.31, 76.50, 46.24, 31.02, 23.31, 23.29, 20.76. ^11^B NMR (128 MHz, Acetone-d_6_) δ 5.4. HRMS (ESI) *m*/*z* calcd for C_13_H_18_BF_3_NO_2_ (M – K)^−^: 288.1388, found 288.1393.

### General Procedure for Bromination of Bromo Xylenes

To a flame-dried round-bottom flask fitted with a condenser and charged with a stir bar was added *N*-bromosuccinimide (2 equiv). A slurry of the solid was formed by adding CHCl_3_ or CCl_4_ (250 mL) and the appropriate bromoxylene (1 equiv) was added. AIBN (0.08–0.09 mol %) was added and the mixture was heated to reflux for 5 h. When reflux was complete, the reaction was cooled to rt, filtered, and insoluble solids were washed with DCM (∼40 mL). The filtrate was transferred to a separatory funnel and washed with sat’d NaHSO_3_ and then brine. The organic phase was dried over Na_2_SO_4_, decanted, and the solvent was evaporated to produce an off-white solid. The crude solid was vigorously stirred with hexanes (500 mL) and a white precipitate slowly formed and the hexanes were decanted to afford **17a**–**c**.

#### 2-Bromo-1,3-Bis­(bromomethyl)­benzene (**17a**)

*N*-Bromosuccinimide (84.1 g, 472 mmol), 2-bromo-*m-*xylene (30 mL, 41.6 g, 225 mmol), CHCl_3_ (250 mL), and AIBN (32 mg, 0.195 mmol, 0.09%) were used and the crude solid was vigorously stirred with hexanes (500 mL) and a white precipitate slowly formed. The precipitate was collected by filtration to afford the **17a** (31.6 g, 41%) as a white solid. All spectra matched those in the literature.^44^

#### 2-Bromo-1,4-Bis­(bromomethyl)­benzene (**17b**)

*N*-Bromosuccinimide (81.0 g, 455 mmol), 2-bromo-*p*-xylene (30 mL, 40.2 g, 217 mmol), CHCl_3_ (250 mL), and AIBN (29 mg, 0.176 mmol, 0.08%) were used and the crude solid was stirred with hexanes (500 mL) and a white precipitate slowly formed. The precipitate was collected by filtration to afford **17b** (27.6 g, 38%) as a white solid. All spectra match those in the literature.^45^

#### 3-Bromo-1,2-Bis­(bromomethyl)­benzene (**17c**)

*N*-Bromosuccinimide (84.1 g, 472 mmol), 2-bromo-*o*-xylene (30 mL, 41.6 g, 225 mmol, CCl_4_ (250 mL), and AIBN (32 mg, 0.195 mmol, 0.09%) were used and the crude solid was stirred with hexanes (500 mL) and hexane was decanted. The product was dried to afford **17c** (75 g, quant) as an orange oil. The product was analytically pure and did not require further purification. All spectra matched those in the literature.^46^

### General Procedure for Substitution and Ester Hydrolysis

To a round-bottom flask was added **17a**–**b** (1 equiv) and ACN:Me_2_CO (2:1, 250 mL). The solution was stirred and anhydrous KOAc (5 equiv) was added. The slurry was brought to reflux for 5 h. Upon completion, the reaction was cooled to rt, filtered, and the filtrate was concentrated. The residue was reconstituted in EtOAc (100 mL) and dH_2_O (100 mL) and transferred to a separatory funnel. The aqueous phase was extracted with EtOAc (2 × 100 mL) and the combined organics were washed with brine. The organic phase was then dried over Na_2_SO_4_ and concentrated to afford the intermediate diacetate which was used without further purification. The intermediate diacetate was added to a round-bottom flask fitted with a reflux condenser and THF (110 mL) was added to dissolve. To the mixture was added 3 M NaOH solution (110 mL) and the biphasic mixture was heated to 50 °C for 5 h. The reaction was then cooled to rt, diluted with EtOAc (100 mL) and dH_2_O (100 mL), and transferred to a separatory funnel. The aqueous phase was extracted with EtOAc (2 × 100 mL) and the combined organic phases were washed with brine. The organic phase was dried over Na_2_SO_4_ and concentrated to afford **18a–c.**

#### 2-Bromo-1,3-Bis­(hydroxymethyl)­benzene (**18a**)

Dibromide **17a** (31.6 g, 92.1 mmol), KOAc (45.2 g, 460 mmol), ACN:Me_2_CO (2:1, 250 mL) were used for the substitution. The intermediate diacetate (19.6 g, 65.1 mmol), 3 M NaOH (110 mL, 330 mmol), and THF (110 mL) were stirred and worked up to afford **18a** (13.1 g, 66% over two steps) as a white solid which was used without further purification. ^1^H NMR (400 MHz, DMSO-*d*_6_) δ 7.47–7.34 (m, 3H), 5.42 (t, *J* = 5.6 Hz, 2H), 4.53 (d, *J* = 5.5 Hz, 4H). ^13^C NMR (100 MHz, DMSO-*d*_6_) δ 140.99, 127.10, 126.29, 120.39, 62.88. HRMS (ASAP) *m*/*z*: calcd for C_8_H_8_BrO (M-H_2_O)^+^ 198.9753 + 200.9733, found 198.9753 + 200.9731.

#### 2-Bromo-1,4-Bis­(hydroxymethyl)­benzene (**18b**)

Dibromide **17b** (27.6 g, 80.5 mmol), KOAc (40 g, 407 mmol), ACN:Me_2_CO (2:1, 250 mL) were used for the substitution. The intermediate diacetate (19.6 g, 65.1 mmol), 3 M NaOH (110 mL, 330 mmol), and THF (110 mL) were stirred and worked up to afford **18b** (13.1 g, 75% over two steps) as a white solid which was used without further purification. ^1^H NMR (400 MHz, DMSO-*d*_6_) δ 7.50 (d, *J* = 1.6 Hz, 1H), 7.47 (d, *J* = 7.8 Hz, 1H), 7.31 (dd, *J* = 7.8, 1.6 Hz, 1H), 5.40 (t, *J* = 5.6 Hz, 1H), 5.30 (t, *J* = 5.8 Hz, 1H), 4.49 (t, *J* = 6.0 Hz, 4H). ^13^C NMR (100 MHz, DMSO-*d*_6_) δ 143.39, 139.18, 129.79, 127.98, 125.56, 120.91, 62.54, 61.96. HRMS (ASAP) *m*/*z*: calcd for C_8_H_8_BrO (M-H_2_O)^+^ 198.9753 + 200.9733, found 198.9753 + 200.9731.

#### 3-Bromo-1,2-Bis­(hydroxymethyl)­benzene (**18c**)

Dibromide **17c** (71 g, 221 mmol), KOAc (104 g, 1.05 mol), and ACN:Me_2_CO (2:1, 600 mL) were used for the substitution. The intermediate diacetate (14 g, 46.5 mmol), 3 M NaOH (80 mL, 247 mmol), and THF (80 mL) were stirred and worked up to afford **18c** (8.6 g, 18% over two steps) as a beige solid which was used without further purification. ^1^H NMR (400 MHz, DMSO-*d*_6_) δ 7.56–7.35 (m, 2H), 7.20 (t, *J* = 7.7 Hz, 1H), 5.27 (t, *J* = 5.4 Hz, 1H), 4.99 (t, *J* = 5.3 Hz, 1H), 4.69 (d, *J* = 5.3 Hz, 2H), 4.64 (d, *J* = 5.1 Hz, 2H). ^13^C NMR (100 MHz, DMSO-*d*_6_) δ 144.16, 137.18, 131.03, 129.26, 126.76, 124.78, 60.76, 59.69. HRMS (ASAP) *m*/*z*: calcd for C_8_H_10_BrO_2_ (M+H)^+^ 216.9859 + 218.9838, found 216.9858 + 218.9837.

### General Procedure for THP Protection of Diols (**19a–c**)

To a round-bottom flask containing diol **18a**–**c** (1 equiv) was added anhydrous DCM (100 mL), and the solid was suspended by stirring. Then, p-toluenesulfonic acid (10 mol %) was added quickly followed by DHP (2.5 equiv) at rt. The mixture became homogeneous upon vigorous stirring, and the resulting solution was stirred overnight. The reaction was quenched with sat’d NaHCO_3_ (30 mL) and transferred to a separatory funnel. The aqueous layer was extracted with DCM (3 × 75 mL), and the combined organics were washed with water and brine. The organics were concentrated, and the residue was subjected to flash chromatography (SiO_2_, 6:1 hexanes/EtOAc) to afford **19a**–**c**.

#### 2,2’-[(2-Bromo-1,3-phenylene)­bis­(methyleneoxy)]­bis­[tetrahydro-2*H*-pyran] (**19a**)

Prepared from **18a** (9.0 g, 41.4 mmol) to provide **19a** (10.0 g, 67%) as a white solid. ^1^H NMR (400 MHz, CDCl_3_) δ 7.45 (d, *J* = 7.5 Hz, 2H), 7.32 (dd, *J* = 8.1, 7.0 Hz, 1H), 4.85 (d, *J* = 13.4 Hz, 2H), 4.78 (t, *J* = 3.5 Hz, 2H), 4.61 (d, *J* = 13.4 Hz, 2H), 3.92 (ddd, *J* = 11.4, 8.7, 3.2 Hz, 2H), 3.60–3.51 (m, 2H), 2.01–1.38 (m, 12H). ^13^C NMR (100 MHz, CDCl_3_) δ 138.26, 127.95, 127.24, 123.04, 98.52, 68.93, 62.28, 30.82, 30.65, 25.58, 19.45. HRMS (ASAP) *m*/*z*: calcd for C_18_H_26_BrO_4_ (M+H)^+^ 385.1009 + 387.0989, found 385.1007 + 387.0986.

#### 2,2’-[(2-Bromo-1,4-phenylene)­bis­(methyleneoxy)]­bis­[tetrahydro-2*H*-pyran] (**19b**)

Prepared from **18b** (8.25 g, 38.0 mmol) to provide **19b** (10.1 g, 68%) as a clear oil. ^1^H NMR (400 MHz, CDCl_3_) δ 7.56 (d, *J* = 1.6 Hz, 1H), 7.47 (d, *J* = 7.8 Hz, 1H), 7.29 (dd, *J* = 7.9, 1.7 Hz, 1H), 4.81 (d, *J* = 13.2 Hz, 1H), 4.76 (t, *J* = 3.6 Hz, 1H) 4.74 (d, *J* = 12.4 Hz, 1H), 4.69 (t, *J* = 3.5 Hz, 1H), 4.57 (d, *J* = 13.2 Hz, 1H), 4.46 (d, *J* = 12.3 Hz, 1H), 3.91 (m, 2H), 3.55 (m, 2H), 1.95–1.49 (m, 12H). ^13^C NMR (CDCl_3_, 100 MHz): δ 139.3, 136.9, 131.6, 129.0, 126.6, 98.3, 97.7, 62.17, 62.14, 30.5, 30.4, 25.44, 25.42, 19.3, 19.2. HRMS (ASAP) *m*/*z*: calcd for C_13_H_18_BrO_3_ (*M*-(THP)+H)^+^ 301.0434 + 303.0413, found 301.0430 + 303.0411.

#### 2,2’-[(3-Bromo-1,2-phenylene)­bis­(methyleneoxy)]­bis­[tetrahydro-2*H*-pyran] (**19c**)

Prepared from **18c** (8.80 g, 40.5 mmol) to provide **19c** (9.6 g, 62%) as a clear oil and a mixture of diastereomers. ^1^H NMR (400 MHz, CDCl_3_) δ 7.52 (dd, *J* = 8.0, 1.3 Hz, 1H), 7.42 (dt, *J* = 7.4, 1.9 Hz, 2H), 7.15 (t, *J* = 7.8 Hz, 1H), 7.11–6.95 (m, 2H), 5.24 (d, *J* = 15.3 Hz, 1H), 5.07–4.53 (m, 11H), 4.04–3.90 (m, 1H), 3.93–3.85 (m, 1H), 3.89–3.81 (m, 1H), 3.76 (dt, *J* = 9.7, 6.6 Hz, 1H), 3.62–3.46 (m, 3H), 3.40 (dt, *J* = 9.7, 6.5 Hz, 1H), 1.91–1.44 (m, 24H). ^13^C NMR (100 MHz, CDCl_3_) δ 141.35, 140.46, 137.86, 135.36 (d), 132.45 (d), 131.82, 129.57, 128.25 (t), 126.58, 122.44, 106.12, 98.97 (d), 98.65 (d), 98.12 (d), 94.71, 69.54, 68.88, 67.45, 66.70 (d), 65.85 (d), 62.99, 62.42, 62.27, 62.17, 33.15, 30.85, 30.64, 30.57, 29.58, 25.59, 25.54 (d), 21.60, 19.83, 19.76, 19.43, 19.30 (d). HRMS (ASAP) *m*/*z*: calcd for C_13_H_18_BrO_3_ (*M*-(THP)+H)^+^ 301.0434 + 303.0413, found 301.0443 + 303.0422.

### General Procedure for the Synthesis of Hydroxybenzoxaboroles (**20a–c**)

To a flame-dried round-bottom flask were added THP ether **19a**–**c** (1 equiv) and THF (50 mL). The flask was cooled to −78 °C, and *n*-BuLi solution (2.5 M in hexanes, 1.03 equiv) was added dropwise via syringe over 30 min. After the addition, the mixture was stirred at −78 °C for 30 min. B­(OiPr)_3_ (1.03 equiv) was added via syringe at −78 °C, and the reaction was warmed to rt to stir for 3 h. Then, the reaction was cooled to 0 °C, quenched with sat’d NH_4_Cl (40 mL), and diluted with water/EtOAc. The aqueous layer was extracted with EtOAc (3 × 50 mL), and the combined organics were washed with brine, dried over Na_2_SO_4_, and concentrated. The crude residue was dissolved in MeOH (100 mL), and p-TSA (50 mol %) was added. The mixture was stirred for 5 h at rt and then concentrated to an oil. The residue was quickly dissolved in EtOAc and extracted (3 × 50 mL) against water (∼40 mL). The combined organics were washed with brine and dried over Na_2_SO_4_. Solvent removal produced a crude solid, which was suspended in hexanes, filtered, and washed with Et_2_O (3 × 20 mL) to produce **20a**–**c**. The compounds were analytically pure and did not require further purification.

#### 7-Hydroxymethyl Benzoxaborole (**20a**)

Prepared from **19a** (9.0 g, 23.3 mmol) to produce **20a** (1.65 g, 43% over three steps) as a white solid.^1^H NMR (CD_3_OD, 400 MHz): δ 7.44 (t, *J* = 7.5 Hz, 1H), 7.40–7.20 (m, 2H), 7.85 (s, 4H). ^13^C NMR (CD_3_OD, 100 MHz): δ 155.33, 146.83, 132.31, 126.02, 121.02, 72.26, 63.50. ^11^B NMR (128 MHz, CD_3_OD): δ 31.9. HRMS (ASAP) *m*/*z*: calcd for C_8_H_10_BO_3_ (M+H)^+^ 165.0718, found 165.0718.

#### 6-Hydroxymethyl Benzoxaborole (**20b**)

Prepared from **19b** (8.73 g, 22.6 mmol) to produce **20b** (3.71 g, 62% over three steps) as a white solid. ^1^H NMR (CD_3_OD, 400 MHz): δ 7.38 (s, 1H), 7.40 (d, 1H, *J* = 7.8), 7.34 (d, 1H, *J* = 7.8), 4.95 (s, 2H), 4.52 (s, 2H). ^13^C NMR (100 MHz, CD_3_OD) δ 154.53, 141.61, 131.12, 129.77, 122.15, 72.21, 65.20. ^11^B NMR (128 MHz, CD_3_OD): δ 32.0. HRMS (ASAP) *m*/*z*: calcd for C_8_H_10_BO_3_ (M+H)^+^ 165.0718, found 165.0719.

#### 4-Hydroxymethyl Benzoxaborole (**20c**)

Prepared from **19c** (9.4 g, 24.3 mmol) to produce **20c** (1.60 g, 40% over three steps) as a white solid. ^1^H NMR (400 MHz, CD_3_OD) δ 7.57 (d, *J* = 7.4 Hz, 1H), 7.42 (d, *J* = 7.5 Hz, 1H), 7.32 (t, *J* = 7.4 Hz, 1H), 5.14 (s, 2H), 4.62 (s, 2H). ^13^C NMR (CD_3_OD, 100 MHz): δ 153.33, 135.88, 130.41, 130.33, 128.45, 71.51, 62.87. ^11^B NMR (128 MHz, CD_3_OD): δ 32.0. HRMS (ASAP) *m*/*z*: calcd for C_8_H_10_BO_3_ (M+H)^+^ 165.0718, found 165.0719.

### General Procedure for Transient Benzoxaborole Protection and Iodide Substitution

To a round-bottom flask charged with a stir bar was added **20a**–**c** (1 equiv), Na_2_SO_4_ (8 equiv), and Et_2_O:Me_2_CO (1:1, 20–50 mL). The slurry was stirred and 3-dimethylamino-1-propanol (1.05 equiv) was added via micropipette. The mixture was stirred overnight at rt and the insoluble solids were removed by vacuum filtration. The solids were washed with EtOAc (2 × 30 mL) and the filtrate was concentrated to afford the intermediate protected benzoxaborole as an oily solid. The intermediate complex was directly dissolved in anhydrous ACN (30–40 mL) in a round-bottom flask and NaI (3.3 equiv) was added. The flask was flushed with argon and cooled to 0 °C. TMSCl (3 equiv) was added dropwise via syringe over 5 min and the mixture was warmed to rt over 3 h. After this time, the reaction mixture was concentrated, resuspended in Et_2_O (50 mL) and dH_2_O (50 mL), and transferred to a separatory funnel. The aqueous phase was extracted with Et_2_O (3 × 50 mL) and the combined organics were washed with sat’d NaHSO_3_ and brine. The organics were dried over Na_2_SO_4_, concentrated, and the resulting solid was suspended in hexanes. The solid was collected by filtration and washed with hexanes (2 × 10 mL) to afford **21a–c.**

#### 7-Iodomethyl Benzoxaborole (**21a**)

Alcohol **20a** (1.50 g, 9.14 mmol) Na_2_SO_4_ (10.1 g, 71.1 mmol), Et_2_O:Me_2_CO (1:1, 50 mL), and 3-dimethylamino-1-propanol (1.15 mL, 1.00 g, 9.72 mmol) were used for the benzoxaborole protection. The intermediate complex was dissolved in ACN (40 mL). TMSCl (3.50 mL, 2.99 g, 27.5 mmol) and NaI (4.52 g, 30.1 mmol) were used to afford **21a** (2.07 g, 83% over two steps) as a white solid. ^1^H NMR (400 MHz, CDCl_3_) δ 7.35 (q, *J* = 8.0, 1H), 7.31 (d, *J* = 8.0) 7.16 (d, *J* = 8.0 Hz, 1H), 5.01 (s, 2H), 4.74 (s, 2H). ^13^C NMR (100 MHz, CDCl_3_) δ 154.43, 143.65, 131.56, 128.12, 120.61, 70.77, 4.51. ^11^B NMR (128 MHz, CDCl_3_): δ 31.9. HRMS (ASAP) *m*/*z*: calcd for C_8_H_9_BIO_2_ (M+H)^+^ 274.9735, found 274.9747.

#### 6-Iodomethyl Benzoxaborole (**21b**)

Alcohol **20b** (1.50 g, 9.14 mmol) Na_2_SO_4_ (10.2 g, 71.8 mmol), Et_2_O:Me_2_CO (1:1, 50 mL), and 3-dimethylamino-1-propanol (1.15 mL, 1.00 g, 9.72 mmol) were used for the benzoxaborole protection. The intermediate complex was dissolved in ACN (30 mL). TMSCl (3.50 mL, 2.99 g, 27.5 mmol) and NaI (4.51 g, 30.0 mmol) were used to afford **21b** (1.90 g, 76% over two steps) as an off-white solid. ^1^H NMR (400 MHz, CDCl_3_) δ 7.78 (s, 1H), 7.55 (bs, 1H), 7.46 (dd, *J* = 7.9, 1.8 Hz, 1H), 7.24 (d, *J* = 8.2 Hz, 1H), 5.01 (s, 2H), 4.50 (s, 2H). ^13^C NMR (100 MHz, CDCl_3_) δ 153.64, 138.30, 131.51, 130.88, 121.62, 70.96, 6.04. ^11^B NMR (128 MHz, CDCl_3_): δ 32.2. HRMS (ASAP) *m*/*z*: calcd for C_8_H_9_BIO_2_ (M+H)^+^ 274.9735, found 274.9746.

#### 4-Iodomethyl Benzoxaborole (**21c**)

Alcohol **20c** (830 mg, 5.06 mmol) Na_2_SO_4_ (5.7 g, 40.1 mmol), Et_2_O:Me_2_CO (1:1, 20 mL), and 3-dimethylamino-1-propanol (616 μL, 537 mg, 5.26 mmol) were used for the benzoxaborole protection. The intermediate complex was dissolved in ACN (30 mL). TMSCl (2.0 mL, 1.71 g, 15.7 mmol) and NaI (2.41 g, 16.0 mmol) were used to afford **21c** (1.25 g, 91% over two steps) as an off-white solid. ^1^H NMR (400 MHz, CDCl_3_) δ 7.70 (d, *J* = 7.3, 1H), 7.42 (dd, *J* = 7.6, 1.1 Hz, 1H), 7.31 (t, *J* = 7.4 Hz, 1H), 5.07 (s, 2H), 4.38 (s, 2H). ^13^C NMR (100 MHz, CDCl_3_) δ 152.23, 132.39, 131.14, 130.71, 128.15, 69.60, 1.58. ^11^B NMR (128 MHz, CDCl_3_): δ 32.5. HRMS (ASAP) *m*/*z*: calcd for C_8_H_9_BIO_2_ (M+H)^+^ 274.9735, found 274.9745.

#### *N*-[(Tetrahydro-2*H*-pyran-2-yl)­oxy] Acetamide

Synthesized from *O*-tetrahydropyranylhydroxylamine according to literature procedures to afford a white solid (4.1 g, 61%). All spectra matched those reported in the literature.^47^

#### *N*-[(Tetrahydro-2*H*-pyran-2-yl)­oxy] Benzamide

Synthesized from *O*-tetrahydropyranylhydroxylamine according to literature procedures to afford a white solid (2.63 g, 40%). All spectra matched those reported in the literature.^48^

### General Procedure for Substitution and THP Deprotection

Utilizing a glovebox, a dry round-bottom flask with a stir bar was charged with NaH (1.3 equiv) and sealed. After removal from the glovebox, anhydrous DMF (15–20 mL) was added and the mixture was cooled to 0 °C. With stirring, *N*-[(tetrahydro-2*H*-pyran-2-yl)­oxy] acetamide or *N*-[(tetrahydro-2*H*-pyran-2-yl)­oxy] benzamide (1.2 equiv) was added in portions over a few min and stirred at 0 °C for 30 min. **21a**–**c** (1 equiv) was then added at 0 °C and the reaction was stirred at rt overnight. The mixture was concentrated and the residue was resuspended in EtOAc (40 mL) and sat’d NH_4_Cl (40 mL) and transferred to a separatory funnel. The aqueous layer was extracted with EtOAc (3 × 40 mL) and combined organics were washed with brine. The organic layer was dried over Na_2_SO_4_ and concentrated to a crude oil that was directly dissolved in MeOH (15–20 mL) in a round-bottom flask. p-TSA (1 equiv) was added and the mixture was stirred for 5–10 h at rt. The mixture was concentrated and reconstituted in EtOAc (50 mL) and dH_2_O (50 mL) and transferred to a separatory funnel. The aqueous layer was extracted with EtOAc (2 × 50 mL) and combined organics were washed with brine, dried over Na_2_SO_4_, and concentrated. The solid was suspended in 1:1 hexane:Et_2_O (∼30 mL) and collected by vacuum filtration. Purification afforded benzoxaboroles **4**–**7**.

#### 7-[(*N*-Hydroxyacetamido)­methyl] Benzoxaborole (**4**)

Iodide **21a** (1.8 g, 6.57 mmol), NaH (212 mg, 8.83 mmol), *N*-[(tetrahydro-2*H*-pyran-2-yl)­oxy] acetamide (1.27 g, 7.97 mmol), and DMF (20 mL) were used for the substitution. The intermediate benzoxaborole was dissolved in MeOH (20 mL) and p-TSA (1.25 g, 6.57 mmol) was stirred for 7 h. The crude product was purified further by heating in Me_2_CO and collecting the insoluble solid by vacuum filtration to afford **4** (386 mg, 27% over two steps) as an off-white solid. Purity by ^1^H NMR: 95.4%. ^1^H NMR (400 MHz, CD_3_OD) δ 7.48 (t, *J* = 7.6 Hz, 1H), 7.32 (d, *J* = 7.7 Hz, 1H), 7.24 (d, *J* = 7.6 Hz, 1H), 5.11 (s, 2H), 5.01 (s, 2H), 2.19 (s, 3H). ^13^C NMR (100 MHz, CD_3_OD) δ 173.69, 155.38, 141.73, 132.35, 126.65, 121.16, 72.33, 51.03, 20.39. ^11^B NMR (128 MHz, CD_3_OD): δ 32.03. HRMS (ASAP) *m*/*z*: calcd for C_10_H_13_BNO_4_ (M+H)^+^ 221.0932, found 222.0928.

#### 6-[(*N*-Hydroxyacetamido)­methyl] Benzoxaborole (**5**)

Iodide **21b** (1.8 g, 6.57 mmol), NaH (216 mg, 9.00 mmol)), *N*-[(tetrahydro-2*H*-pyran-2-yl)­oxy] acetamide (1.26 g, 7.91 mmol), and DMF (20 mL) were used for the substitution. The intermediate benzoxaborole was dissolved in MeOH (20 mL) and p-TSA (1.26 g, 6.62 mmol) was stirred for 7 h. The product was further purified by flash chromatography (SiO_2_, 40:1 DCM:EtOH → 20:1 DCM:EtOH → 10:1 DCM:EtOH) and dried under high vacuum to afford **5** (301 mg, 21% over two steps) as an orange solid. Purity by ^1^H NMR: 97.2%. ^1^H NMR (400 MHz, CD_3_OD) δ 7.60 (s, 1H), 7.43 (dd, *J* = 7.9, 1.7 Hz, 1H), 7.36 (d, *J* = 7.9 Hz, 1H), 5.06 (s, 2H), 4.79 (s, 2H), 2.14 (s, 3H). ^13^C NMR (100 MHz, CD_3_OD) δ 173.65, 154.74, 136.67, 132.27, 131.20, 122.31, 72.20, 52.66, 20.23. ^11^B NMR (128 MHz, CD_3_OD): δ 31.9. HRMS (ASAP) *m*/*z*: calcd for C_10_H_13_BNO_4_ (M+H)^+^ 221.0932, found 222.0928.

#### 6-[(*N*-Hydroxybenzoylamido)­methyl] Benzoxaborole (**6**)

A dry round-bottom flask with a stir bar was pumped into a glovebox and NaH (100 mg, 4.17 mmol) was added. To Iodide **21b** (1.0 g, 3.65 mmol), NaH (100 mg, 4.17 mmol), *N*-[(tetrahydro-2*H*-pyran-2-yl)­oxy] benzamide (770 mg, 3.48 mmol), and DMF (15 mL) were used for the substitution. The intermediate benzoxaborole was dissolved in MeOH (15 mL) and p-TSA (660 mg, 3.47 mmol) was stirred for 5 h. The product was further purified with a C_18_–Phenomenex Sep-Pak using 7:3 dH_2_O:ACN as the eluent. The fractions containing product were lyophilized to afford **6** (57 mg, 5.7% over two steps) as a white solid. Purity by ^1^H NMR: 95.5%. ^1^H NMR (400 MHz, CD_3_OD) δ 7.82–7.57 (m, 3H), 7.57–7.30 (m, 5H), 5.06 (s, 2H), 4.93 (s, 2H). ^13^C NMR (100 MHz, CD_3_OD) δ 154.88, 136.49, 135.70, 132.75, 132.14, 131.58, 131.08, 129.77, 129.64, 129.26, 129.08, 128.08, 122.41, 72.22. ^11^B NMR (128 MHz, CD_3_OD): δ 31.1. HRMS (ASAP) *m*/*z*: calcd for C_15_H_14_BNO_4_ (M+H)^+^ 284.1089, found 284.1083.

#### 4-[(*N*-Hydroxyacetamido)­methyl] Benzoxaborole (**7**)

Iodide **21c** (1.6 g, 5.84 mmol), NaH (242 mg, 10.1 mmol), *N*-[(tetrahydro-2*H*-pyran-2-yl)­oxy] acetamide (1.43 g, 8.98 mmol), and DMF (15 mL) were used for the substitution. The intermediate benzoxaborole was dissolved in MeOH (20 mL) and p-TSA (1.15 g, 6.04 mmol) was stirred for 10 h. The product was further purified by heating in Me_2_CO and collecting the insoluble solid by vacuum filtration. Drying on a high vacuum afforded **7** (248 mg, 19% over two steps) as an off-white solid. Purity by ^1^H NMR: 98.6%. ^1^H NMR (400 MHz, CD_3_OD) δ 7.67–7.57 (m, 1H), 7.41 (dd, *J* = 7.5, 1.2 Hz, 1H), 7.35 (t, *J* = 7.3 Hz, 1H), 5.11 (s, 2H), 4.76 (s, 2H), 2.13 (s, 3H). ^13^C NMR (100 MHz, CD_3_OD) δ 172.23, 152.68, 131.57, 129.54, 129.42, 127.23, 70.26, 48.37, 18.79. ^11^B NMR (128 MHz, CD_3_OD): δ 32.0. HRMS (ASAP) *m*/*z*: calcd for C_10_H_13_BNO_4_ (M+H)^+^ 221.0932, found 222.0929.

#### 2-Bromo-5-(trifluoromethyl) Benzaldehyde Diethyl Acetal (Scheme 3, Step b)

To a round-bottom flask charged with **22b** (5.1 g, 20.2 mmol) was added EtOH (50 mL) and p-TSA (318 mg, 1.67 mmol). Triethyl orthoformate (8.0 mL, 7.1 g, 48 mmol) was added and the solution was brought to reflux for 6 h. The reaction mixture was concentrated and dissolved in EtOAc (50 mL). The solution was transferred to a separatory funnel and extracted against dH_2_O (50 mL). The aqueous layer was extracted further with EtOAc (2 × 50 mL) and the combined organics were washed with sat’d NaHSO_3_ and brine. The organics were dried over Na_2_SO_4_ and concentrated to afford the acetal intermediate (5.98 g, 91%) as a clear oil. The product was analytically pure and did not require further purification. ^1^H NMR (400 MHz, CDCl_3_) δ 7.92 (d, *J* = 2.4 Hz, 1H), 7.63 (d, *J* = 8.3 Hz, 1H), 7.43 (dd, *J* = 8.3, 2.4 Hz, 1H), 5.66 (s, 1H), 3.75–3.54 (m, 4H), 1.26 (t, *J* = 7.1 Hz, 6H). ^13^C NMR (100 MHz, CDCl_3_) δ 139.26, 133.54, 129.95 (d, *J* = 32.8 Hz), 126.85 (m), 126.61 (q, *J* = 3.6 Hz), 126.49 (q, *J* = 271 Hz), 125.45 (q, *J* = 3.7 Hz), 100.74, 62.83, 15.24. HRMS (ASAP) *m*/*z*: calcd for C_10_H_9_BrFO (M-EtOH)^+^ 280.9783 + 282.763, found 280.9781 + 22.9760.

#### Potassium 2-Formylphenyl Trifluoroborate (**23a**)

Modified from literature procedures.^49^ To a round-bottom flask was added 2-formylphenylboronic acid **22a** (5.0 g, 33.3 mmol) and Et_2_O (80 mL). KHF_2_ (13.0 g, 166 mmol) was added to the solution followed by dH_2_O (4.0 mL) in one portion. The mixture was stirred at rt overnight and the precipitate was dissolved in Me_2_CO. Insoluble solids were removed by vacuum filtration and the filtrate was concentrated. The crude solid was dissolved in a minimum amount of Me_2_CO and added to Et_2_O (500 mL) to promote precipitation. The solid was collected by vacuum filtration, washed with Et_2_O, and dried to afford **23a** (6.7 g, 95%) as a shiny, white solid. All spectra match those in the literature.^49^

#### Potassium 2-Formyl-5-(trifluoromethyl) Trifluoroborate (**23b**)

To a dry round-bottom flask under argon charged with 2-bromo-5-(trifluoromethyl) benzaldehyde diethyl acetal (3.14 g, 9.59 mmol) was added THF (30 mL) and the solution was cooled to −78 °C. *n*-BuLi solution (2.5 M in hexanes, 4.0 mL, 10.0 mmol) was added via syringe over 15 min at −78 °C and the solution was stirred at −78 °C for 30 min. After this time, B­(OiPr)_3_ (2.4 mL, 1.95 g, 10.4 mmol) was added via syringe at −78 °C and the mixture was warmed to rt for 1.5 h. The reaction was then quenched with 3 M HCl (30 mL) at rt and the mixture was stirred for 30 min. EtOAc (40 mL) was added and the mixture was extracted in a separatory funnel. The aqueous layer was further extracted with EtOAc (2 × 40 mL) and the combined organics were washed with brine. The organics were dried over Na_2_SO_4_ and concentrated to a residue that was dried under high vacuum. The crude residue was dissolved in Et_2_O (40 mL) and KHF_2_ (3.8 g, 48.6 mmol) was added. dH_2_O (1.5 mL) was added in one portion and the mixture was stirred for 1 h. The precipitate was dissolved in Me_2_CO and insoluble solids were removed by vacuum filtration. The filtrate was concentrated and the residue was dissolved in a minimum amount of Me_2_CO before promoting precipitation in Et_2_O (300 mL). The solid was collected by vacuum filtration, washed with Et_2_O (2 × 100 mL), and dried under high vacuum to afford **23b** (2.68 g, 75% over two steps) as a shiny, white solid. ^1^H NMR (400 MHz, Acetone-d_6_) δ 10.64 (s, 1H), 8.01 (s, 1H), 7.94 (d, *J* = 7.8 Hz, 1H), 7.67 (dd, *J* = 7.9, 1.9 Hz, 1H). ^13^C NMR (100 MHz, Acetone-d_6_) δ 196.74, 140.92, 135.10 (q, *J* = 3.5 Hz), 129.32 (d, *J* = 71.7 Hz), 128.52 (d, *J* = 31.8 Hz), 128.11, q, *J* = 3.8 Hz), 125.69 (q, *J* = 269 Hz), 121.98 (q, *J* = 3.3 Hz). ^11^B NMR (128 MHz, CD_3_OD): δ 2.9. HRMS (ESI) *m*/*z* calcd for C_8_H_4_BF_6_O (M-K)^−^: 241.0265, found 241.0259.

#### 1,3-Dihydro-1-hydroxy-2,1-benzoxaborole-3-acetic Acid (**24a**)

To a dry round-bottom flask under argon charged with diisopropyl amine (4.2 mL, 3.03 g, 29.9 mmol) was added THF (50 mL) and the solution was cooled to −78 °C. *n*-BuLi solution (2.5 M, 9.6 mL, 24 mmol) was added over 5 min and the resulting mixture was stirred for an additional 15 min. *t*-Butyl acetate (4.8 mL, 3.46 g, 29.8 mmol) was added via syringe over 10 min at −78 °C. The solution was stirred for 30 min at −78 °C and solid **23a** (3.0 g, 14.1 mmol) was added to the flask in one portion. The mixture was warmed to rt and stirred for 7 h and then quenched with 0.5 M HCl (70 mL) and SiO_2_ (4.3 g, 74.9 mmol). The mixture was stirred at rt for 30 min, filtered with EtOAc washes (2 × 25 mL), and the filtrate was transferred to a separatory funnel. The aqueous layer was further extracted with EtOAc (2 × 50 mL) and the combined organics were washed with brine. The organics were dried over Na_2_SO_4_ and concentrated to a crude oil that was directly dissolved in anhydrous DCM (50 mL). TFA (10.5 mL, 15.6 g, 137 mmol) was added at rt and the mixture was stirred for 1 h. The orange solution was evaporated and redissolved in EtOAc (50 mL) and 3 M HCl (20 mL). The aqueous layer was further extracted with EtOAc (2 × 50 mL) and the combined organics were washed with sat’d NaHSO_3_ and brine. The organics were dried over Na_2_SO_4_, concentrated, and the solid was suspended in 1:1 hexane:Et_2_O (50 mL). The product was collected by vacuum filtration and dried under high vacuum to afford **24a** (1.10 g, 41% over three steps) as a beige solid. ^1^H NMR (400 MHz, Acetone-d_6_) δ 7.73 (d, *J* = 7.3 Hz, 1H), 7.54–7.45 (m, 2H), 7.45–7.21 (m, 2H), 5.56 (dd, *J* = 8.8, 4.4 Hz, 1H), 2.93 (ddd, *J* = 15.7, 4.5, 1.3 Hz, 1H), 2.50 (dd, *J* = 15.7, 8.9 Hz, 1H). ^13^C NMR (100 MHz, Acetone-d_6_) δ 172.10, 139.31, 131.67, 131.25, 128.28, 122.18, 78.43, 42.29. ^11^B NMR (128 MHz, Acetone-d_6_): δ 32.0. HRMS (ASAP) *m*/*z*: calcd for C_18_H_16_B_2_O_7_ (Dimer + Na)^+^ 389.0974, found 389.0965.

#### 1,3-Dihydro-1-hydroxy-5-(trifluoromethyl)-2,1-benzoxaborole-3-acetic Acid (**24b**)

To a dry round-bottom flask under argon charged with diisopropyl amine (1.6 mL, 1.15 g, 11.4 mmol) was added THF (20 mL) and the solution was cooled to −78 °C. *n*-BuLi solution (2.5 M, 3.6 mL, 9.0 mmol) was added over 5 min and the resulting mixture was stirred for an additional 20 min. *t*-Butyl acetate (1.8 mL, 1.55 g, 13.4 mmol) was added via syringe over 10 min at −78 °C. The solution was stirred for 30 min at −78 °C and solid **23b** (1.5 g, 5.35 mmol) was added to the flask in one portion. The mixture was warmed to rt and stirred for 6 h and then quenched with 0.5 M HCl (20 mL) and SiO_2_ (1.60 g, 26.6 mmol). The mixture was stirred at rt for 30 min, filtered with EtOAc washes (2 × 25 mL), and the filtrate was transferred to a separatory funnel. The aqueous layer was further extracted with EtOAc (2 × 50 mL) and the combined organics were washed with brine. The organics were dried over Na_2_SO_4_ and concentrated to a crude oil that was directly dissolved in anhydrous DCM (20 mL). TFA (4.2 mL, 6.25 g, 54.8 mmol) was added at rt and the mixture was stirred for 1 h. The orange solution was evaporated and redissolved in warm DCM. The solution was cooled and layered with hexanes to form a precipitate which was collected by vacuum filtration. Drying under high vacuum to afford **24b** (350 mg, 25% over three steps) as a beige solid. ^1^H NMR (400 MHz, Acetone-d_6_) δ 10.86 (s, 1H), 7.93 (d, *J* = 7.6 Hz, 1H), 7.88 (s, 1H), 7.71 (d, *J* = 8.1 Hz, 1H), 5.66 (dd, *J* = 8.3, 4.6 Hz, 1H), 3.06 (dd, *J* = 16.0, 4.7 Hz, 1H), 2.64 (dd, *J* = 16.0, 8.3 Hz, 1H). ^13^C NMR (100 MHz, Acetone-d_6_) δ 171.73, 157.94, 145.38, 140.26, 132.78, 131.22 (q, *J* = 256 Hz), 125.13 (q, *J* = 3.9 Hz), 121.57, 119.31 (q, *J* = 4.0 Hz), 78.45, 41.56. ^11^B NMR (128 MHz, CD_3_OD): δ 31.5. HRMS (ASAP) *m*/*z*: calcd for C_10_H_9_BF_3_O_4_ (M+H)^+^ 261.0541, found 261.0532.

#### 1,3-Dihydro-1-hydroxy-2,1-benzoxaborole-3-acetyl Hydroxamic Acid (**8**)

To a round-bottom flask charged with a stir bar was added **24a** (1.01 g, 5.26 mmol), Na_2_SO_4_ (2.0 g, 14.0 mmol). To the flask was added Et_2_O (15 mL) and Me_2_CO (25 mL) followed by 3-dimethylamino-1-propanol (634 μL, 552 mg, 5.35 mmol) via micropipette. The mixture was stirred for 10 h at rt and a clumpy precipitate formed over time. The reaction was concentrated and DCM (30 mL) was added to the same pot. CDI (1.47 g, 9.06 mmol) was added in one portion at rt and the mixture was stirred for 15 min. *O*-Tetrahydropyranylhydroxylamine (1.09 g, 9.30 mmol) was then added to the mixture in one portion at rt and the mixture was stirred overnight. The mixture was quenched with 0.5 M HCl, transferred to a separatory funnel, and extracted with DCM (3 × 50 mL). The combined organics were washed with brine, dried over Na_2_SO_4_, and concentrated to an oil that was immediately dissolved in MeOH (30 mL). To the solution was added p-TSA (2.1 g, 11.0 mmol) and the mixture was stirred overnight. The reaction was concentrated, the residue was dissolved in EtOAc (50 mL) and dH_2_O (50 mL), and the aqueous layer was extracted further with EtOAc (2 × 50 mL). Combined organics were washed with brine, dried over Na_2_SO_4_, and concentrated to produce a solid that was further purified by crystallization from hexanes. The solid from two crops was collected by vacuum filtration and dried under high vacuum to afford **8** (293 mg, 27% over three steps) as an off-white solid. Purity by ^1^H NMR: 96.2%. ^1^H NMR (400 MHz, CD_3_OD) δ 7.64 (d, *J* = 7.3 Hz, 1H), 7.47 (td, *J* = 7.4, 1.3 Hz, 1H), 7.43–7.25 (m, 2H), 5.58 (dd, *J* = 8.8, 4.3 Hz, 1H), 2.96 (dd, *J* = 15.7, 4.3 Hz, 1H), 2.53 (dd, *J* = 15.7, 8.8 Hz, 1H). ^13^C NMR (100 MHz, CD_3_OD) δ 172.70, 156.96, 132.03, 131.33, 128.65, 122.18, 79.24, 52.19, 42.51. ^11^B NMR (128 MHz, CD_3_OD): δ 31.5. HRMS (ASAP) *m*/*z*: calcd for C_9_H_11_BNO_4_ (M-NHOH)^+^ 175.0561, found 175.0555.

#### 1,3-Dihydro-1-hydroxy-5-(trifluoromethyl)-2,1-benzoxaborole-3-acetyl Hydroxamic Acid (**9**)

To a round-bottom flask charged with a stir bar was added **24b** (816 mg, 3.13 mmol), Na_2_SO_4_ (3.5 g, 24.6 mmol), and Et_2_O:Me_2_CO (1:1, 10 mL). The slurry was stirred and 3-dimethylamino-1-propanol (390 μL, 340 mg, 3.29 mmol) was added via micropipette. The mixture was stirred for 5 h at rt and the insoluble solids were removed by vacuum filtration. The solids were washed with EtOAc (3 × 15 mL) and the filtrate was concentrated to afford the intermediate protected benzoxaborole as an oily solid. The residue was dissolved in DCM (15 mL) and CDI (843 mg, 5.19 mmol) was added in one portion at rt. The mixture was stirred for 1 h and then **24b** (603 mg, 5.14 mmol) was added in one portion at rt. The mixture was stirred overnight. The mixture was quenched with sat’d NH_4_Cl (15 mL) and the pH was adjusted to below 5 with 3 M HCl. The mixture was transferred to a separatory funnel and extracted with DCM (3 × 30 mL). The combined organics were washed with brine, dried over Na_2_SO_4_, and concentrated to afford an oil that was immediately dissolved in MeOH (15 mL). To the solution was added p-TSA (1.0 g, 5.26 mmol) and the mixture was stirred at rt overnight. The reaction was concentrated and the residue was dissolved in EtOAc (50 mL) and dH_2_O (50 mL). The aqueous layer was extracted further with EtOAc (2 × 50 mL) and combined organics were washed with brine, dried over Na_2_SO_4_, and concentrated. The crude solid was suspended in Et_2_O and collected by vacuum filtration. The product was further purified with a C_18_–Phenomenex Sep-Pak using 7:3 dH_2_O:ACN as the eluent. The fractions containing product were lyophilized to afford **9** (73 mg, 8.5% over three steps) as a fluffy, white solid. Purity by ^1^H NMR: 95.3%. ^1^H NMR (400 MHz, CD_3_OD) δ 7.83 (d, *J* = 7.7 Hz, 1H), 7.76 (s, 1H), 7.67 (d, *J* = 7.7 Hz, 1H), 5.68 (dd, *J* = 8.7, 4.7 Hz, 1H), 3.07 (dd, *J* = 15.9, 4.5 Hz, 0.5H) + 2.76 (dd, *J* = 14.4, 4.5 Hz, 0.5H), 2.73–2.57 (m, 0.5H) + 2.43–2.26 (m, 0.5H). ^13^C NMR (CD_3_OD, 100 MHz) δ 171.06, 156.15, 132.41 (d, *J* = 32.4 Hz), 130.64, 130.32 (q, *J* = 284 Hz), 124.16 (m), 118.02 (m), 77.90, 50.86. ^11^B NMR (128 MHz, CD_3_OD): δ 30.9. HRMS (ASAP) *m*/*z*: calcd for C_10_H_7_BF_3_O_3_ (M-NHOH)^+^ 243.0435, found 243.0426.

#### 1,3-Dihydro-1-hydroxy-2,1-benzoxaborole-3-acetaldehyde (**25**)

To a flame-dried two-neck round-bottom flask was added Mg turnings (1.5 g, 61.7 mmol) and anhydrous THF (30 mL). The flask was fitted with a reflux condenser and bromomethyl-1,3-dioxolane (4.8 mL, 7.74 g, 46.3 mmol) was added in one portion via syringe at rt. The reaction was stirred and intermittently heated/cooled to initiate the reaction. Once initiated, the reagent was stirred for 30 min and allowed to naturally cool to rt. In a separate flame-dried round-bottom flask, **23a** (5.0 g, 23.5 mmol) was dissolved in anhydrous THF (30 mL) and cooled to 0 °C. The freshly prepared Grignard reagent was added to **23a** via syringe at 0 °C over 10 min and allowed to stir for an additional 10 min. The reaction was warmed to rt for 8 h and then quenched with 1 M HCl (70 mL) and SiO_2_ (7.1 g, 118 mmol). The mixture was stirred for 30 min and filtered to remove insoluble solids with EtOAc washes (3 × 30 mL). The filtrate was transferred to a separatory funnel and extracted with EtOAc (2 × 75 mL). The combined organics were washed with sat’d NaHSO_3_ and brine, dried over Na_2_SO_4_, and concentrated to an oil. The crude intermediate dioxolane was dissolved in THF (80 mL) and 3 M HCl (125 mL) was added at rt. The mixture was stirred at rt for 8 h and then transferred to a separatory funnel to be extracted with EtOAc (2 × 100 mL). The combined organics were washed with brine, dried over Na_2_SO_4_, and concentrated to an oil. This process was repeated two additional times with THF (80 mL) and 3 M HCl (125 mL) for 1.5 h at rt. The product was purified by flash chromatography (SiO_2_, 1:1 Et_2_O:hexanes → 3:1 Et_2_O:hexanes) to afford **25** (960 mg, 23% over three steps) as a yellow solid. ^1^H NMR (400 MHz, CDCl_3_) δ 9.87 (s, 1H), 7.76 (d, *J* = 7.3 Hz, 1H), 7.51 (td, *J* = 7.5, 1.2 Hz, 1H), 7.43–7.37 (m, 1H), 7.32 (dd, *J* = 7.7, 0.9 Hz, 1H), 5.71 (dd, *J* = 8.5, 4.1 Hz, 1H), 2.98 (ddd, *J* = 17.0, 4.1, 1.6 Hz, 1H), 2.76 (ddd, *J* = 17.0, 8.5, 2.1 Hz, 1H). ^13^C NMR (100 MHz, CDCl_3_) δ 200.19, 131.64, 130.97, 128.09, 121.22, 76.65, 50.17. ^11^B NMR (128 MHz, CDCl_3_): δ 32.1. HRMS (ASAP) *m*/*z*: calcd for C_9_H_9_BO_3_ (M+H)^+^ 177.0718, found 177.0724.

#### 1,3-Dihydro-1-hydroxy-2,1-benzoxaborole-3-*N*-(benzyloxy)­ethylamine (**26**)

In a round-bottom flask, **25** (890 mg, 5.05 mmol) was dissolved in THF (20 mL) and O-benzylhydroxylamine hydrochloride (1.0 g, 6.26 mmol) was added. TEA (1.2 mL, 871 mg, 8.61 mmol) was added via syringe at rt and the mixture was stirred for 9.5 h. The reaction was then quenched with 1 M HCl (50 mL), diluted with EtOAc (75 mL), and transferred to a separatory funnel. The aqueous layer was further extracted with EtOAc (2 × 75 mL) and the combined organics were washed with brine, dried over Na_2_SO_4_, and concentrated. The intermediate oxime was then dissolved in MeOH (15 mL) and pyridine-borane complex (1.61 g, 17.3 mmol) in MeOH (5 mL) was added. The mixture was stirred and conc. HCl (2.8 mL, 33.6 mmol) was added via syringe over a few min. The mixture was stirred at rt for 24 h, quenched with sat’d NaHCO_3_, and transferred to a separatory funnel. The aqueous layer was extracted with EtOAc (3 × 50 mL) and the combined organics were dried over Na_2_SO_4_. The organics were concentrated and dried under high vacuum to afford **26** (1.08 g, 75% over two steps) as a white solid. ^1^H NMR (400 MHz, CDCl_3_) δ 7.75–7.65 (m, 1H), 7.46 (td, *J* = 7.5, 1.2 Hz, 1H), 7.37–7.26 (m, 7H), 5.27 (dd, *J* = 9.2, 3.2 Hz, 1H), 4.73 (s, 2H), 3.31–3.06 (m, 2H), 2.27 (dtd, *J* = 14.5, 7.2, 3.3 Hz, 1H), 1.74 (ddd, *J* = 16.0, 11.6, 6.5 Hz, 1H). ^13^C NMR (100 MHz, CDCl_3_) δ 156.87, 137.69, 136.57, 131.12, 130.66, 128.57, 128.55, 128.02, 127.52, 121.17, 80.29, 76.24, 48.75, 34.30. ^11^B NMR (128 MHz, CDCl_3_): δ 31.8. HRMS (ASAP) *m*/*z*: calcd for C_16_H_19_BNO_3_ (M+H)^+^ 284.1453, found 284.1470.

#### 3-[(*N*-Benzyloxyacetamido)­ethyl] Benzoxaborole (**10**)

To a round-bottom flask charged with a stir bar was added **26** (1.08 g, 3.81 mmol), Na_2_SO_4_ (2.63 g, 18.5 mmol), and Et_2_O:Me_2_CO (1:1, 20 mL). The slurry was stirred and 3-dimethylamino-1-propanol (465 μL, 405 mg, 3.93 mmol) was added via micropipette. The mixture was stirred for 8 h at rt and the insoluble solids were removed by vacuum filtration. The solids were washed with EtOAc (3 × 15 mL) and the filtrate was concentrated to afford the intermediate protected benzoxaborole as an oily solid. The intermediate complex was dissolved in anhydrous DCM (20 mL) in a round-bottom flask and TEA (2.1 mL, 1.52 g, 15.0 mmol) and Ac_2_O (1.4 mL, 1.51 g, 14.8 mmol) were added at rt. The mixture was stirred overnight and then quenched with 1 M HCl (20 mL). The mixture was transferred to a separatory funnel and the aqueous layer was extracted with DCM (3 × 75 mL). The combined organics were washed with brine, dried over Na_2_SO_4_, and concentrated. The residue was dissolved in MeOH (10 mL) and added to a flask charged with a slurry of Pd/C (10% on carbon, 364 mg, 0.34 mmol) in MeOH (10 mL). The round-bottom flask was sealed with a septum and the atmosphere was exchanged with H_2_ gas via balloon three times via bubbling through the solution and then the reaction was allowed to stir under H_2_ (1 atm) overnight. Pd/C was then removed by filtration and the filtrate was concentrated. The product was purified with a C_18_–Phenomenex Sep-Pak using 7:3 dH_2_O:ACN as the eluent. The fractions containing product were lyophilized to afford **10** (73 mg, 8.5% over three steps) as a fluffy, white powder. Purity by ^1^H NMR: 86.5%. ^1^H NMR (400 MHz, CD_3_OD) δ 7.64 (d, *J* = 7.3 Hz, 1H), 7.47 (td, *J* = 7.5, 1.2 Hz, 1H), 7.39 (dd, *J* = 7.6, 1.0 Hz, 1H), 7.33 (t, *J* = 7.3 Hz, 1H), 5.25 (dd, *J* = 8.5, 3.4 Hz, 1H), 3.86–3.68 (m, 2H), 2.39–2.26 (m, 1H), 1.95–1.76 (m, 1H). ^13^C NMR (100 MHz, CD_3_OD) δ 173.74, 157.82, 132.47, 131.96, 131.29, 128.43, 122.22, 80.90, 45.60, 34.57, 20.19. ^11^B NMR (128 MHz, CD_3_OD): δ 31.4. HRMS (ASAP) *m*/*z*: calcd for C_11_H_15_BNO_4_ (M+H)^+^ 236.1089, found 236.1080.

### Biological Evaluation Methods

#### Enzyme Inhibition Assay Against *Ec*IspC^27,28^

Inhibition of *Ec*IspC was determined spectrophotometrically at 37 °C in a 1 mL cuvette. Compound (100 μM) was preincubated with purified *Ec*IspC for 1 min at 37 °C in a solution containing HEPES (50 mM, pH 8.0), MgCl_2_ (2 mM), NADPH (100 μM), TCEP (500 μM), and BSA (1 mg/mL). The reaction was initiated by the addition of DXP (100 μM) and residual enzyme activity was measured at 340 nm for 1 min at 37 °C. Reaction rates were determined via linear fitting and percent inhibition for each compound was calculated. Any compounds exhibiting >80% inhibition were selected to determine their IC_50_. The IC_50_ value of Fos was determined under the same conditions with a 1 min preincubation time with varied concentrations of Fos (5–160 nM). Experiments were run in triplicate. Data were plotted and fitted using Origin 2020 64-Bit.

#### *E. Coli* Growth Inhibition Screen^43^

*E. coli* WT was grown overnight in LB media at 37 °C and then diluted to an initial OD_600_ of 0.05. Compounds (100 μg/mL final concentration) were added to a 96-well plate along with the following controls: a sterility check (10% DMSO, LB media), negative control (10% DMSO), positive control (25 and 50 μg/mL of kanamycin in 10% DMSO), positive control (25 and 50 μg/mL of ampicillin in 10% DMSO). The final DMSO concentration in all wells was 1% v/v. Each compound concentration (15 μL) was deposited into their respective wells and inoculum (135 μL) was added to make a final volume of 150 μL and the plates were incubated at 37 °C for 16 h with shaking (300 rpm). The plates were read at 600 nm to determine OD_600_ and percent growth inhibition was calculated for each compound. Experiments were run in triplicate. Compounds exhibiting >80% inhibition at that concentration were selected to determine their MIC against *E. coli* WT and *E. coli* ΔGlpT.

#### *E. Coli* Growth Inhibition Assays^43^

*E. coli* WT and *E. coli* ΔGlpT were grown overnight in LB media at 37 °C and then diluted to an initial OD_600_ of 0.05. A concentration gradient of compound (1.56–200 μg/mL final concentration) or Fos (0.04–25 μg/mL final concentration) was added to a 96-well plate along with the following controls: a sterility check (10% DMSO, LB media), negative control (10% DMSO), positive control (25 and 50 μg/mL of kanamycin in 10% DMSO), positive control (25 and 50 μg/mL of ampicillin in 10% DMSO). The final DMSO concentration in all wells was 1% v/v. Fos was used as a negative control for *E. coli* ΔGlpT at 400 μg/mL. Each compound concentration (15 μL) was deposited into their respective wells and inoculum (135 μL) was added to make a final volume of 150 μL and the plates were incubated at 37 °C for 16 h with shaking (300 rpm). The plates were read at 600 nm to determine OD_600_ and the data was plotted and fitted to a Dose–response curve using Origin 2020 64-Bit. MICs are reported as the drug concentration that provides 80% inhibition of cell growth in this assay. Experiments were run in triplicate.

#### IPP Rescue Assay with *E. coli* WT^33^

*E. coli* WT was grown overnight in LB media at 37 °C and then diluted to an initial OD_600_ of 0.05. A concentration gradient of compound (1.56–200 μg/mL final concentration) or Fos (0.04–25 μg/mL final concentration) was added to a 96-well plate along with the following controls: a sterility check (10% DMSO, LB media), negative control (10% DMSO), positive control (25 and 50 μg/mL of kanamycin), positive control (25 and 50 μg/mL of ampicillin). Fos was used as a negative control for *E. coli* ΔGlpT at 400 μg/mL. The final DMSO concentration in all wells was 1% v/v. Each compound concentration (15 μL) was deposited into their respective wells followed by IPP (125 μM final concentration). Inoculum (135 μL) was added to make a final volume of 150 μL and the plates were incubated at 37 °C for 16 h with shaking (300 rpm). The plates were read at 600 nm to determine OD_600_ and the data was plotted and fitted using Origin 2020 64-Bit. Experiments were run in triplicate.

#### *S. aureus* and MRSA Growth Inhibition Assays^43^

*S. aureus* and MRSA were grown overnight in cation-adjusted Mueller Hinton broth (MH broth) at 37 °C and then diluted to an initial OD_600_ of 0.05. A concentration gradient of compound (1.56–200 μg/mL final concentration) was added to a 96-well plate along with the following controls: a sterility check (10% DMSO, MH broth), negative control (10% DMSO), positive control (50 μg/mL of levofloxacin). The final DMSO concentration in all wells was 1% v/v. Inoculum (135 μL) was added to make a final volume of 150 μL and the plates were incubated at 37 °C for 16 h with shaking (250 rpm). The plates were read at 600 nm to determine OD_600_ and the data was plotted and fitted using Origin 2020 64-Bit. Experiments were run in triplicate.

## Supplementary Material



## Notes

Includes final compound purity determinations via qNMR; NMR spectra for final compounds; EcIspC purification and kinetic characterization; MIC data for tested compounds; CO-ADD compound evaluation data (PDF)

## §

Ridgeline Discovery GmbH, Hochbergerstrasse 60F, 4057, Basel, Switzerland

## ∥

Neumann University, Science Department, One Neumann Drive, Aston, PA, 19014, United States.

## ⊥

King Saud bin Abdulaziz University for Health Sciences College of Nursing, Basic Sciences Department, Al Ahsa, Eastern Province, Saudi Arabia, 31982.

## #

University of Delaware, Department of Chemistry and Biochemistry, 210 S. College Ave, Newark, DE, 19716, United States.

## ¶

Washington University in St. Louis, Department of Biomedical Engineering, 1 Brookings Drive, St. Louis, MO, 63130, United States.

## Abbreviations

ACN: acetonitrile
Bn: benzyl
CDCl_3_: chloroform-d
CD_3_OD: methanol-d_4_
CHCl_3_: chloroform
DCM: dichloromethane
DMAPP: dimethylallyl pyrophosphate
DMF: dimethylformamide
DMSO: dimethyl sulfoxide
Et_2_O: diethyl ether
EtOAc: ethyl acetate
EtOH: ethanol
Fos: fosmidomycin
IPP: isopentenyl pyrophosphate
IspC: 1-deoxy-D-xylyulose-5-phosphate reductoisomerase
KOAc: potassium acetate
Me_2_CO: acetone
MeOH: methanol
MEP: 2-*C*-methyl-D-erythritol 4-phosphate
MIC: minimum inhibitory concentration
MRSA: methicillin-resistant *Staphylococcus aureus*
NADPH: nicotinamide adenine dinucleotide phosphate
Na_2_SO_4_: sodium sulfate
NH_4_Cl: ammonium chloride
SEM: standard error of the mean
THF: tetrahydrofuran
THP: tetrahydropyranyl
TLC: thin layer chromatography
WT: wild type
