# Supplementary material for: Boron-Containing Analogs of Fosmidomycin: Benzoxaborole Derivatives Exhibit Promising Activity Against Resistant Pathogens
Source: ACS Omega. 2025 Jul 19;10(29):31722–40. doi: 10.1021/acsomega.5c02701 (PMC12311745; doi:10.1021/acsomega.5c02701)
Supplement: Supplementary file 1 [file ao5c02701_si_001.pdf]

Boron-containing Analogs of Fosmidomycin: Benzoxaborole Derivatives Exhibit Promising Activity Against Resistant Pathogens

James M. Gamrat[a]; Christopher L. Orme[a]; Giulia Mancini[a†]; Sarah J. Burke[a‡]; Latifah M. Alhthlol[a§]; Rebecca C. Colandrea[a**]; Bryan C. Figula[a]; Dylan T. Tomares[a††]; Jason E. Heindl[b]; John W. Tomsho[a*]

[a] Saint Joseph's University, University City Campus, Department of Chemistry & Biochemistry, 600 S. 43rd St, Philadelphia, PA, 19104, United States.

[b] Rowan University, Department of Biological & Biomedical Sciences, 201 Mullica Hill Rd, Glassboro, NJ, 08028 United States.

[*] Corresponding Author: jtomsho@sju.edu

[†] GM is currently located at Ridgeline Discovery GmbH, Hochbergerstrasse 60F, 4057, Basel, Switzerland.

[‡] SJB is currently located at Neumann University, Science Department, One Neumann Drive, Aston, PA, 19014, United States.

[§] LMA is currently located at King Saud bin Abdulaziz University for Health Sciences College of Nursing, Basic Sciences Department, Al Ahsa, Eastern Province, Saudi Arabia, 31982.

[**] RCC is currently located at University of Delaware, Department of Chemistry & Biochemistry, 210 S. College Ave, Newark, DE, 19716, United States.

[††] DTT is currently located at Washington University in St. Louis, Department of Biomedical Engineering, 1 Brookings Drive, St. Louis, MO, 63130, United States.

Supporting Information

Table of Contents

Purity of Compounds by ^{1}H NMR S3
NMR Spectra of Compound Library S18
Purification and Kinetic Characterization of *Ec*IspC S64
Screening and microdilution assays of compound library against *E. coli* WT and *E. coli* ΔGlpT S66
*Ec*IspC Inhibition Assay Results S66
Minimum inhibitory concentration curves of active compounds S67
Data Tables from CO-ADD S75

Purity of compounds by ^{1}H NMR

Figure S1. ^{1}H NMR purity spectrum of **1a** with Me_2SO_2 in D_2O.

$$P = \frac{n\ i.c.*Int\ s*MW\ s*Mass\ i.c.}{n\ s.*Int\ i.c.*MW\ i.c*Mass\ s}\ x\ P\ i.c.$$

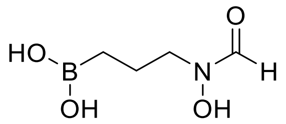

1a

$n_{i.c.}$: 6
$Int_{i.c.}$: 6
$MW_{i.c.}$: 94.13 g/mol
$Mass_{i.c.}$: 8.5 mg
$P_{i.c.}$: 99.4%

$n_{s.}$: 2
$Int_{s.}$: 1.22
MW_s: 146.94 g/mol
$Mass_{s.}$: 8.2 mg

P = 98.1%

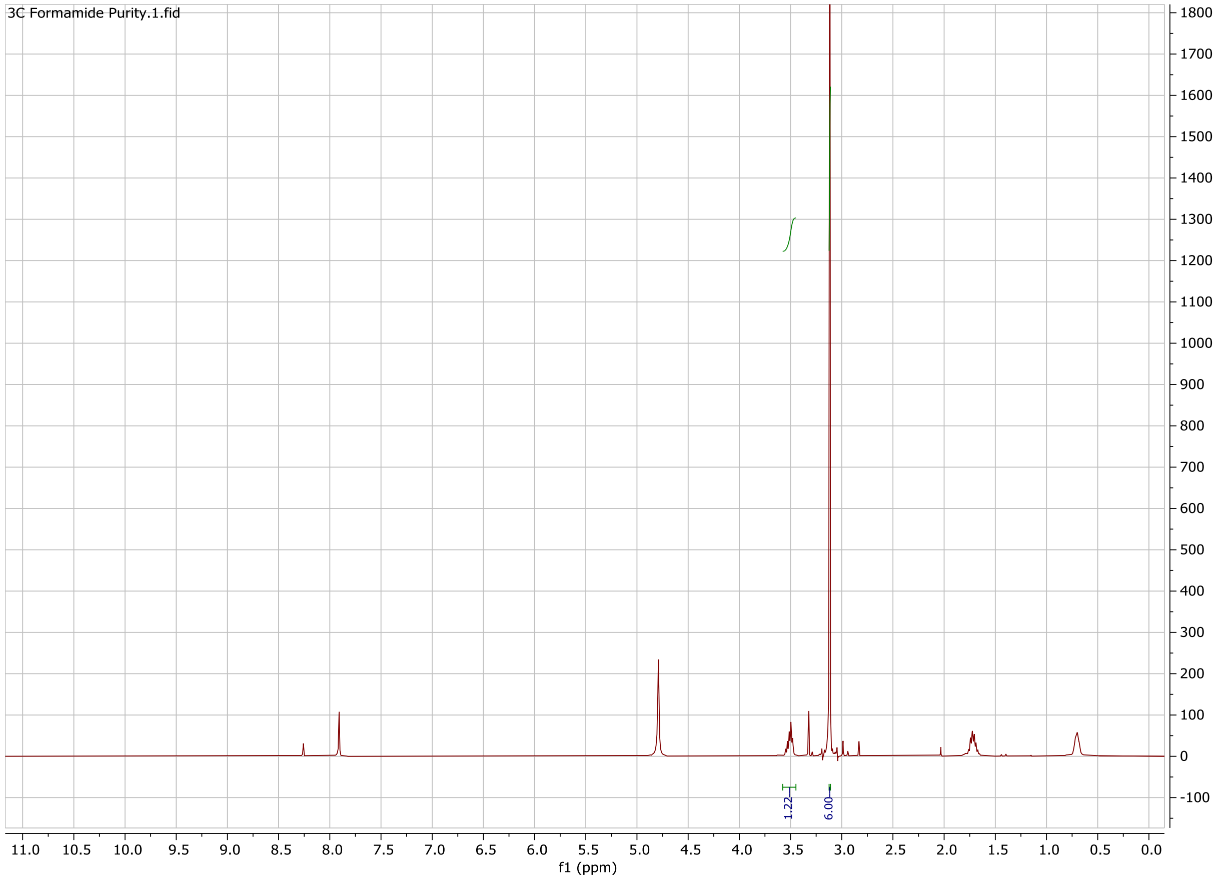

Figure S2. ^{1}H NMR purity spectrum of **1b** with Me_2SO_2 in D_2O.

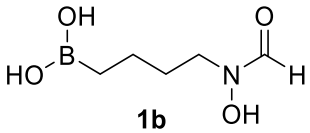

1b

P = 97.6%

$n_{i.c.}$: 6
$Int_{i.c.}$: 6
$MW_{i.c.}$: 94.13 g/mol
$Mass_{i.c.}$: 13.6 mg
$P_{i.c.}$: 99.4%

$n_{s.}$: 2
$Int_{s.}$: 1.81
MW_{s}: 160.96 g/mol
$Mass_{s.}$: 8.7 mg

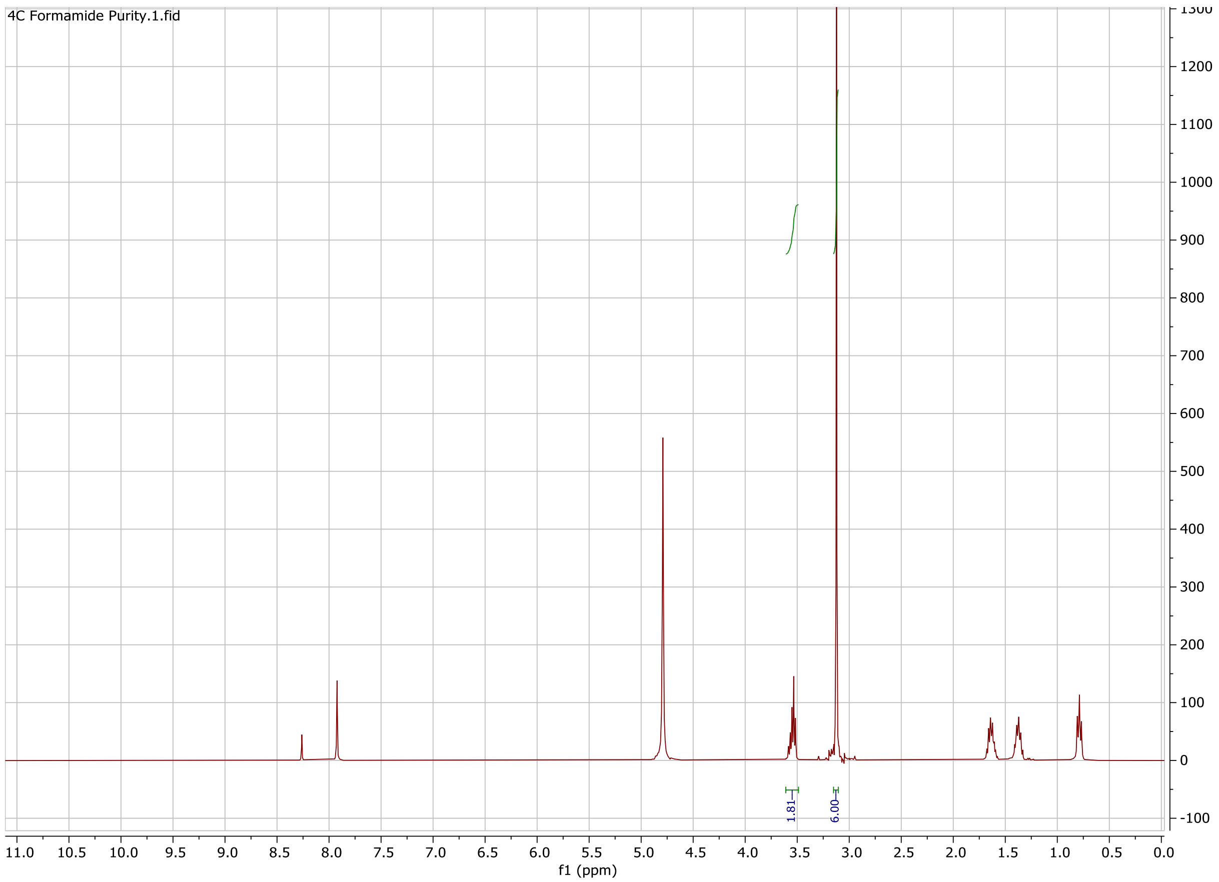

Figure S3. ^{1}H NMR purity spectrum of **1c** with Me_2SO_2 in D_2O.

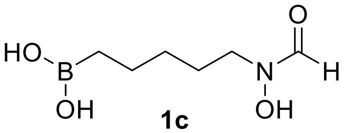

1c

P = 99.1%

$n_{i.c.}$: 6
$Int_{i.c.}$: 6
$MW_{i.c.}$: 94.13 g/mol
$Mass_{i.c.}$: 6.3 mg
$P_{i.c.}$: 99.4%

$n_{s.}$: 2
$Int_{s.}$: 1.26
MW_{s}: 174.94 g/mol
$Mass_{s.}$: 7.4 mg

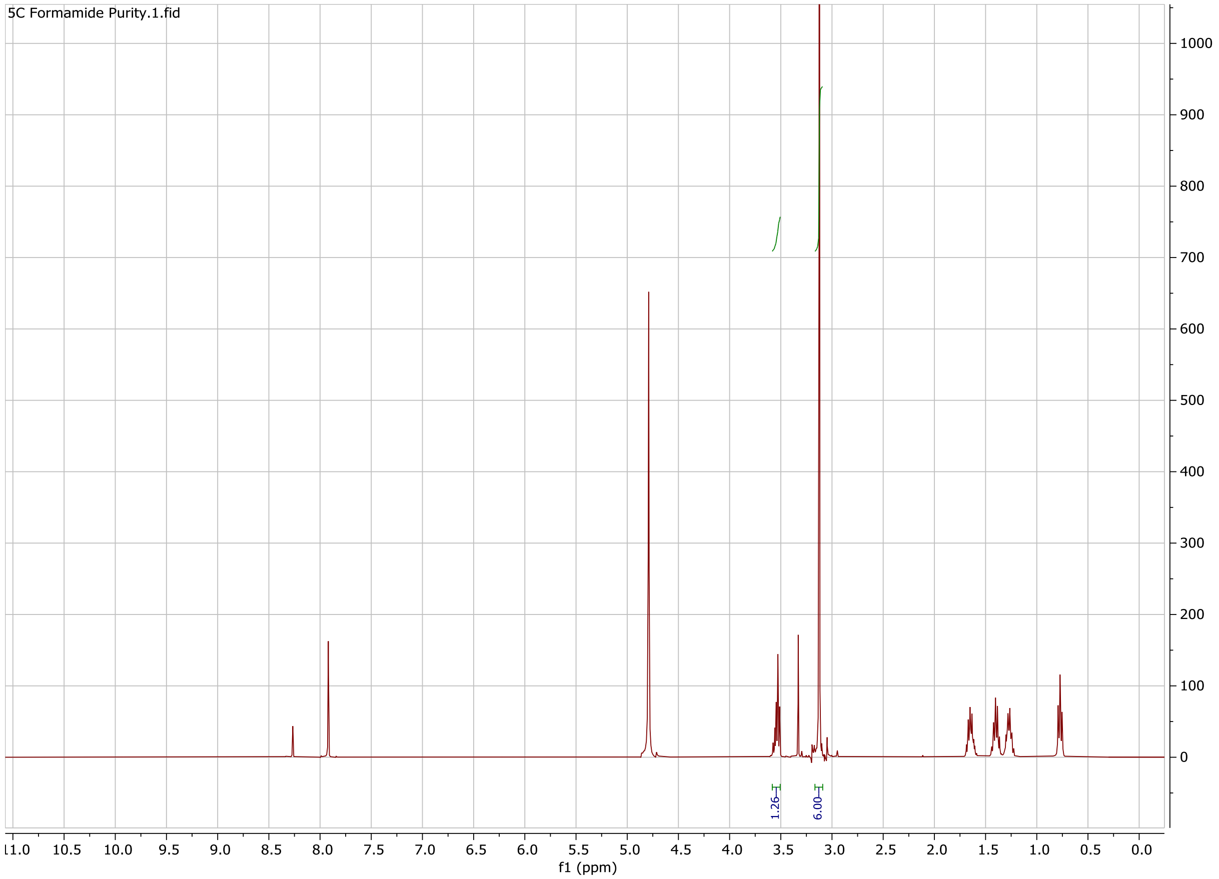

Figure S4. ^{1}H NMR purity spectrum of **2a** with Me_2SO_2 in D_2O.

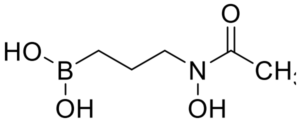

2a

P = 99.0%

$n_{i.c.}$: 6
$Int_{i.c.}$: 6
$MW_{i.c.}$: 94.13 g/mol
$Mass_{i.c.}$: 13.6 mg
$P_{i.c.}$: 99.4%

$n_{s.}$: 2
$Int_{s.}$: 0.66
MW_{s}: 160.96 g/mol
$Mass_{s.}$: 8.7 mg

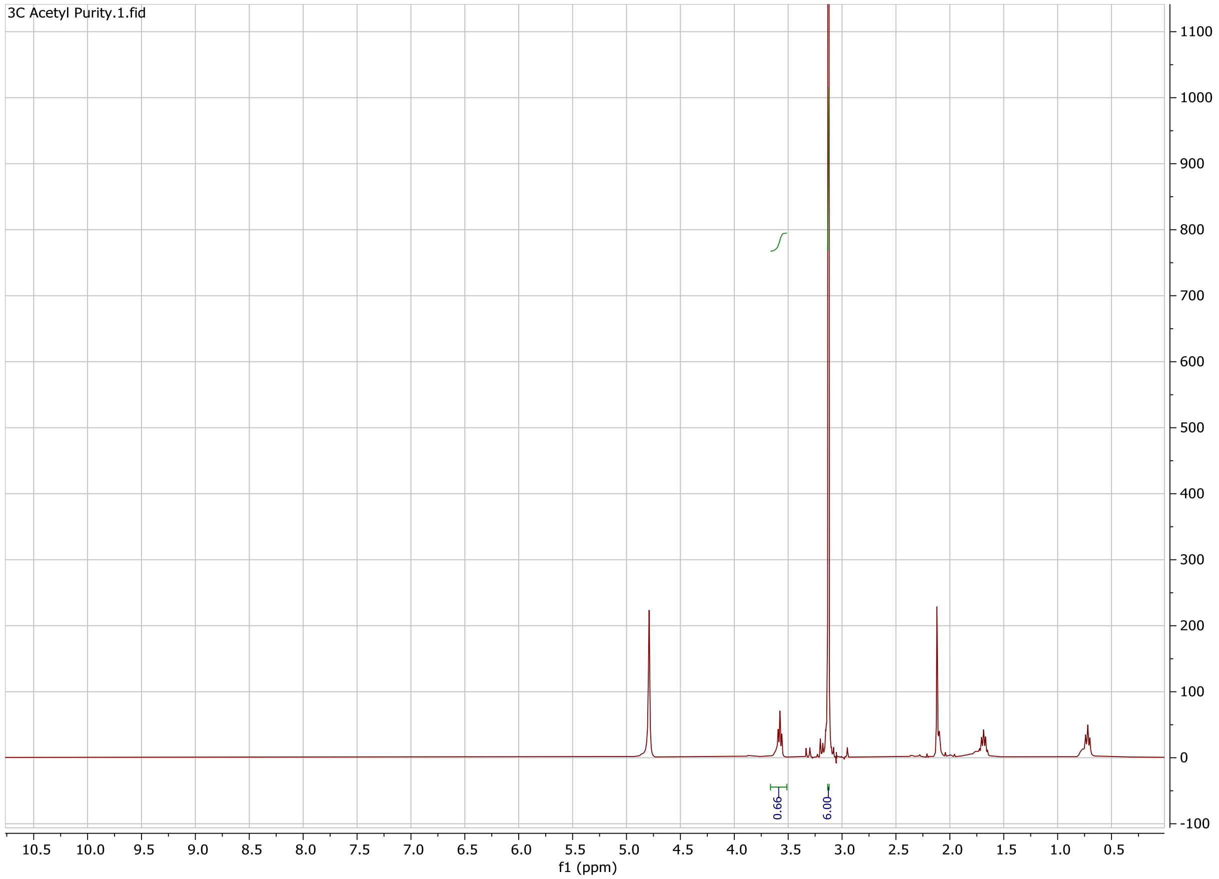

Figure S5. ^{1}H NMR purity spectrum of **2b** with Me_2SO_2 in D_2O.

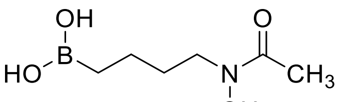

2b

P = 96.4%

$n_{i.c.}$: 6
$Int_{i.c.}$: 6
$MW_{i.c.}$: 94.13 g/mol
$Mass_{i.c.}$: 9.3 mg
$P_{i.c.}$: 99.4%

$n_{s.}$: 2
$Int_{s.}$: 1.01
MW_{s}: 174.99 g/mol
$Mass_{s.}$: 9.0 mg

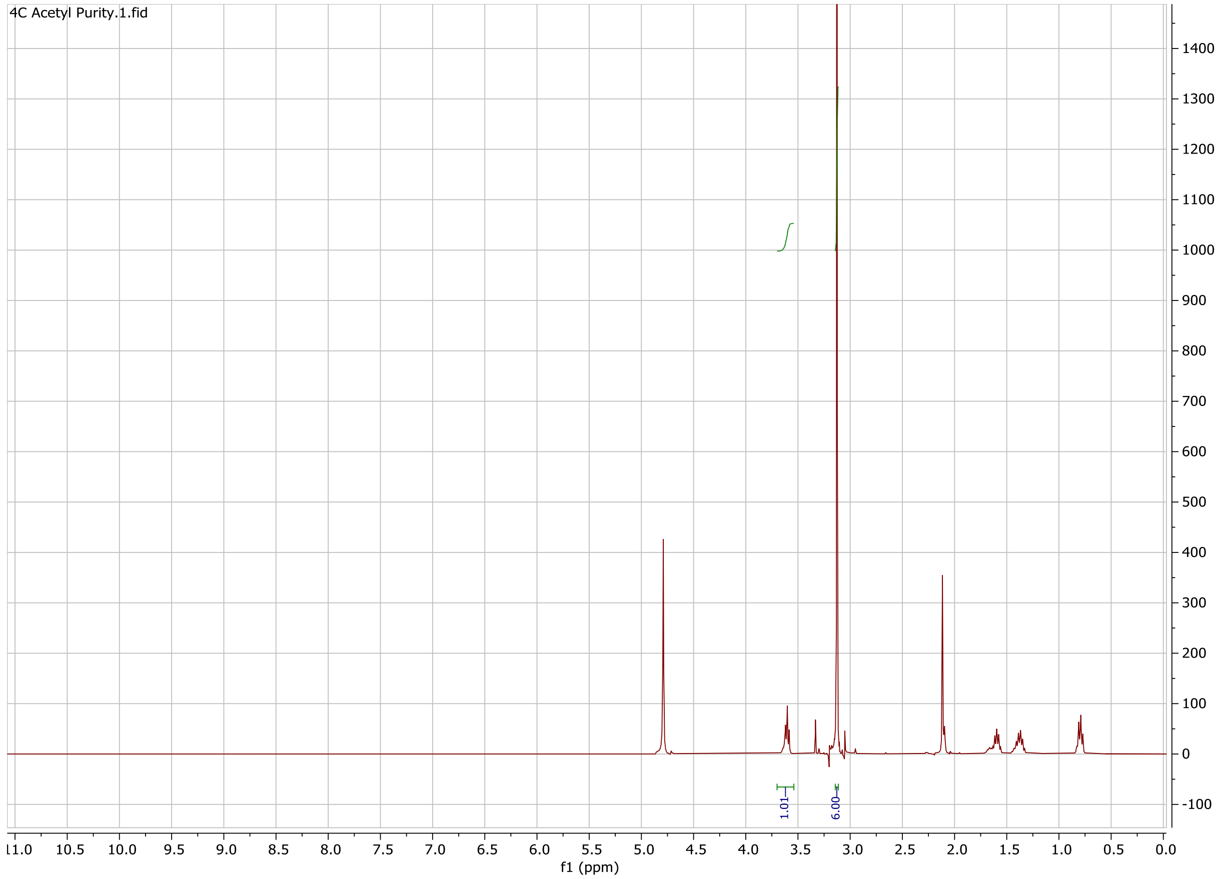

Figure S6. ^{1}H NMR purity spectrum of **2c** with Me_2SO_2 in D_2O.

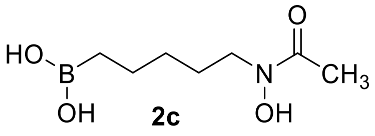

P = 99.6%

$n_{i.c.}$: 6
$Int_{i.c.}$: 6
$MW_{i.c.}$: 94.13 g/mol
$Mass_{i.c.}$: 11.7 mg
$P_{i.c.}$: 99.4%

$n_{s.}$: 2
$Int_{s.}$: 0.70
MW_{s}: 189.02 g/mol
$Mass_{s.}$: 8.2 mg

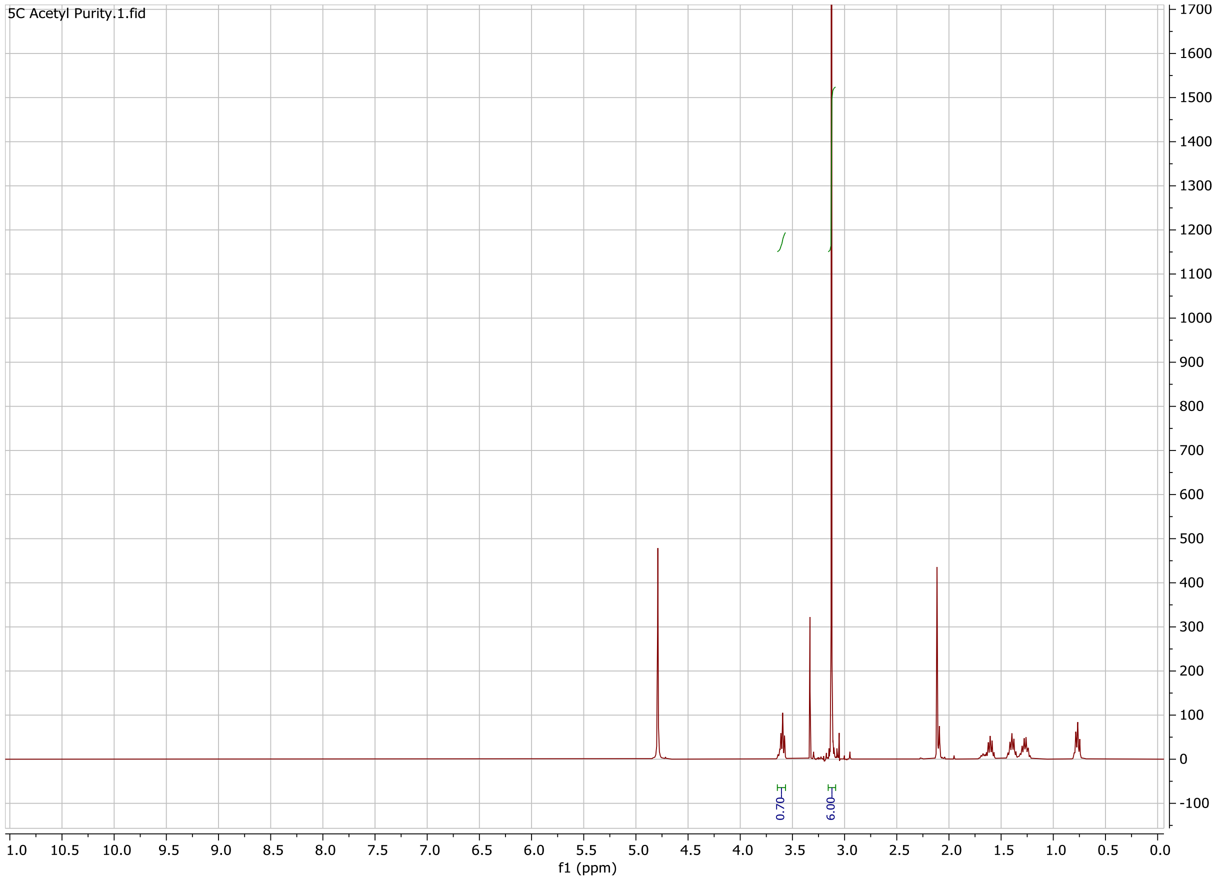

Figure S7. ^{1}H NMR purity spectrum of **3a** with Me_2SO_2 in Acetone-d_6.

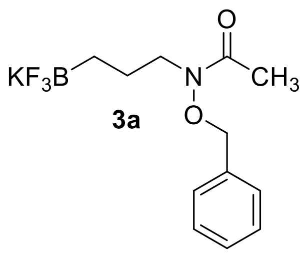

P = 99.4%

$n_{i.c.}$: 6
$Int_{i.c.}$: 6
$MW_{i.c.}$: 94.13 g/mol
$Mass_{i.c.}$: 5.6 mg
$P_{i.c.}$: 99.4%

$n_{s.}$: 2
$Int_{s.}$: 1.16
MW_{s}: 313.17 g/mol
$Mass_{s.}$: 10.8 mg

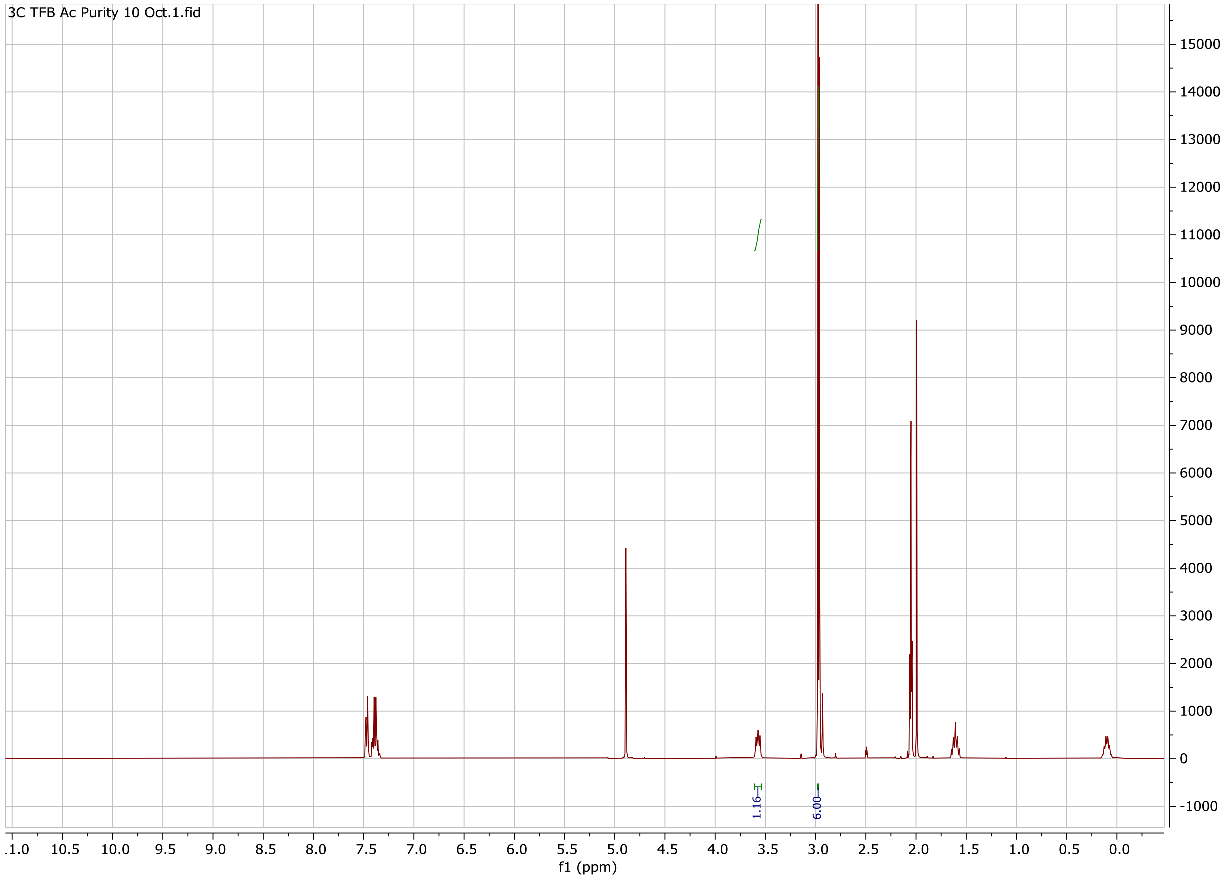

Figure S8. ^{1}H NMR purity spectrum of **3b** with Me_2SO_2 in Acetone-d_6.

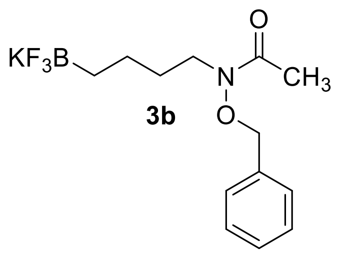

P = 97.6%

$n_{i.c.}$: 6
$Int_{i.c.}$: 6
$MW_{i.c.}$: 94.13 g/mol
$Mass_{i.c.}$: 6.8 mg
$P_{i.c.}$: 99.4%

$n_{s.}$: 2
$Int_{s.}$: 1.18
MW_s: 327.20 g/mol
$Mass_{s.}$: 14.2 mg

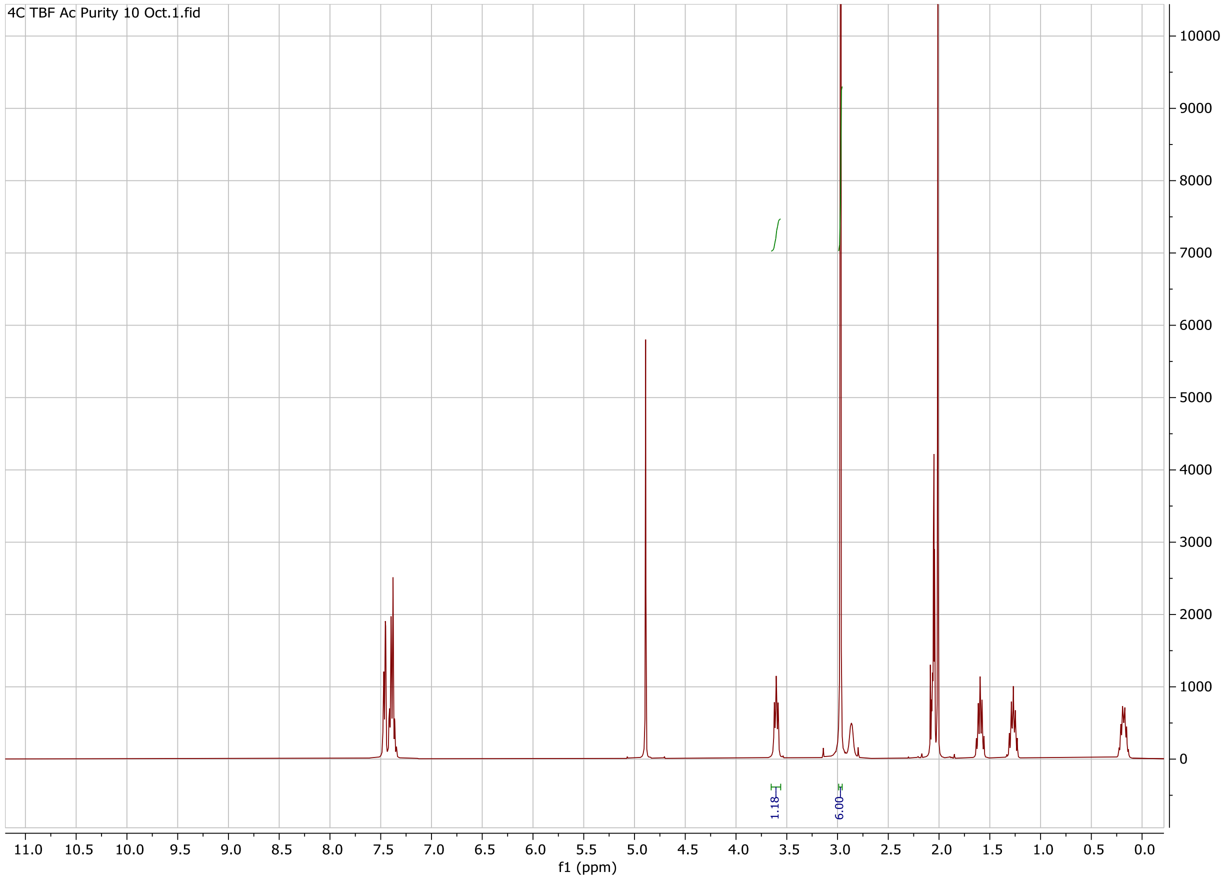

Figure S9. ^{1}H NMR purity spectrum of **4** with Me_2SO_2 in methanol-d$_4$.

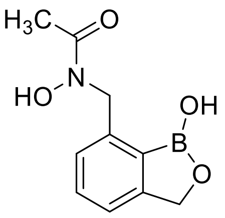

4

P = 95.4%

$n_{i.c.}$: 6
$Int_{i.c.}$: 6
$MW_{i.c.}$: 94.13 g/mol
$Mass_{i.c.}$: 6.2 mg
$P_{i.c.}$: 99.4%

$n_{s.}$: 1
$Int_{s.}$: 0.30333 (Avg)
MW_{s}: 221.02 g/mol
$Mass_{s.}$: 4.6 mg

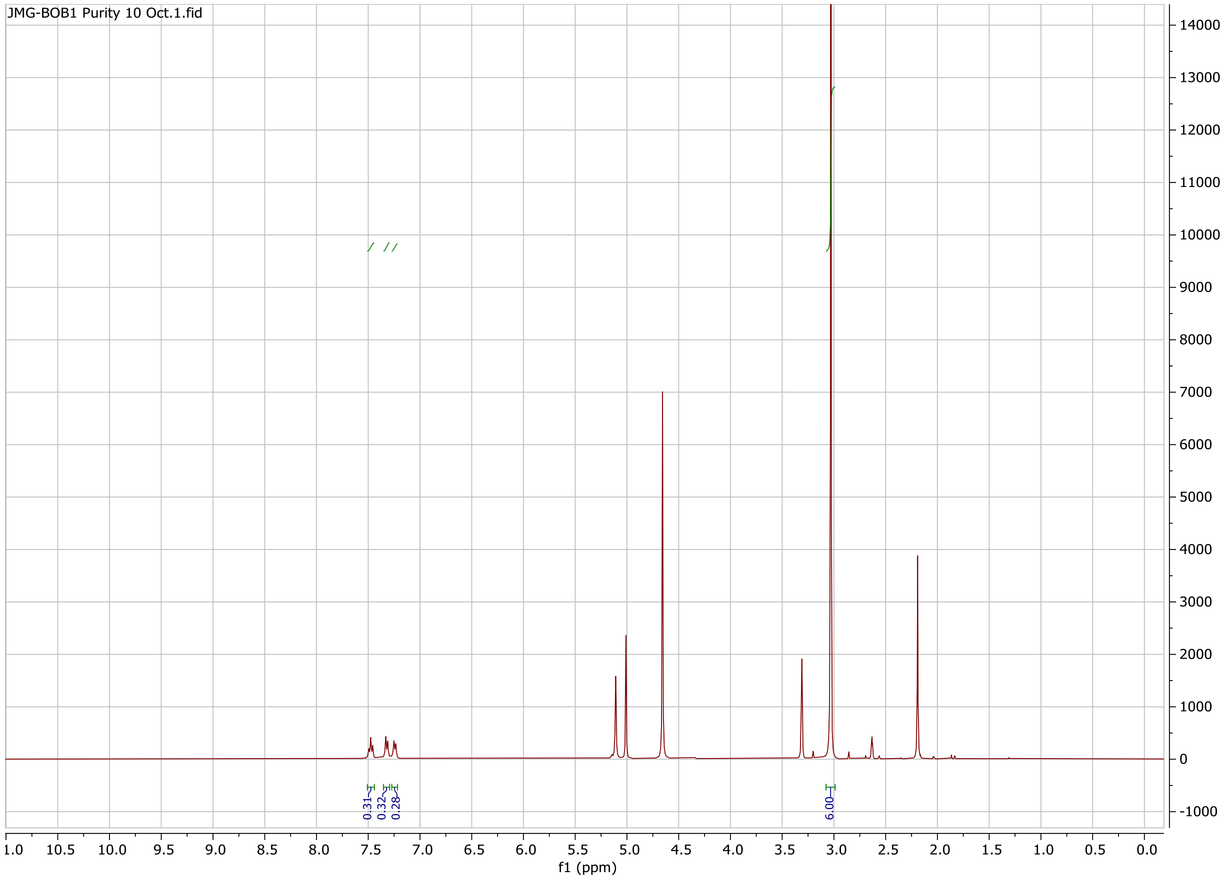

Figure S10. ¹H NMR purity spectrum of **5** with Me_2SO_2 in methanol-d_4.

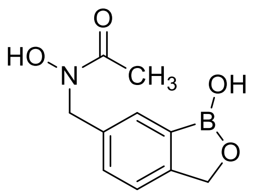

5

P = 97.2%

$n_{i.c.}$: 6
$Int_{i.c.}$: 6
$MW_{i.c.}$: 94.13 g/mol
$Mass_{i.c.}$: 5.1 mg
$P_{i.c.}$: 99.4%

$n_{s.}$: 1
$Int_{s.}$: 0.49 (Avg)
MW_{s}: 221.02 g/mol
$Mass_{s.}$: 6.0 mg

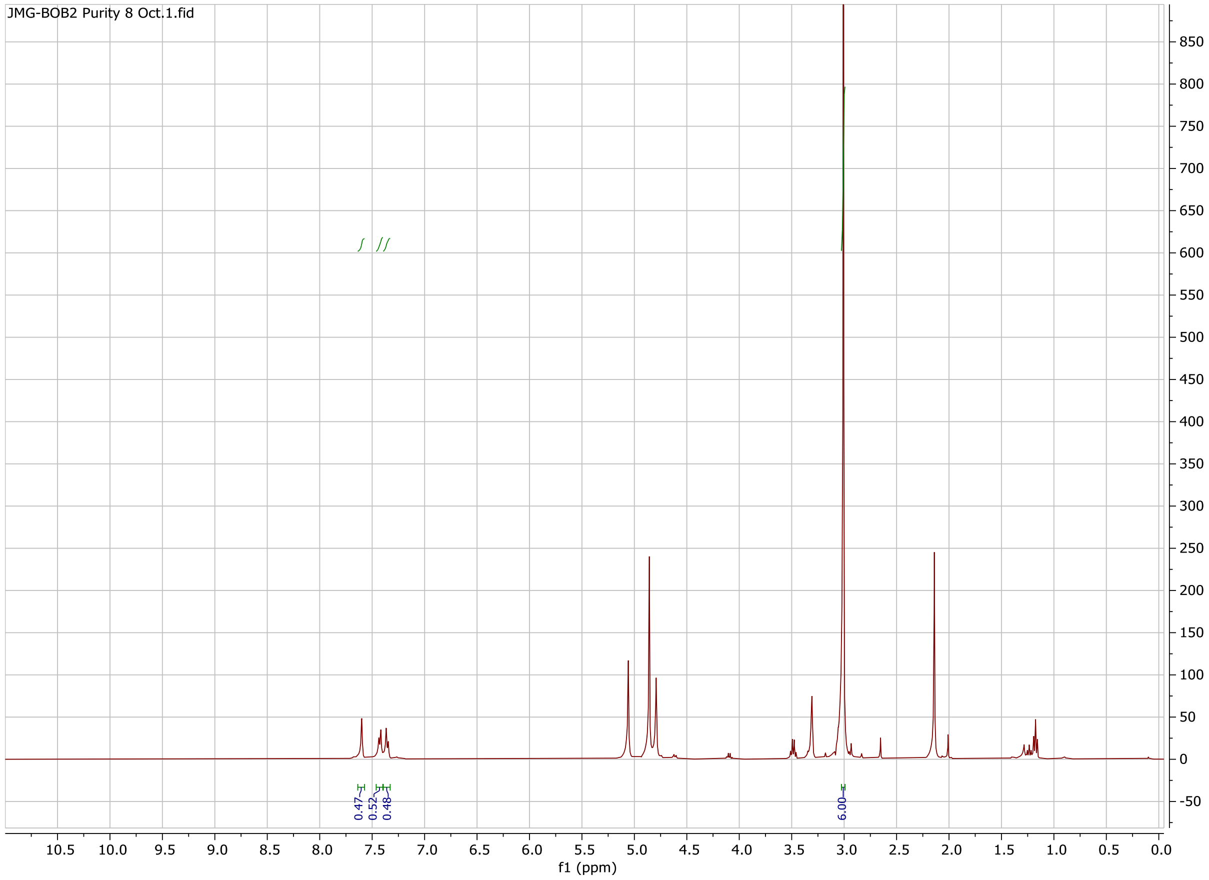

Figure S11. ^{1}H NMR purity spectrum of **6** with Me_2SO_2 in methanol-d_4.

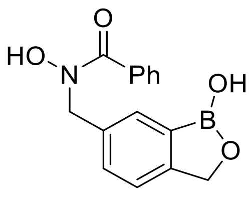

6

P = 95.5%

$n_{i.c.}$: 6
$Int_{i.c.}$: 6
$MW_{i.c.}$: 94.13 g/mol
$Mass_{i.c.}$: 7.0 mg
$P_{i.c.}$: 99.4%

$n_{s.}$: 1
$Int_{s.}$: 0.5025 (Avg)
MW_{s}: 283.09 g/mol
$Mass_{s.}$: 11.0 mg

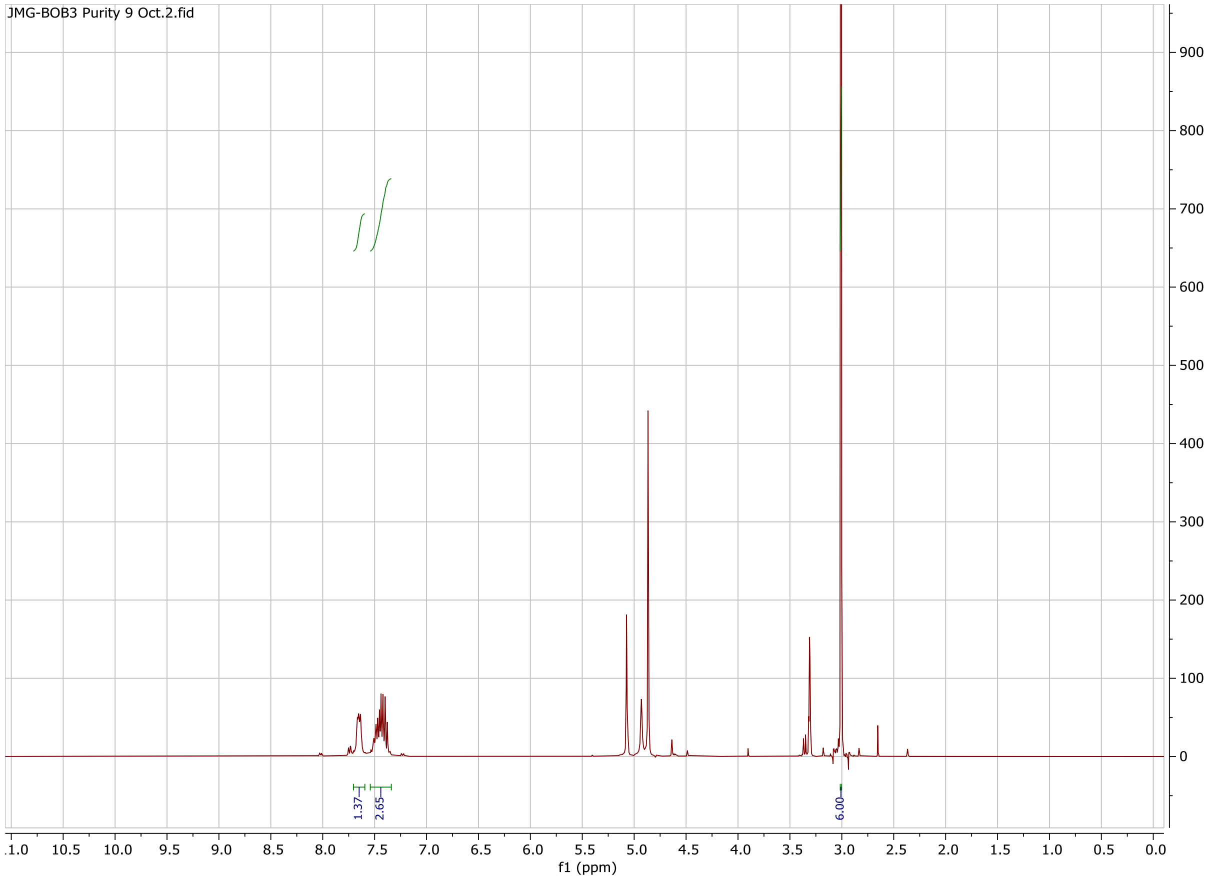

Figure S12. ^{1}H NMR purity spectrum of **7** with Me_2SO_2 in methanol-d$_4$.

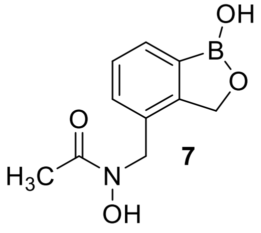

7

P = 98.6%

$n_{i.c.}$: 6
$Int_{i.c.}$: 6
$MW_{i.c.}$: 94.13 g/mol
$Mass_{i.c.}$: 5.9 mg
$P_{i.c.}$: 99.4%

$n_{s.}$: 1
$Int_{s.}$: 0.3366 (Avg)
MW_{s}: 221.02 g/mol
$Mass_{s.}$: 4.7 mg

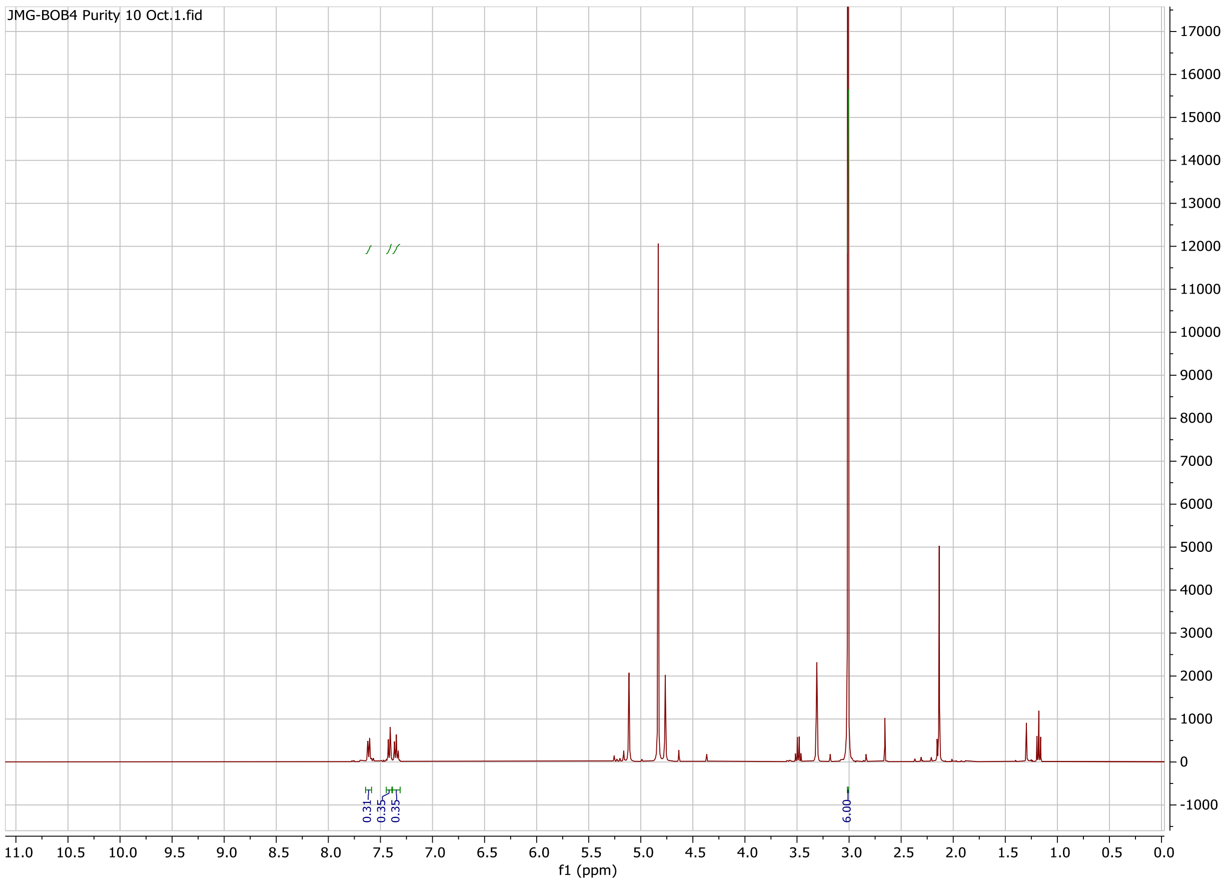

Figure S13. ^{1}H NMR purity spectrum of **8** with Me_2SO_2 in methanol-d_4.

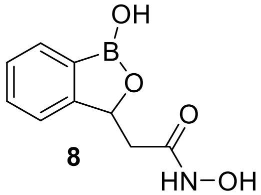

8

P = 96.2%

$n_{i.c.}$: 6
$Int_{i.c.}$: 6
$MW_{i.c.}$: 94.13 g/mol
$Mass_{i.c.}$: 11.8 mg
$P_{i.c.}$: 99.4%

$n_{s.}$: 1
$Int_{s.}$: 047 (Avg)
MW_{s}: 206.99 g/mol
$Mass_{s.}$: 12.6 mg

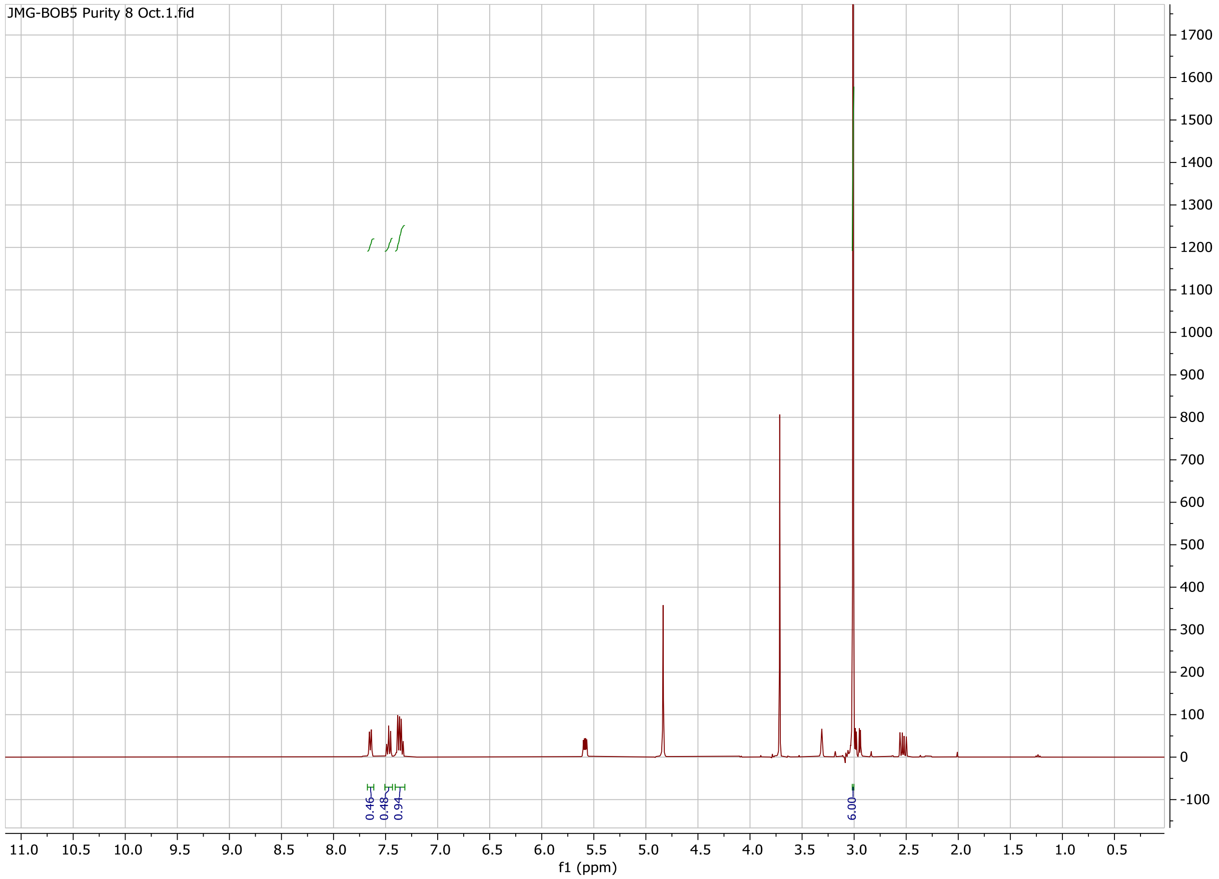

Figure S14. ^{1}H NMR purity spectrum of **9** with Me_2SO_2 in methanol-d$_4$.

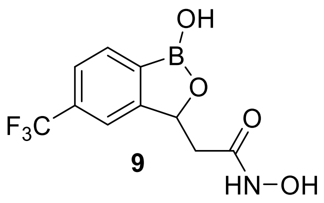

P = 95.3%

$n_{i.c.}$: 6
$Int_{i.c.}$: 6
$MW_{i.c.}$: 94.13 g/mol
$Mass_{i.c.}$: 5.7 mg
$P_{i.c.}$: 99.4%

$n_{s.}$: 1
$Int_{s.}$: 0.53 (Avg)
MW_{s}: 274.99 g/mol
$Mass_{s.}$: 9.2 mg

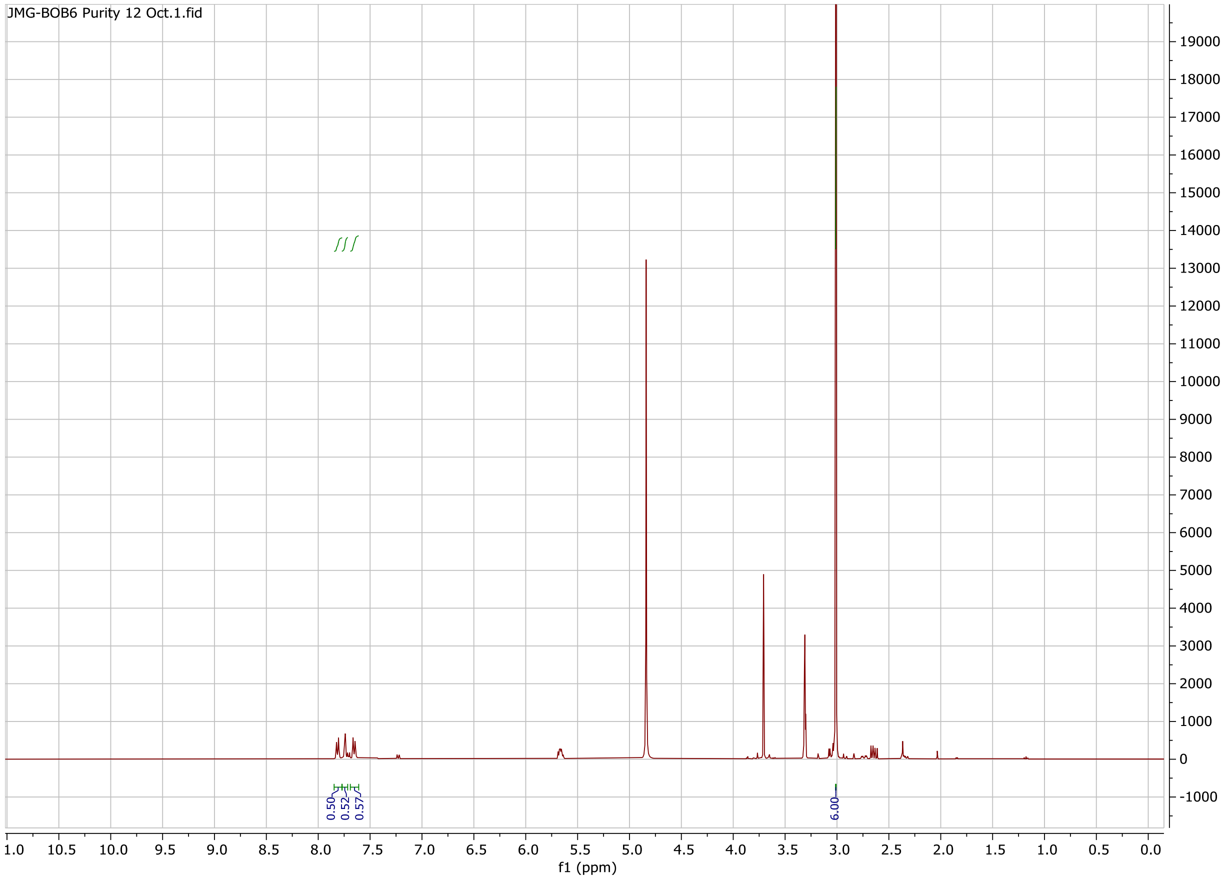

Figure S15. ^{1}H NMR purity spectrum of **10** with Me_2SO_2 in methanol-d_4.

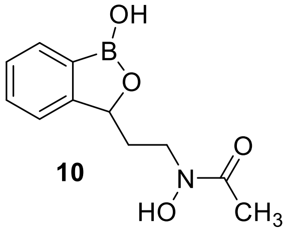

10

P = 86.5%

$n_{i.c.}$: 6
$Int_{i.c.}$: 6
$MW_{i.c.}$: 94.13 g/mol
$Mass_{i.c.}$: 5.0 mg
$P_{i.c.}$: 99.4%

$n_{s.}$: 1
$Int_{s.}$: 0.3975 (Avg)
MW_{s}: 235.05 g/mol
$Mass_{s.}$: 5.7 mg

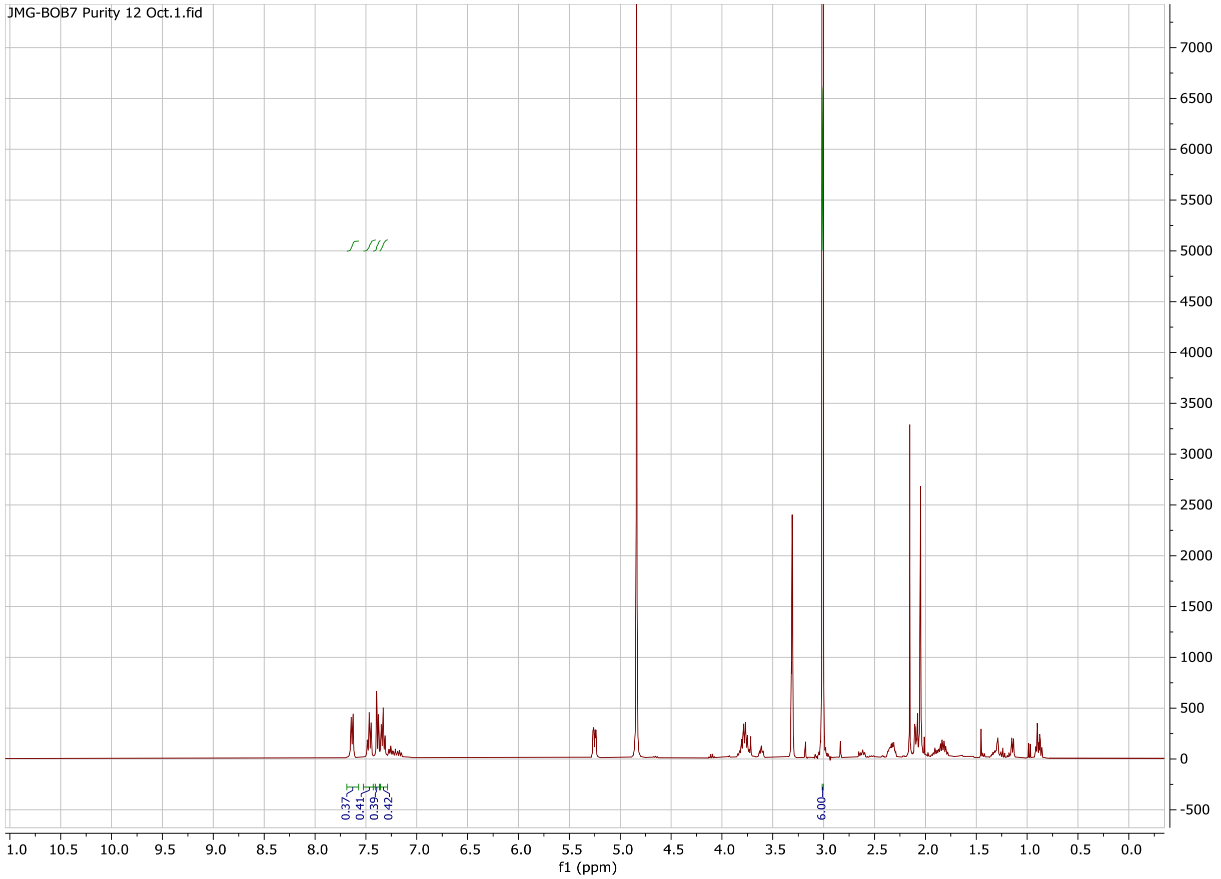

NMR Spectra of Compound Library

Figure S16. ^{1}H NMR spectrum of **1a** in D_2O.

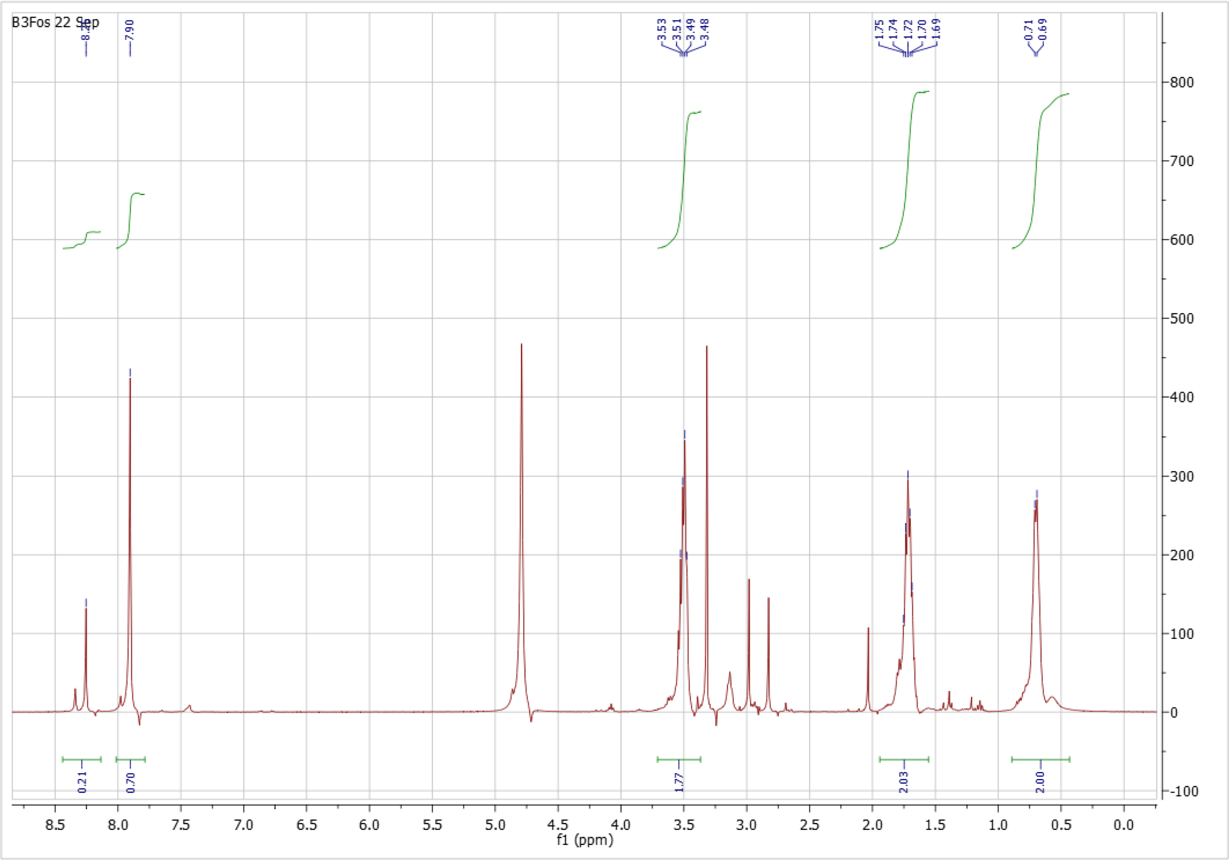

Figure S17. ^{13}C APT NMR spectrum of **1a** in D_2O.

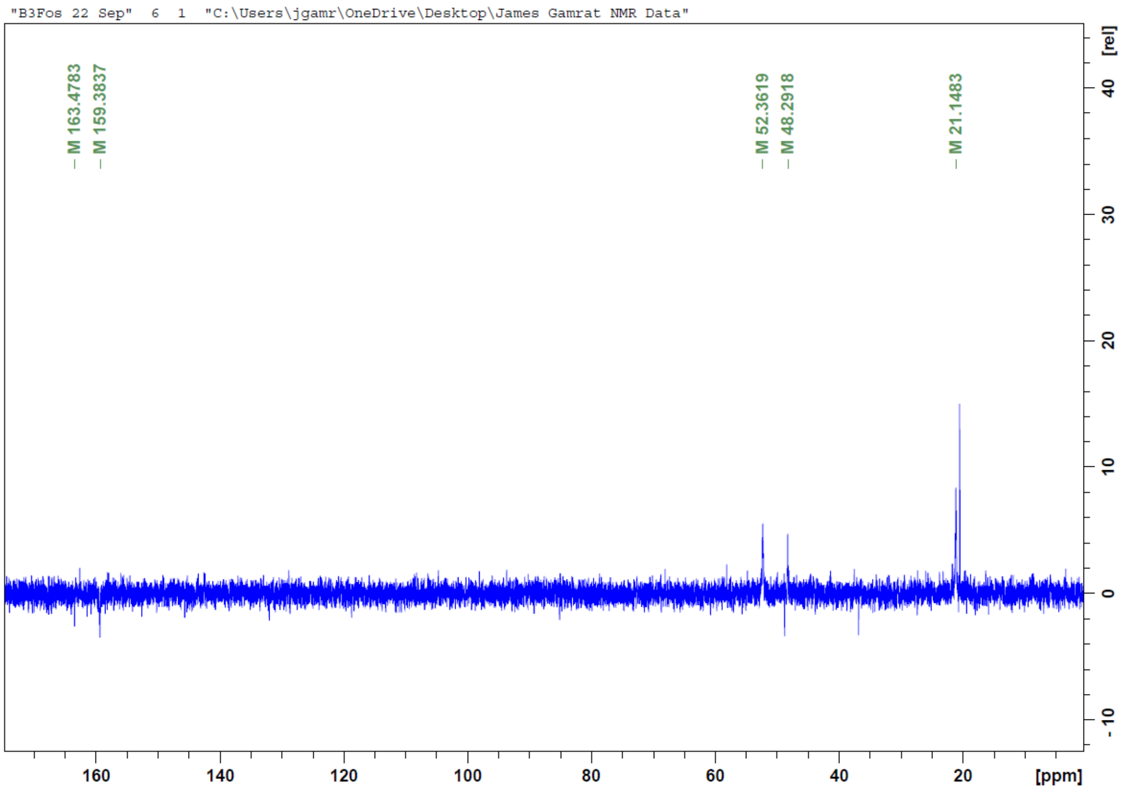

Figure S18. ^{11}B NMR spectrum of **1a** in D_2O.

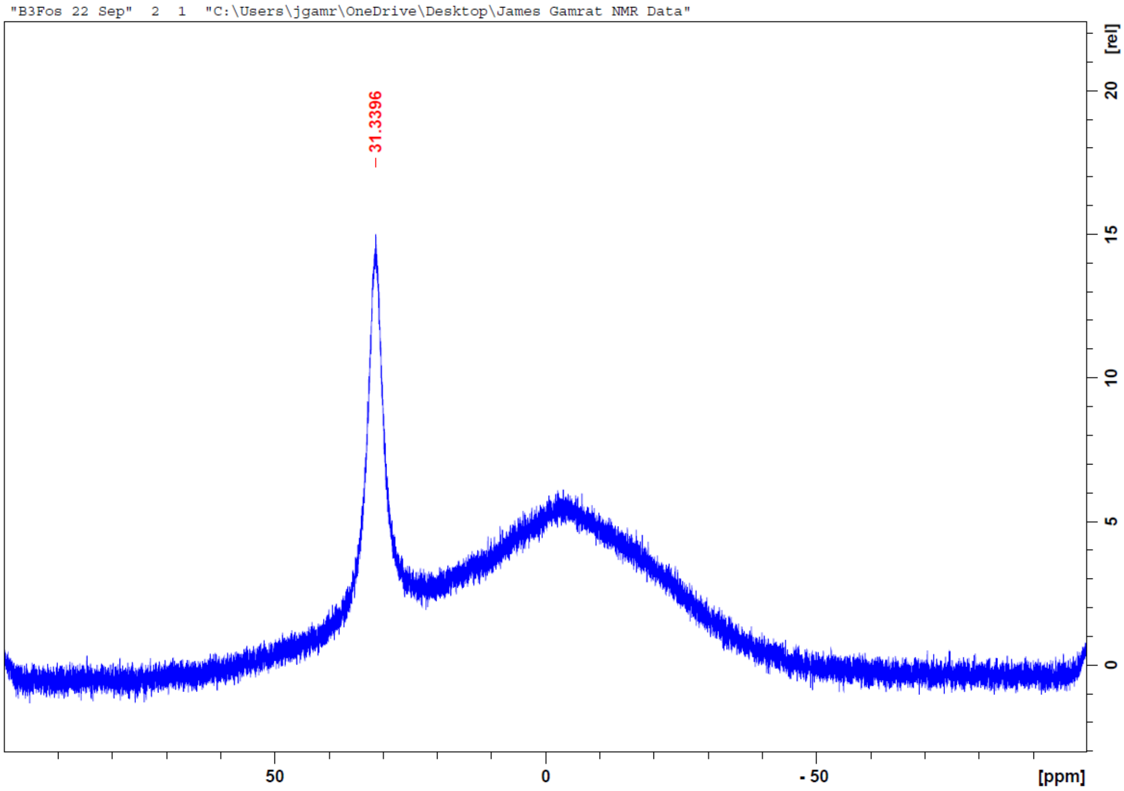

Figure S19. ^{1}H NMR spectrum of **1b** in D_2O.

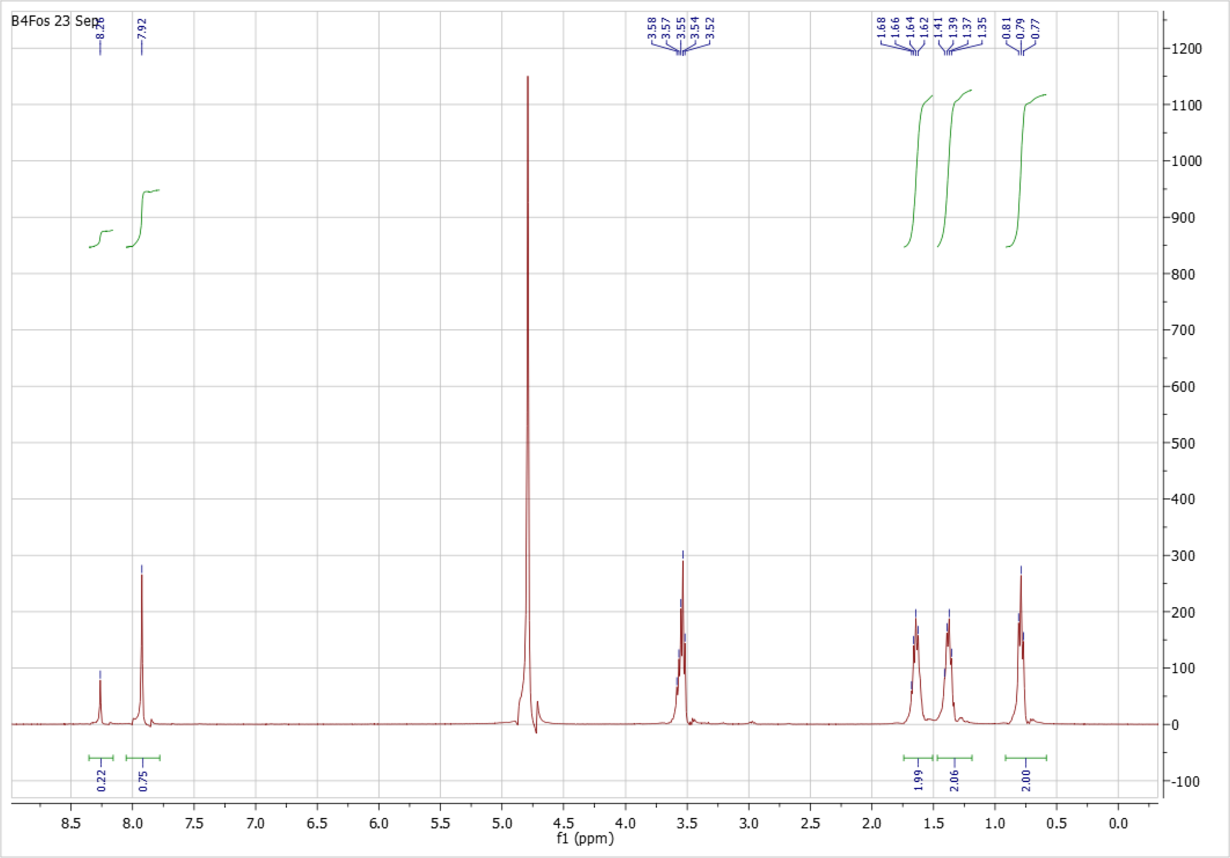

Figure S20. ^{13}C APT NMR spectrum of **1b** in D_2O.

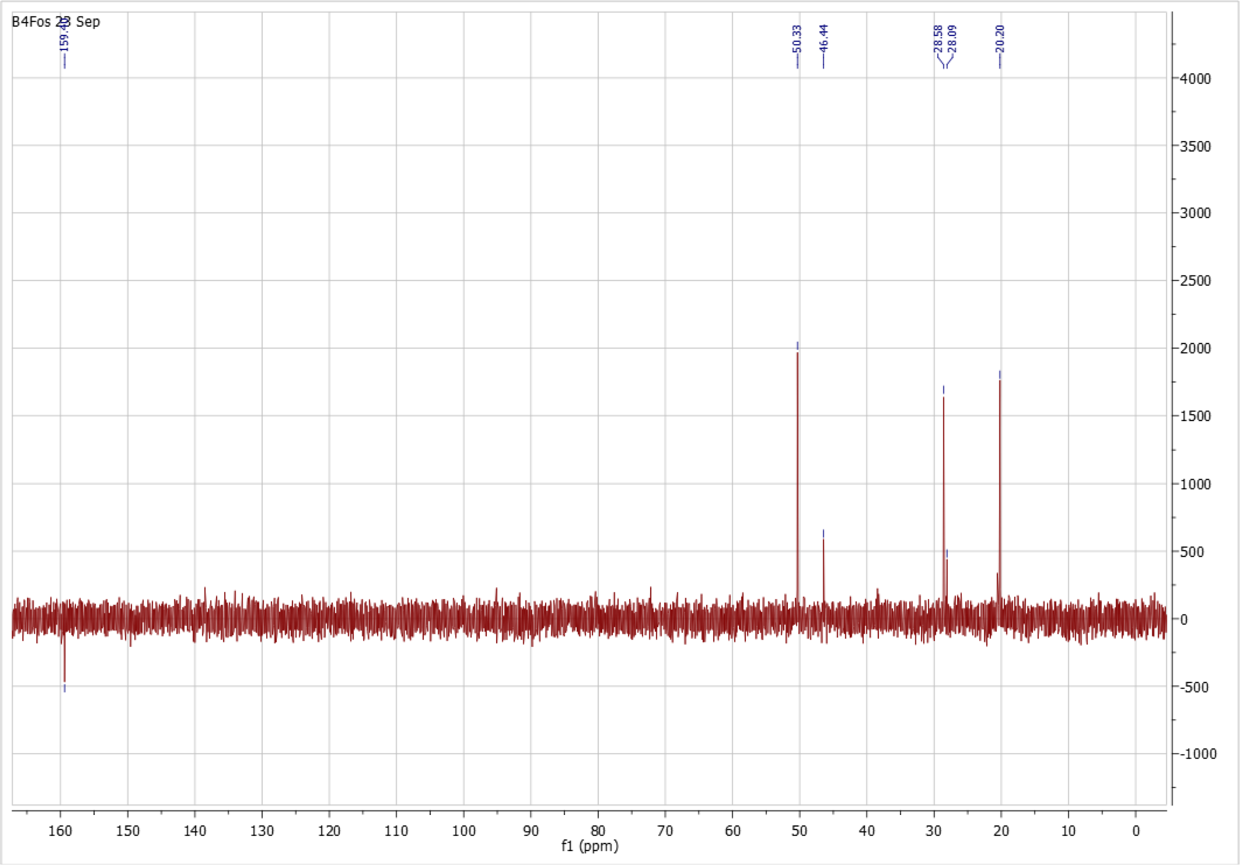

Figure S21. ^{11}B NMR spectrum of **1b** in D_2O.

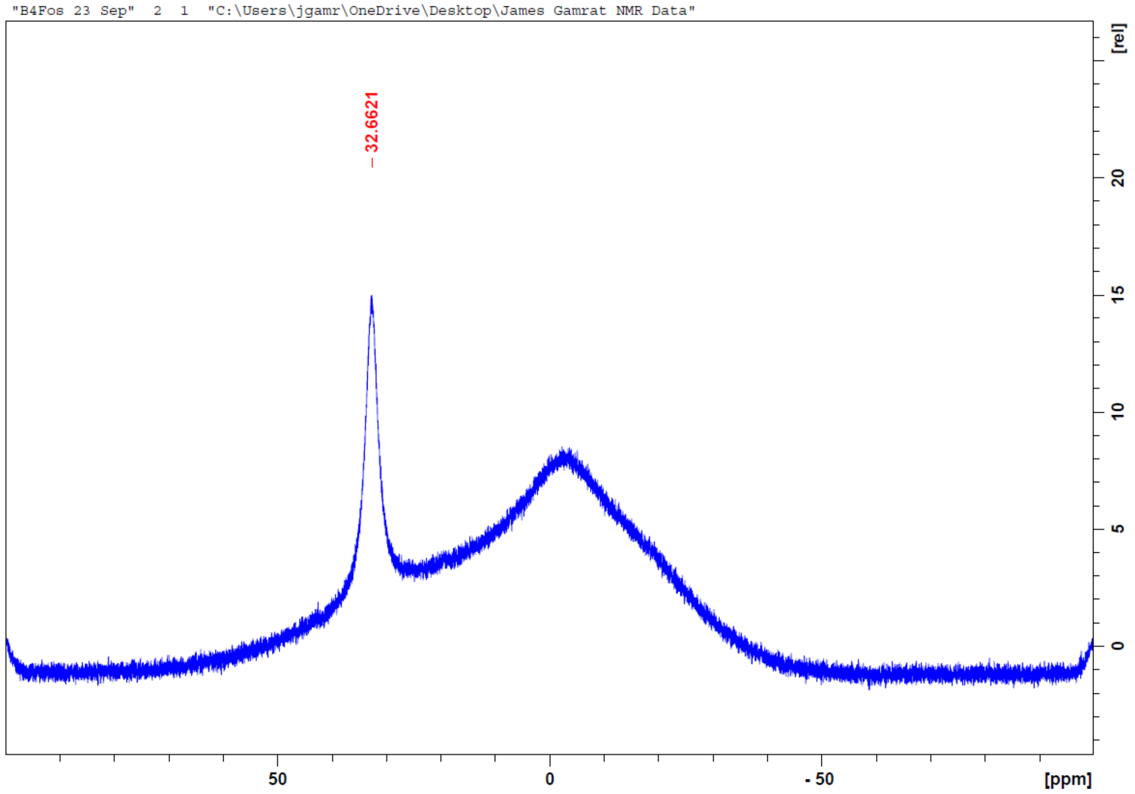

Figure S22. ^{1}H NMR spectrum of **1c** in D_2O.

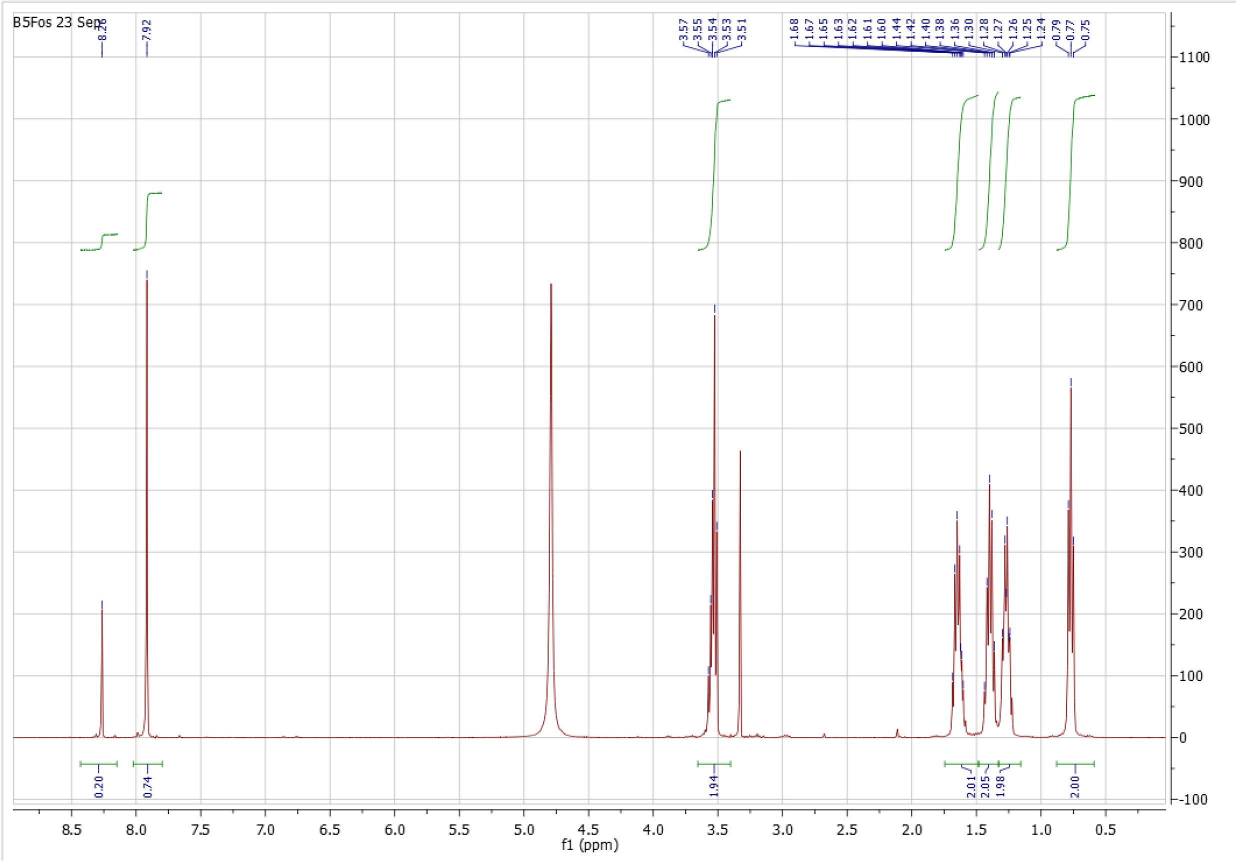

Figure S23. ^{13}C APT NMR spectrum of **1c** in D_2O.

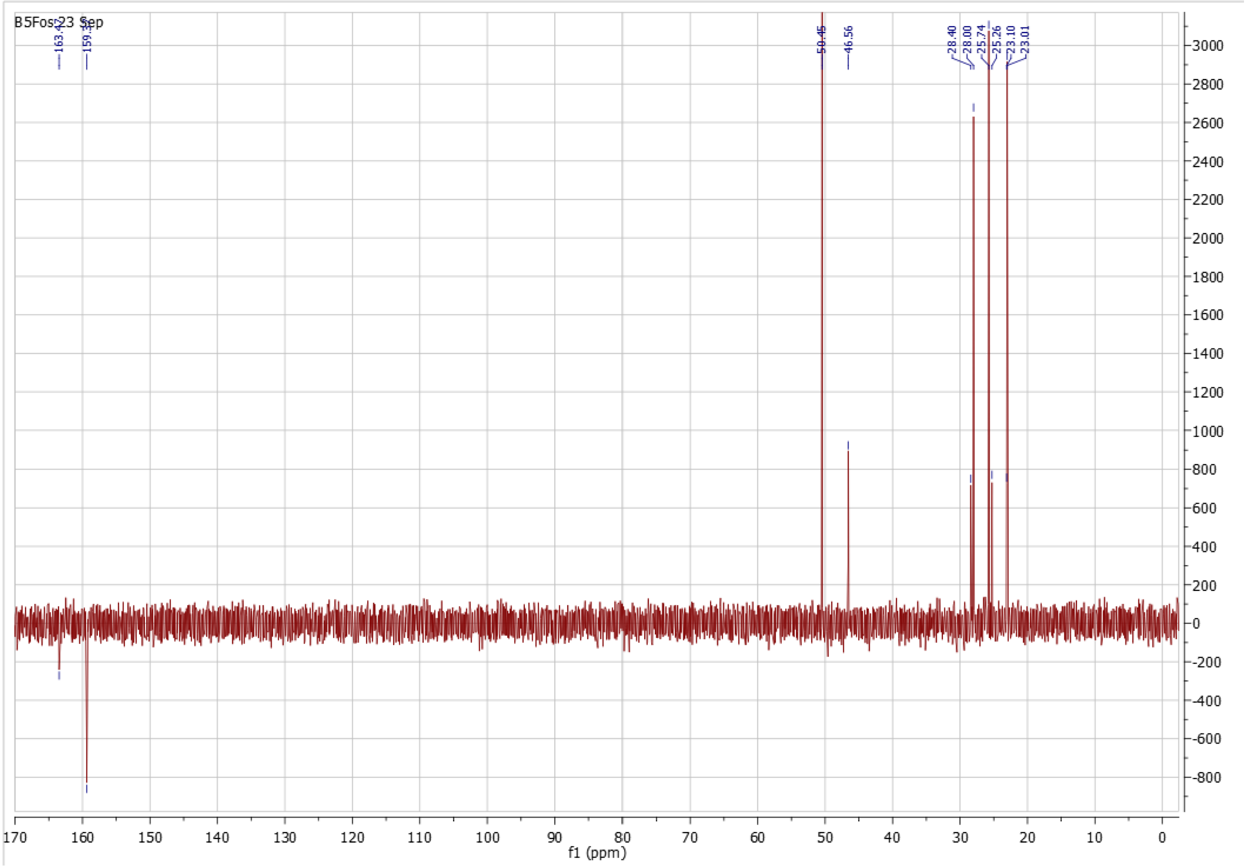

Figure S24. ^{11}B NMR spectrum of **1c** in D_2O.

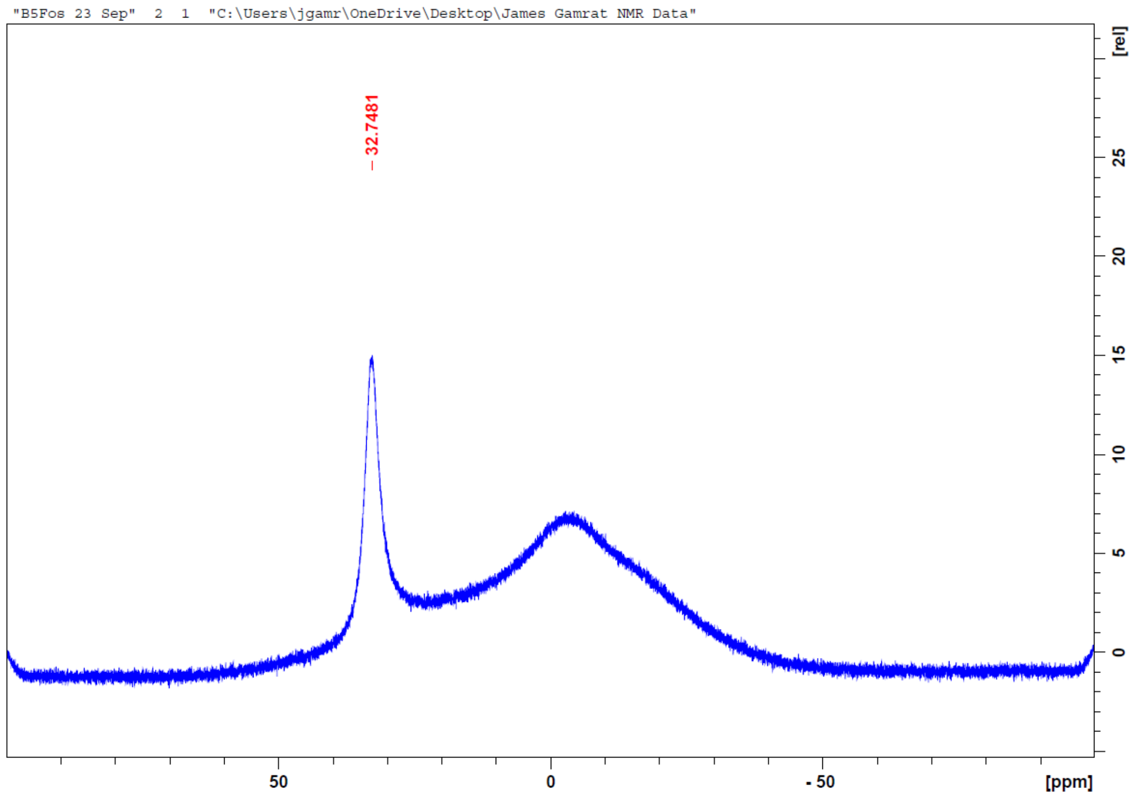

Figure S25. ^1H NMR spectrum of **2a** in D_2O.

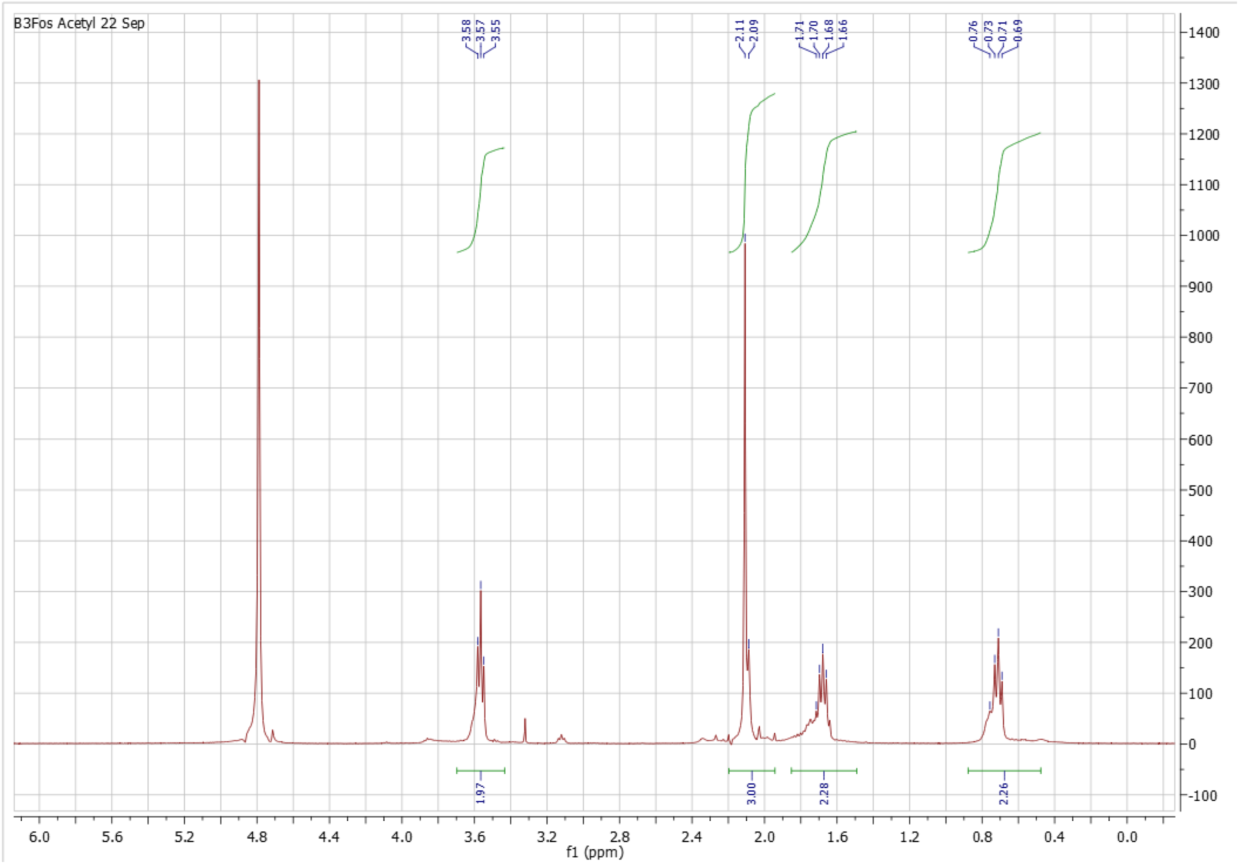

Figure S26. ^{13}C APT NMR spectrum of **2a** in D_2O.

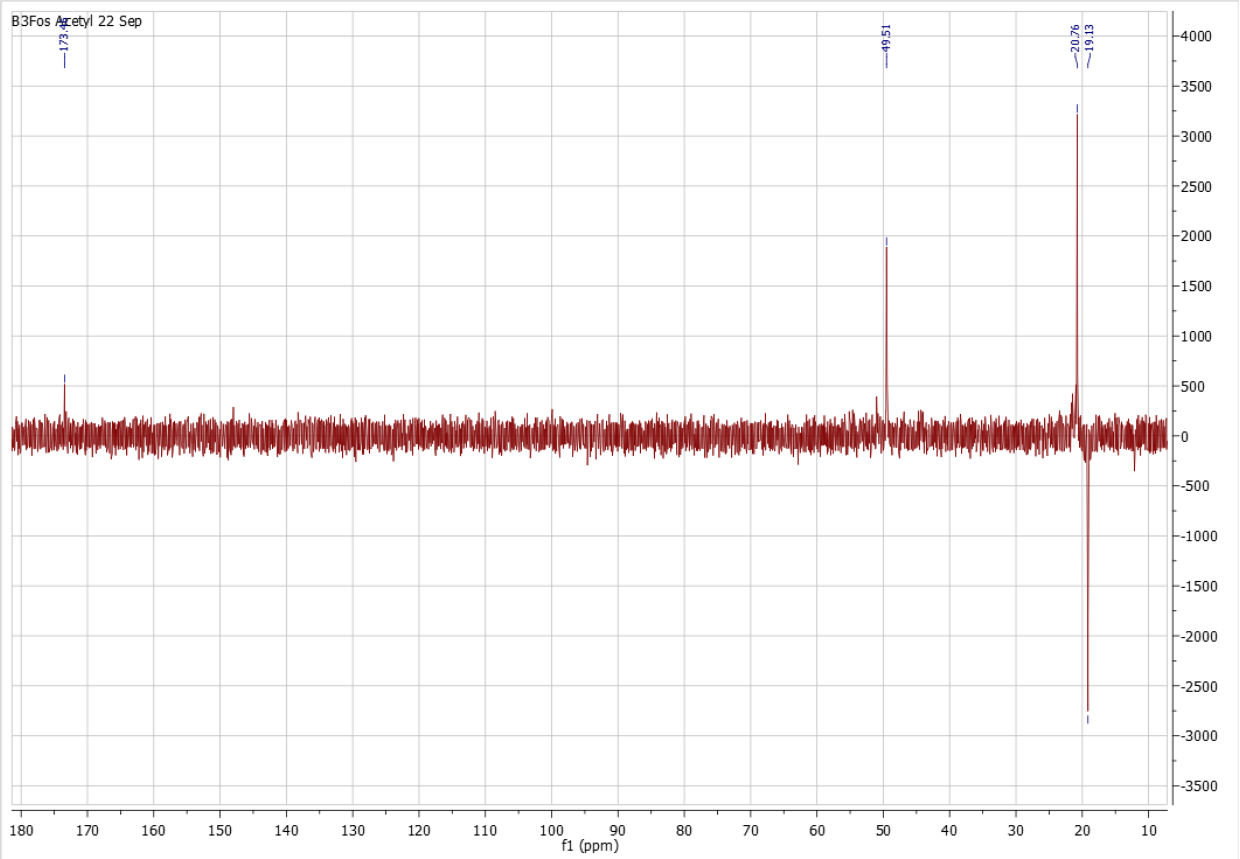

Figure S27. ^{11}B NMR spectrum of **2a** in D_2O.

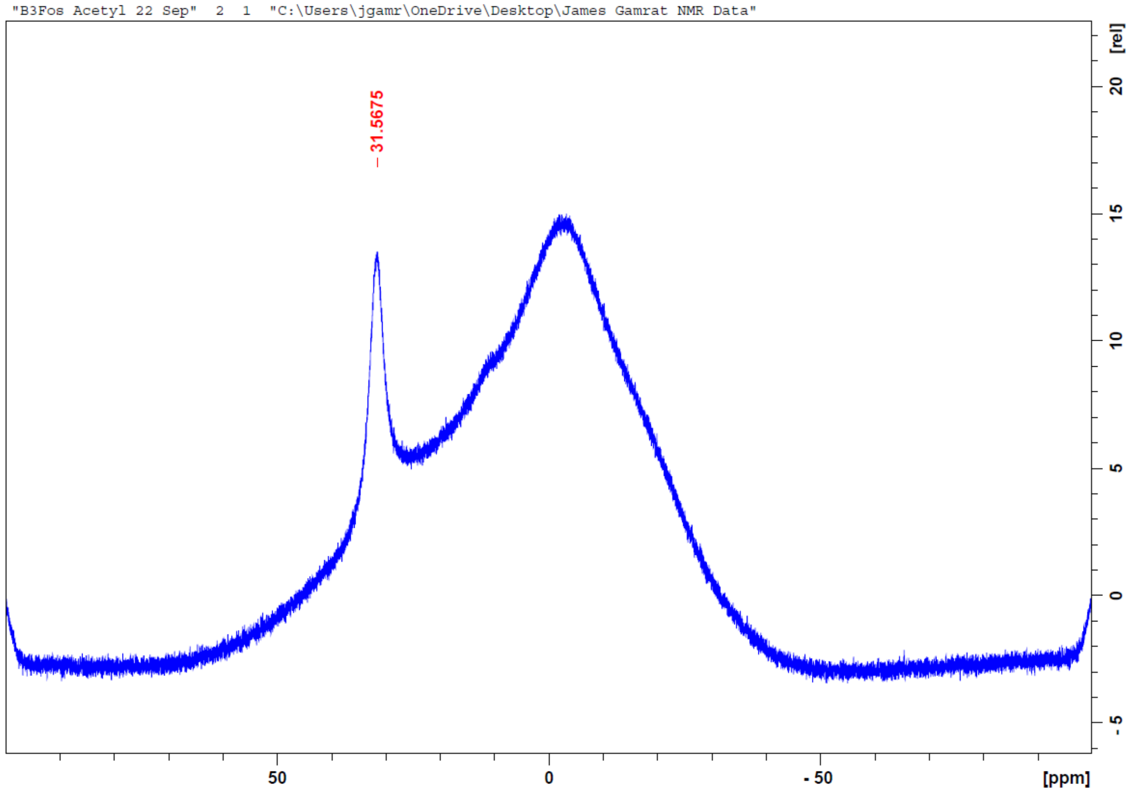

Figure S28. ^{1}H NMR spectrum of **2b** in D_2O.

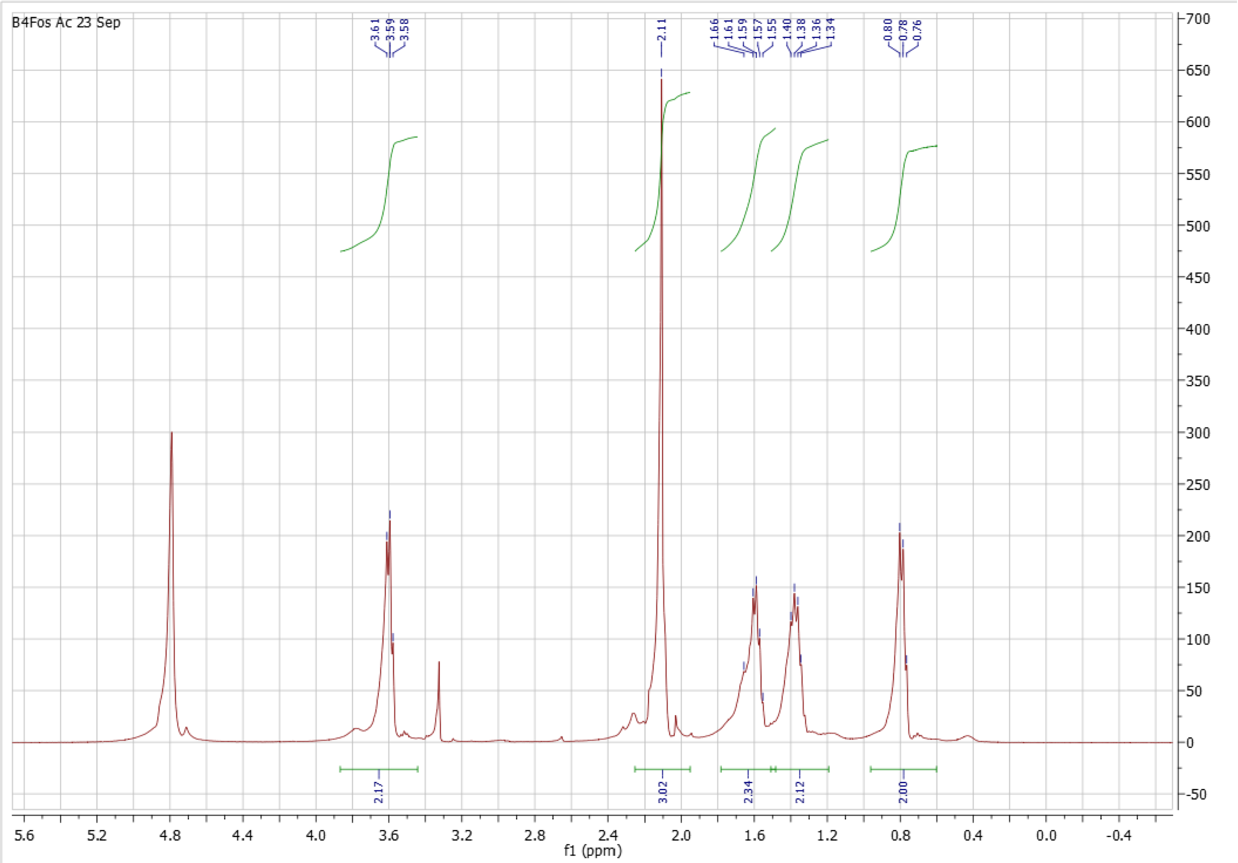

Figure S29. ^{13}C APT NMR spectrum of **2b** in D_2O.

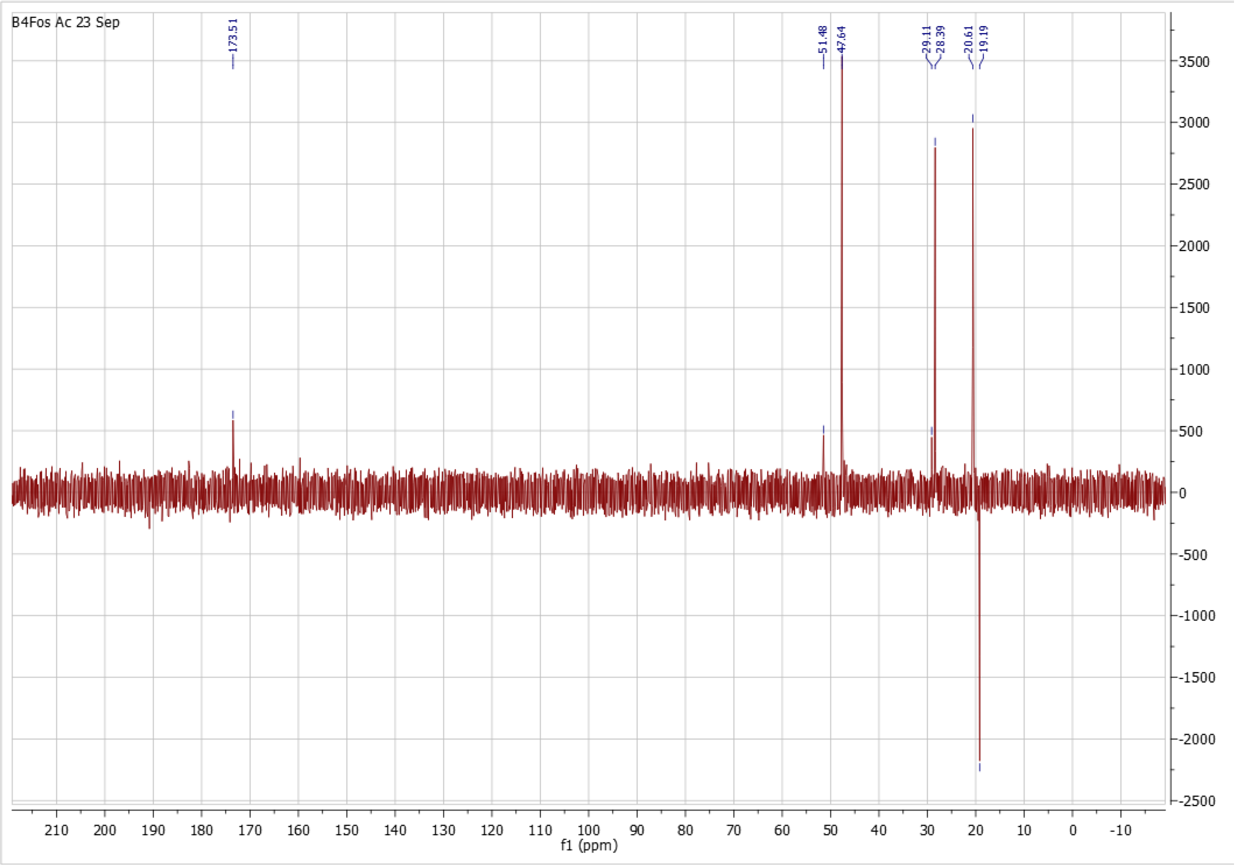

Figure S30. ^{11}B NMR spectrum of **2b** in D_2O.

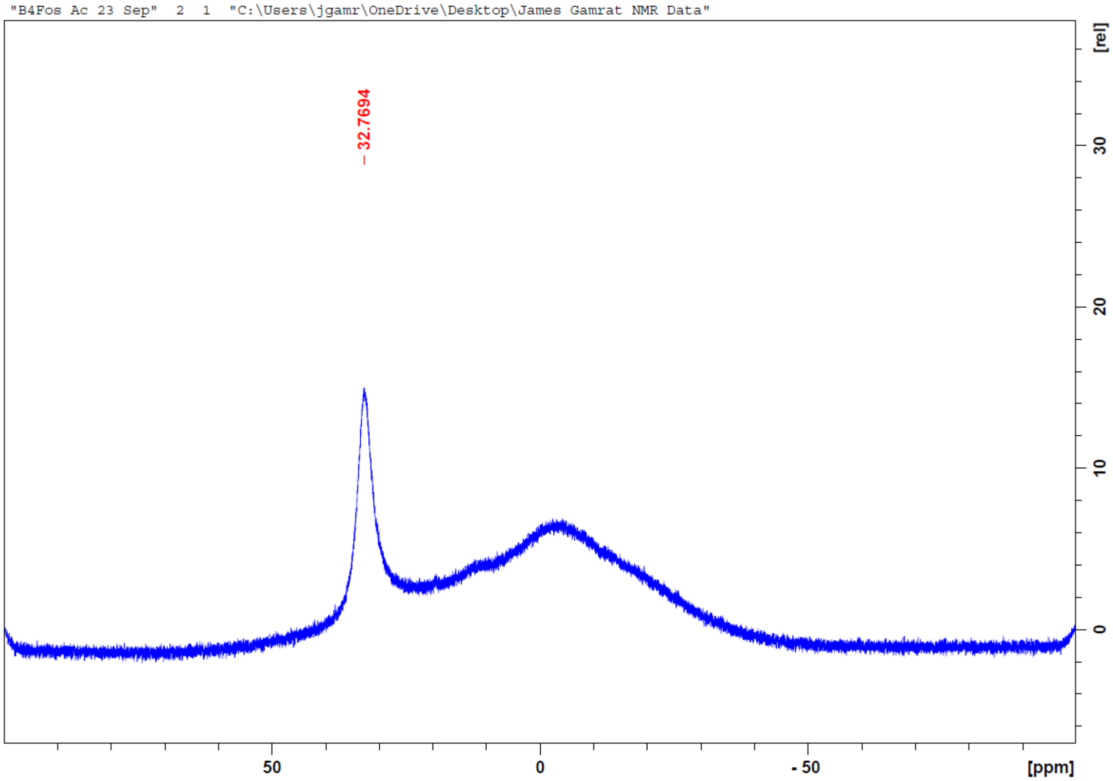

Figure S31. ^{1}H NMR spectrum of **2c** in D_2O.

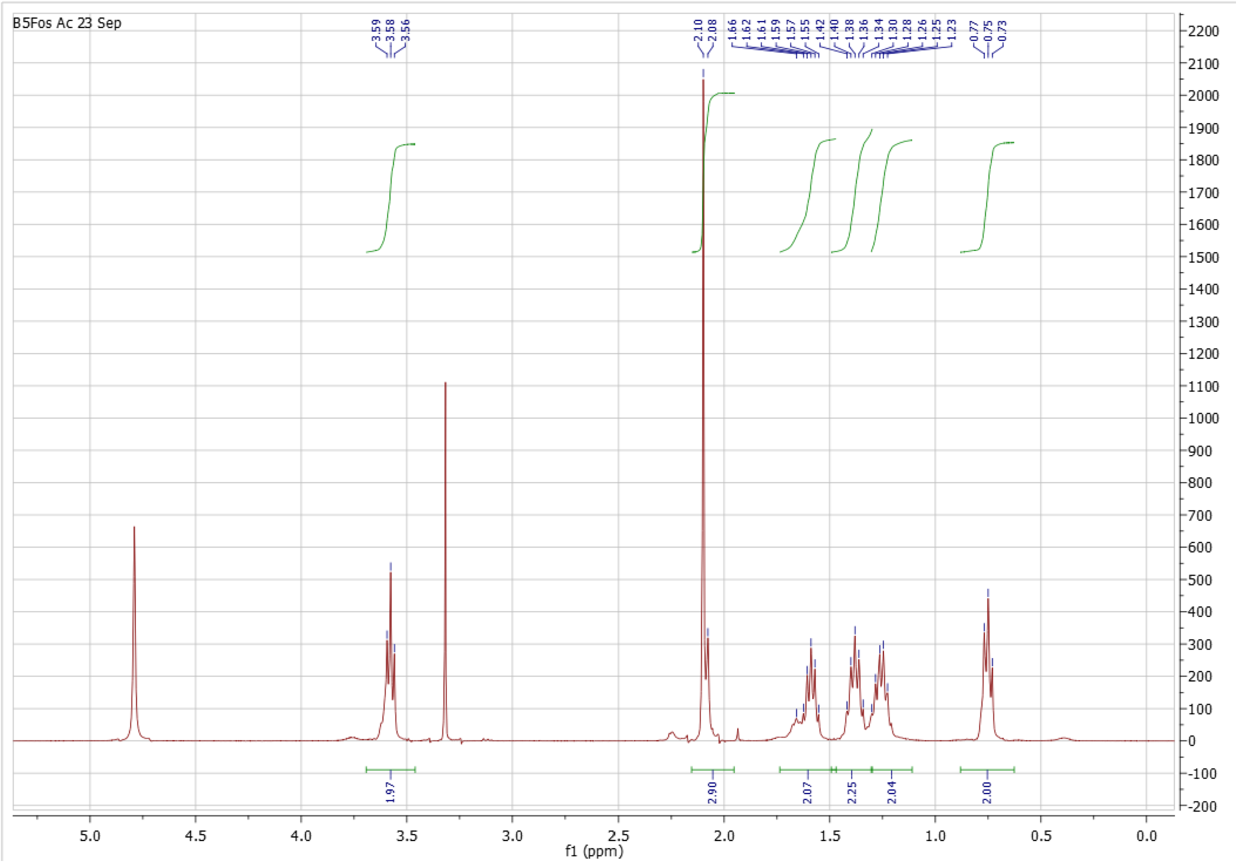

Figure S32. ^{13}C APT NMR spectrum of **2c** in D_2O.

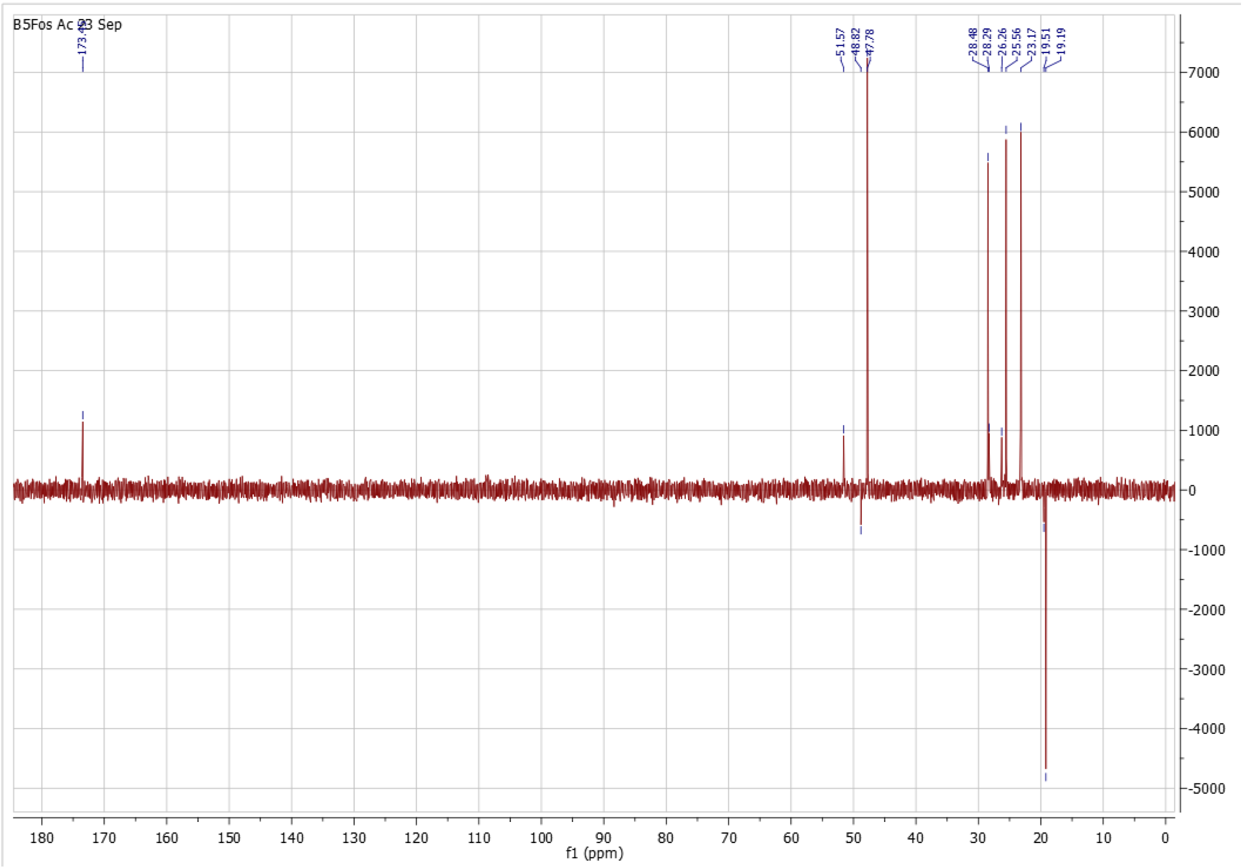

Figure S33. ^{11}B NMR spectrum of **2c** in D_2O.

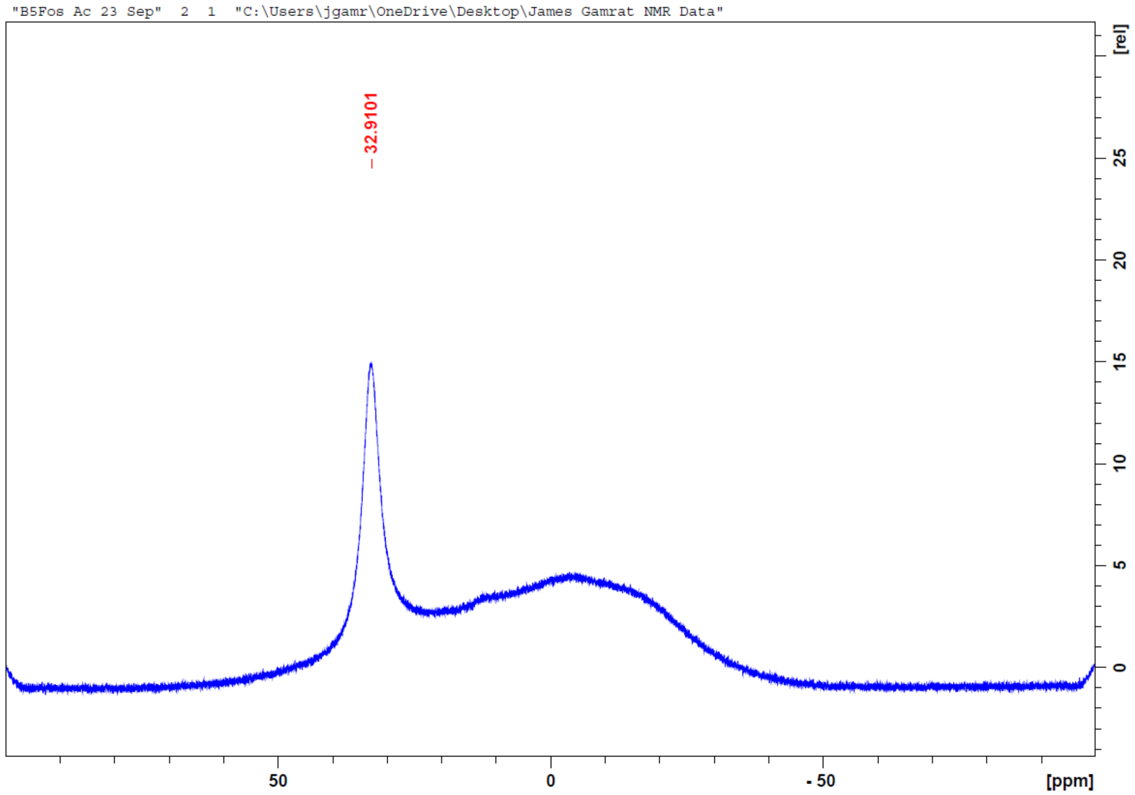

Figure S34. ^{1}H NMR spectrum of **3a** in Acetone-d_6.

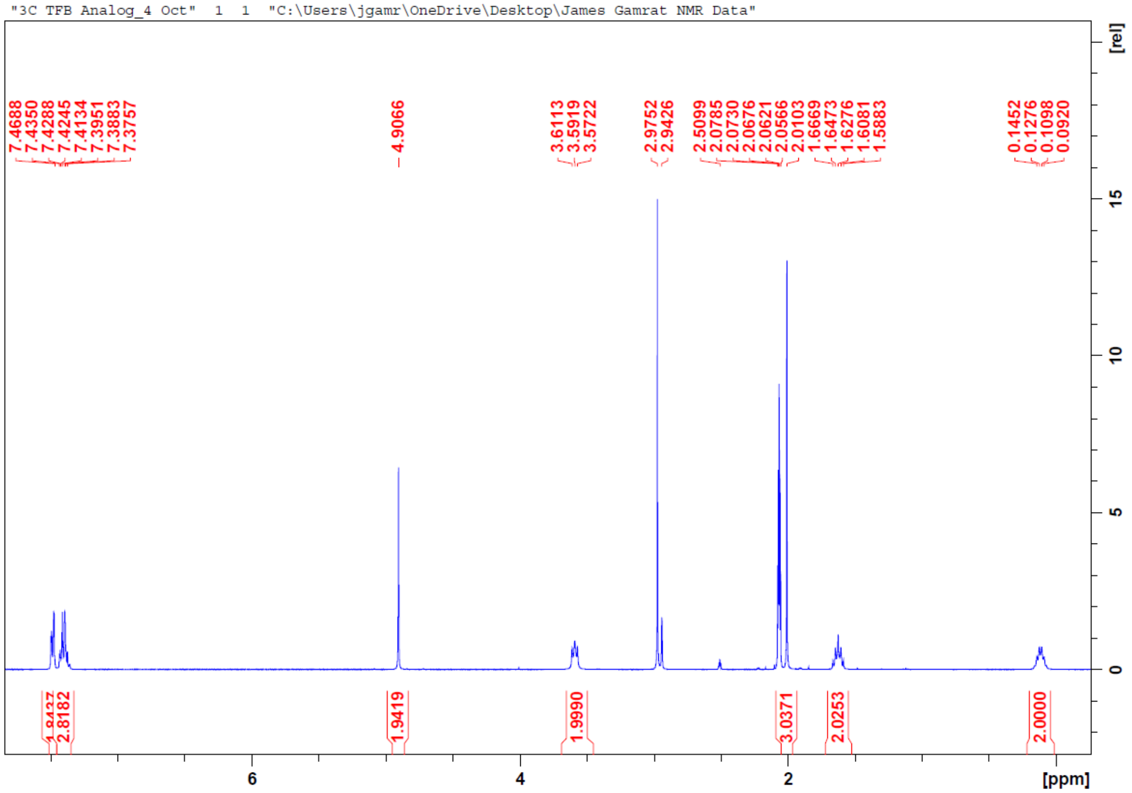

Figure S35. ^{13}C NMR spectrum of **3a** in Acetone-d_6.

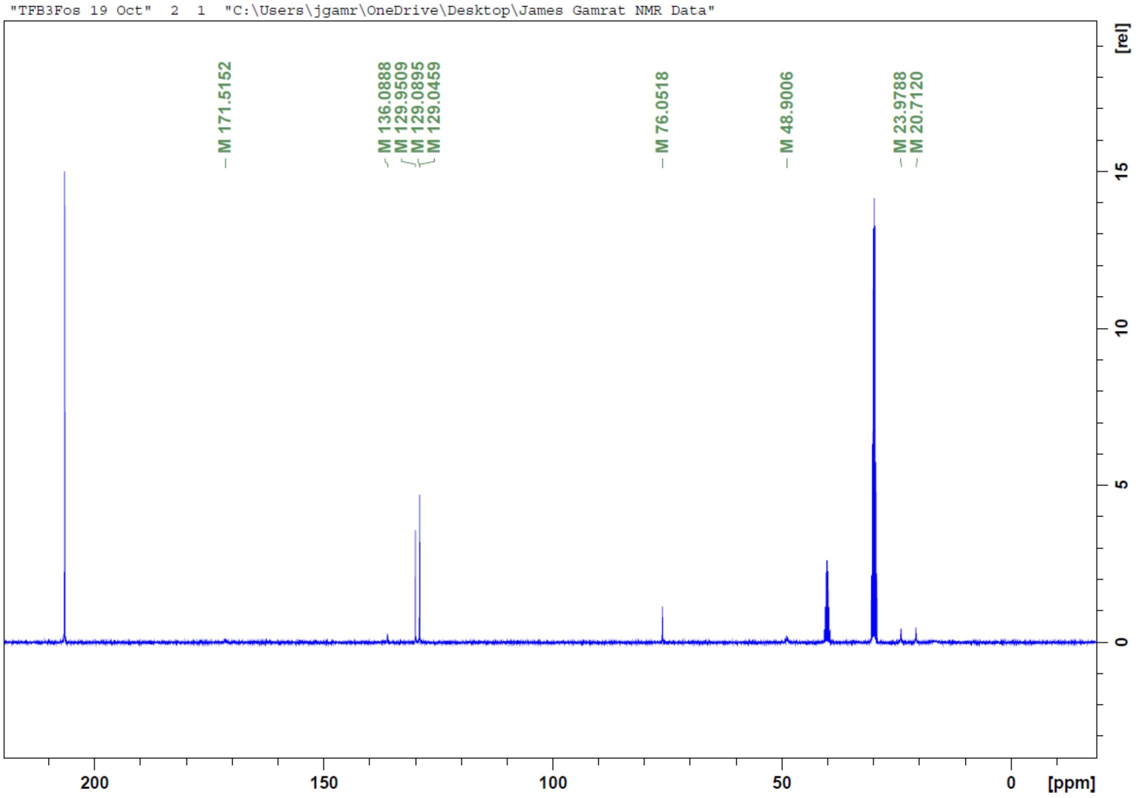

Figure S36. ^{11}B NMR spectrum of **3a** in Acetone-d_6.

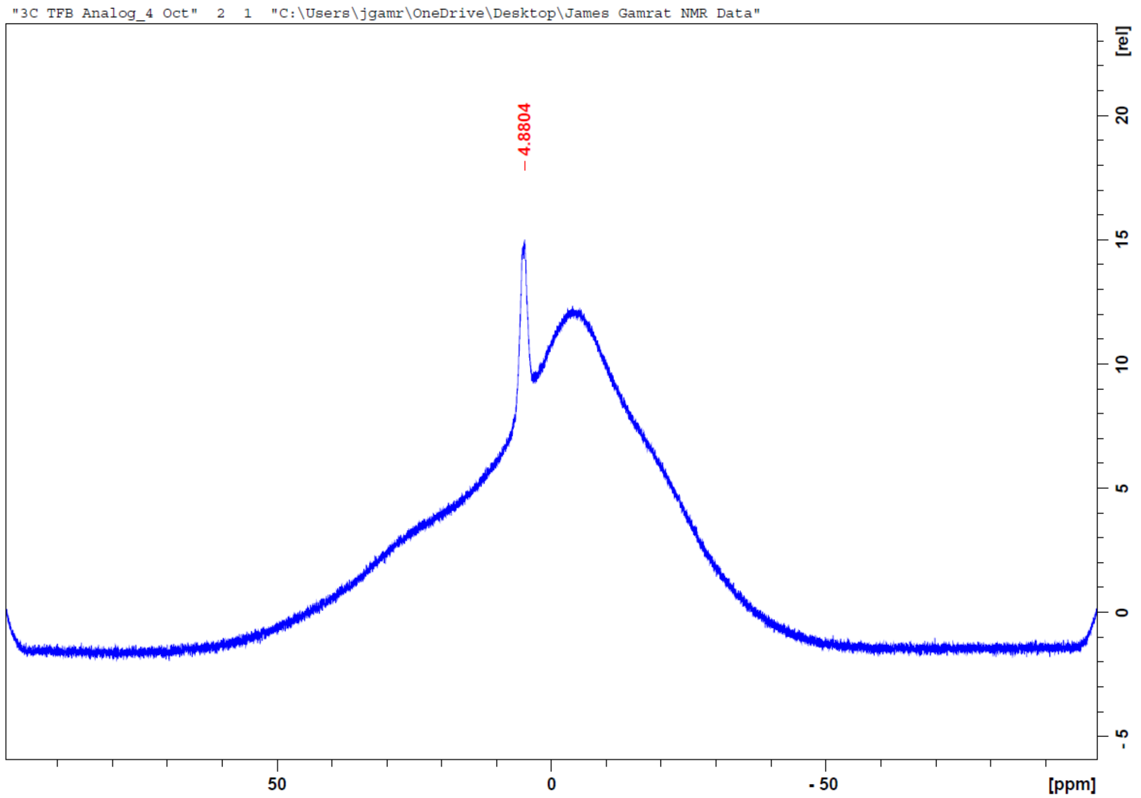

Figure S37. ^{1}H NMR spectrum of **3b** in Acetone-d_6.

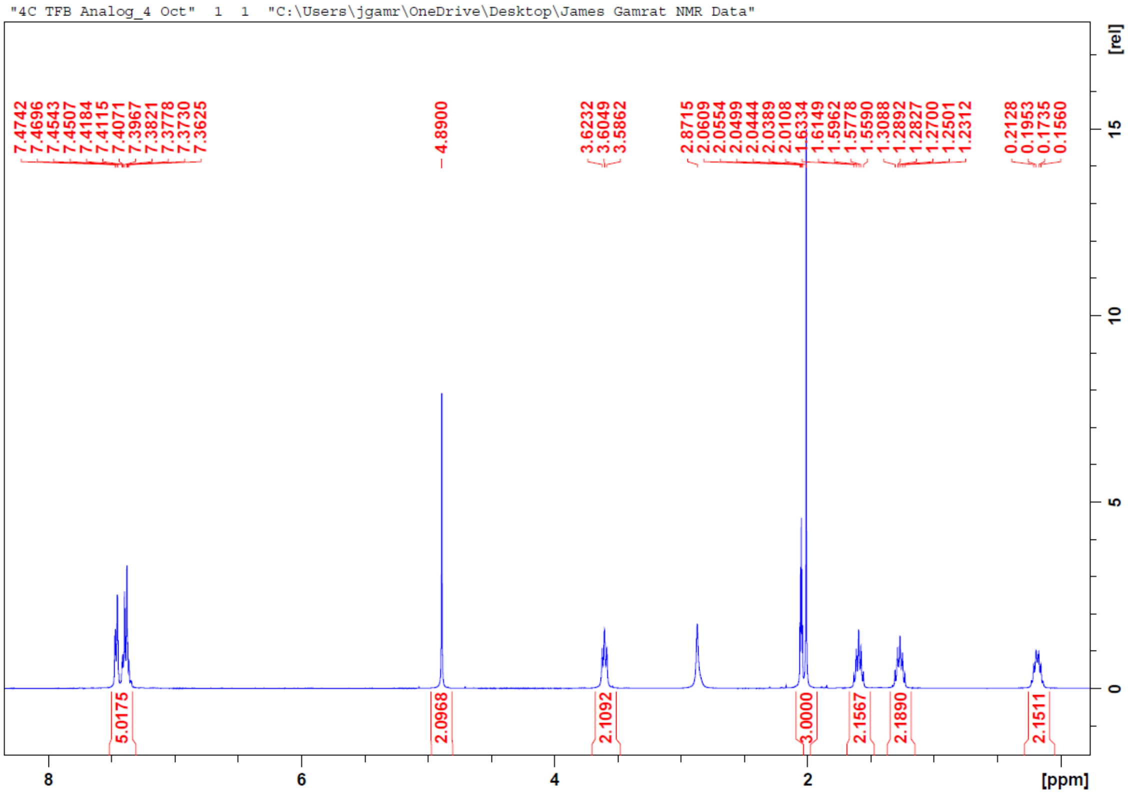

Figure S38. ^{13}C NMR spectrum of **3b** in Acetone-d_6.

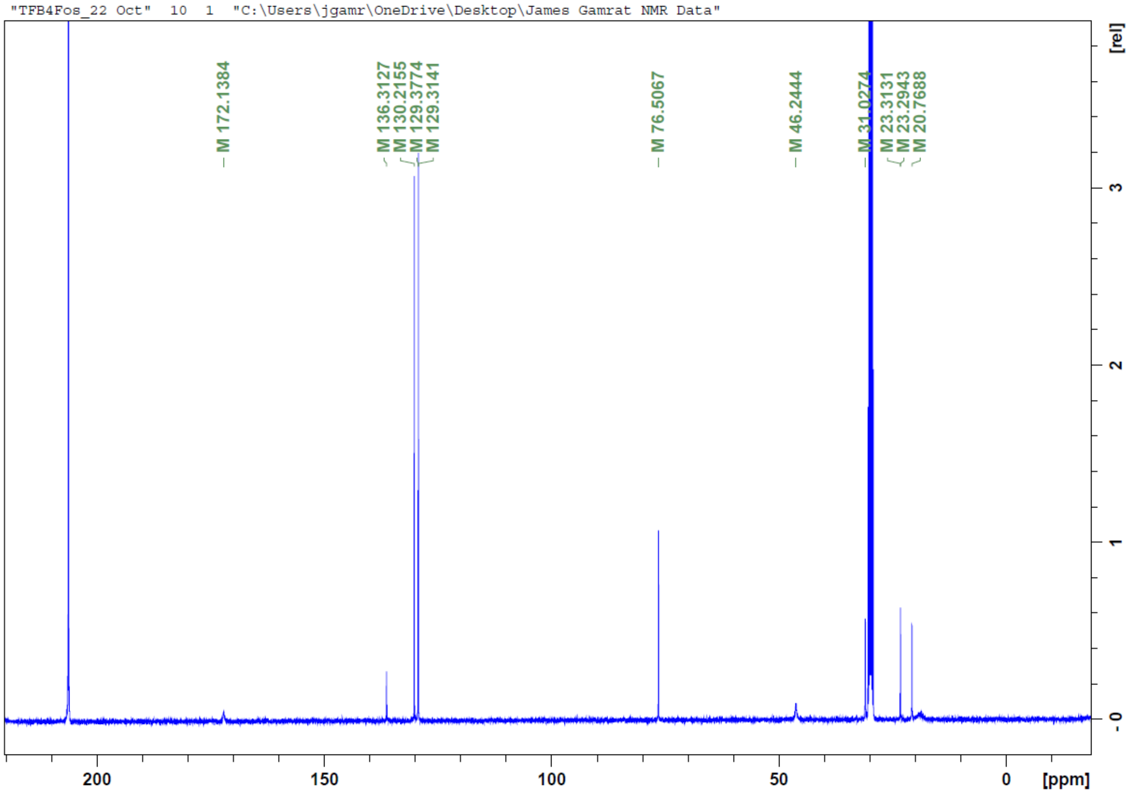

Figure S39. ^{1}H NMR spectrum of **4** in methanol-d$_4$.

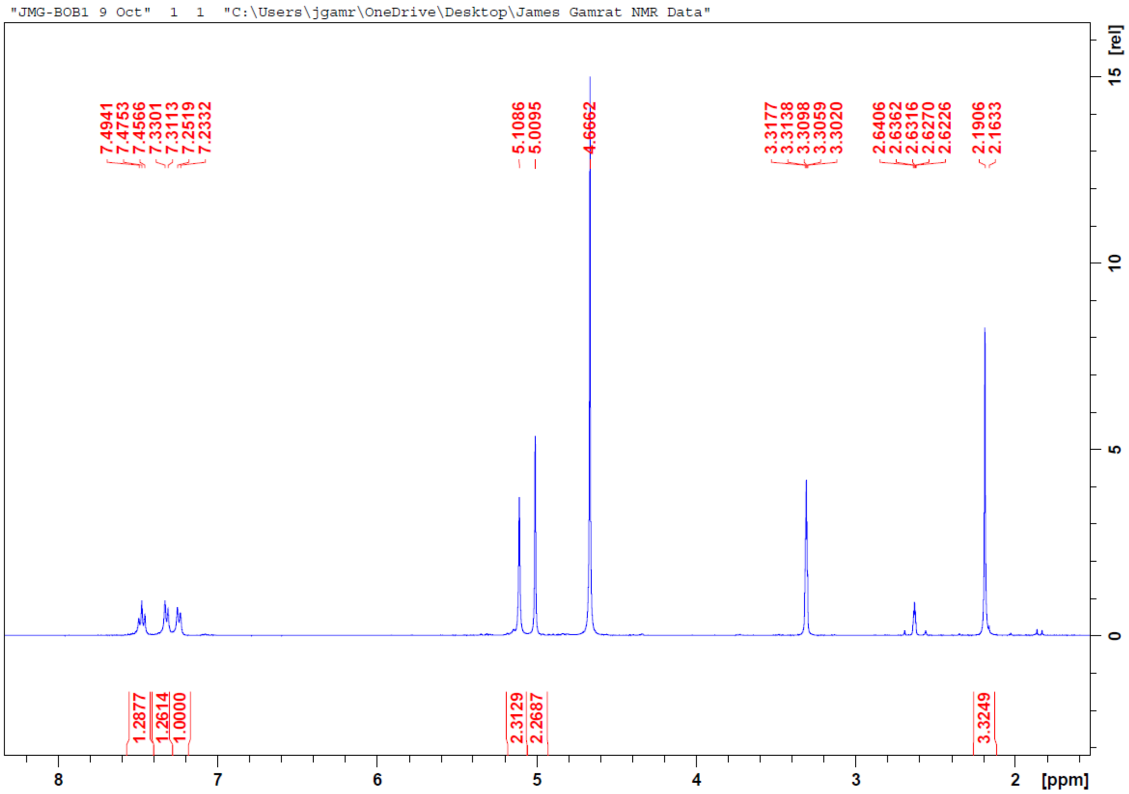

Figure S40. ^{13}C NMR spectrum of **4** in methanol-d$_4$.

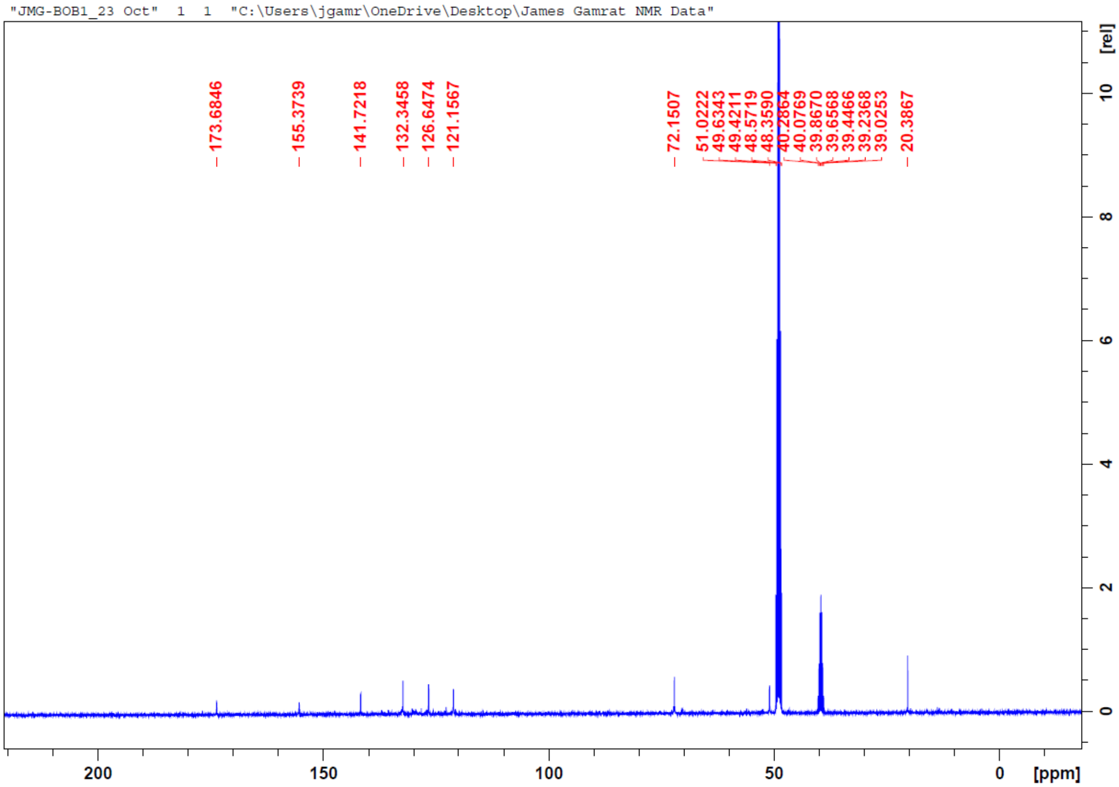

Figure S41. ^{11}B NMR spectrum of **4** in methanol-d_4.

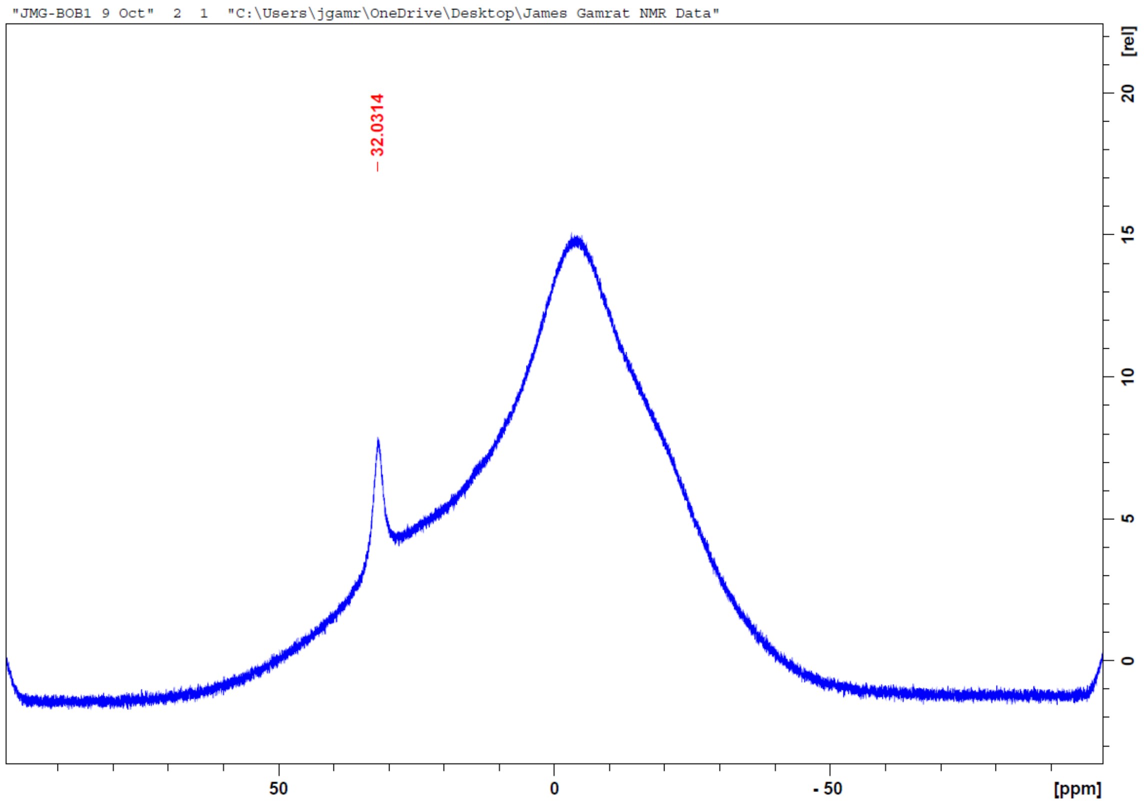

Figure S42. ^{1}H NMR spectrum of **5** in methanol-d$_4$.

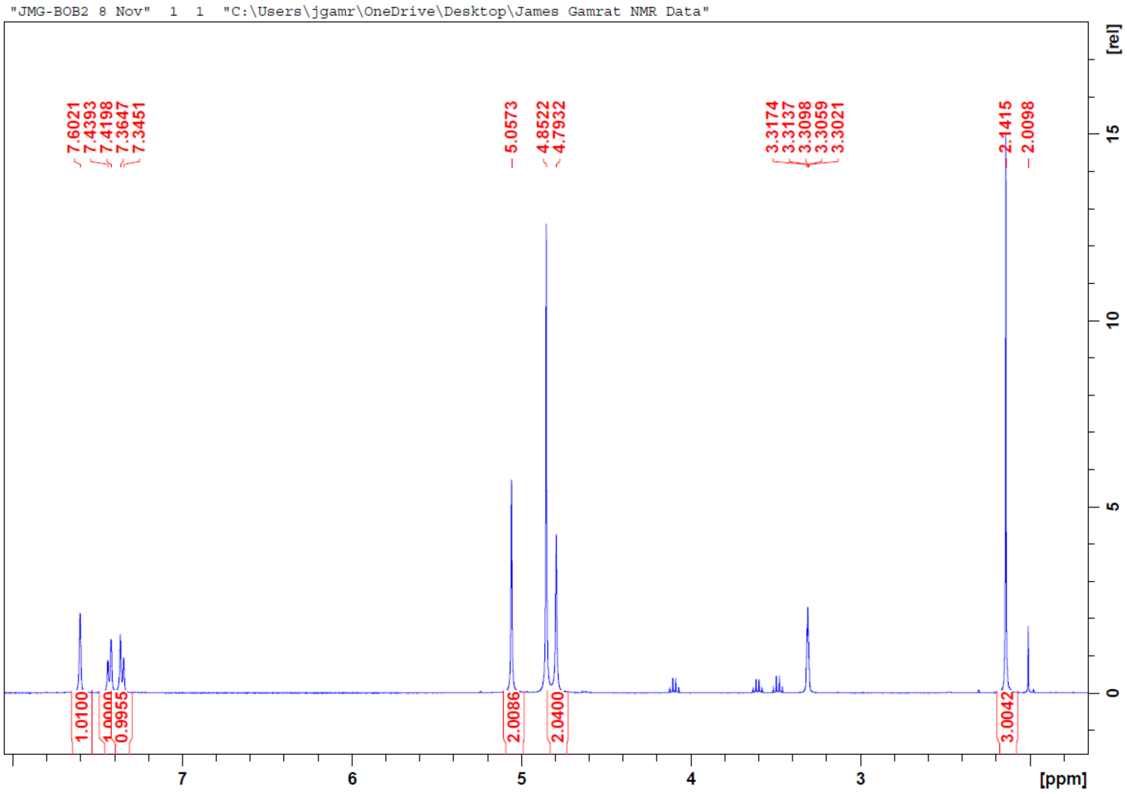

Figure S43. ^{13}C NMR spectrum of **5** in methanol-d$_4$.

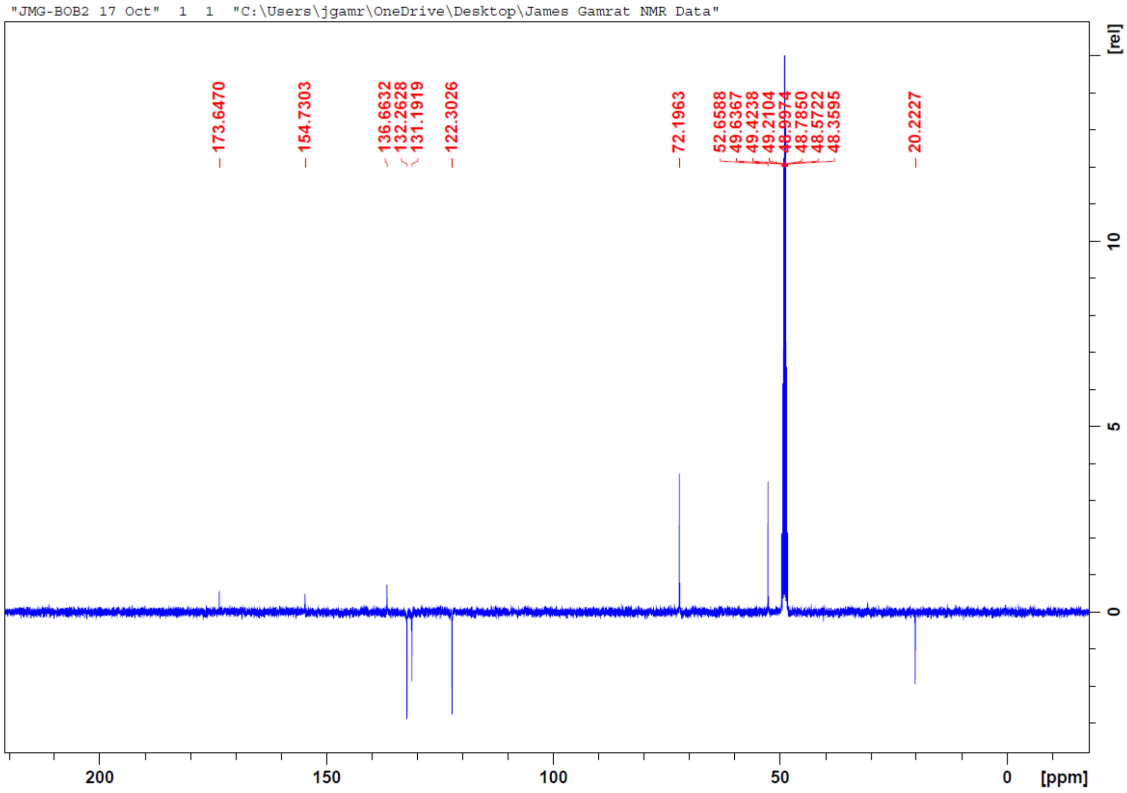

Figure S44. ^{11}B NMR spectrum of **5** in methanol-d$_4$.

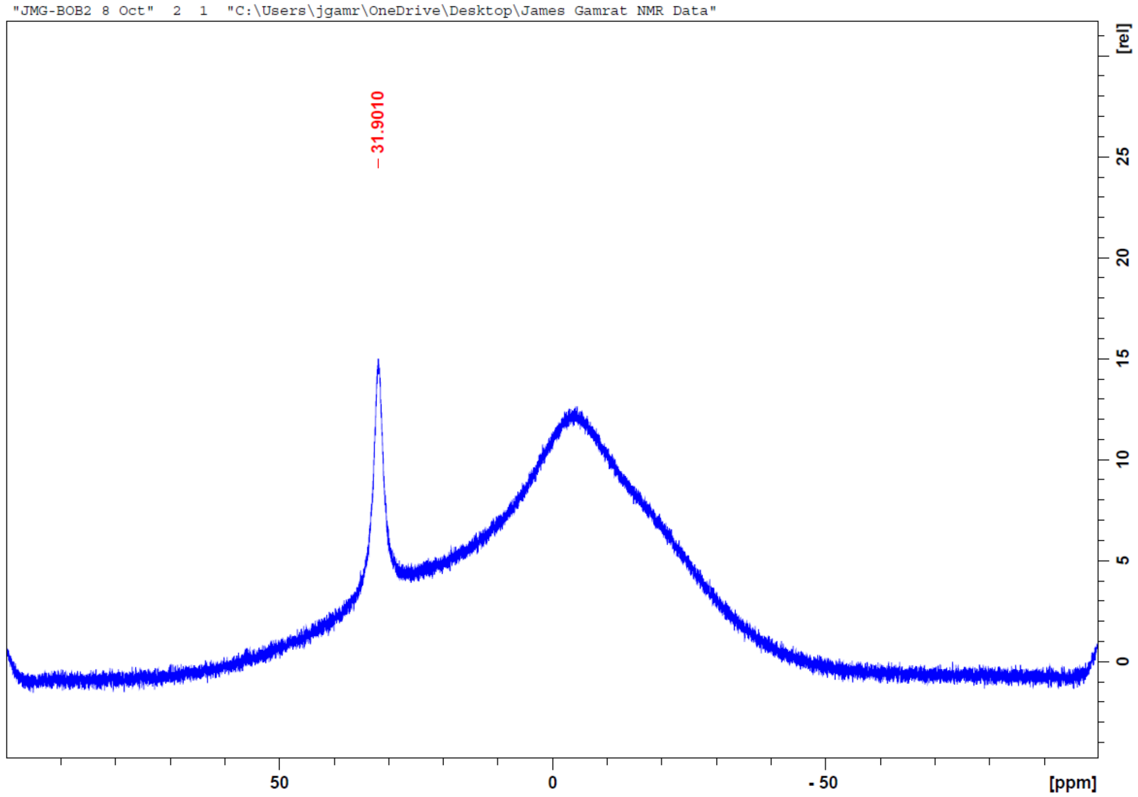

Figure S45. 2-D ^{1}H-^{1}H COSY NMR spectrum of **5** in methanol-d_4.

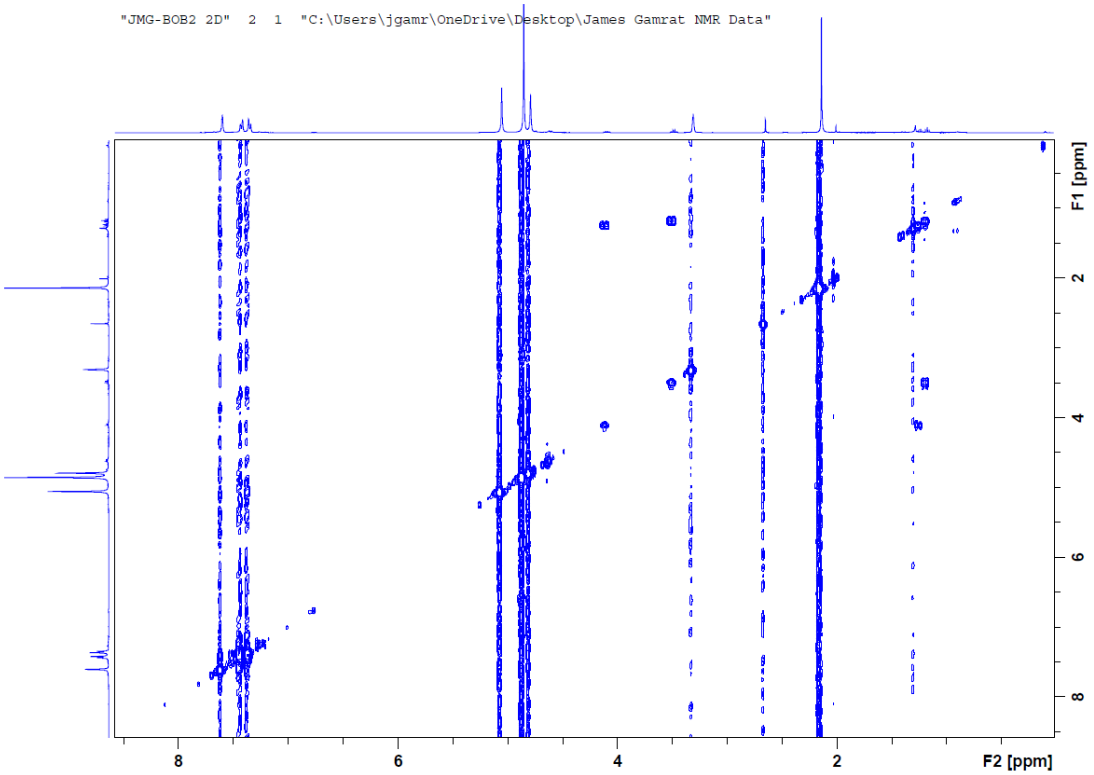

Figure S46. 2-D ^1H-^{13}C HMBC NMR spectrum of **5** in methanol-d_4.

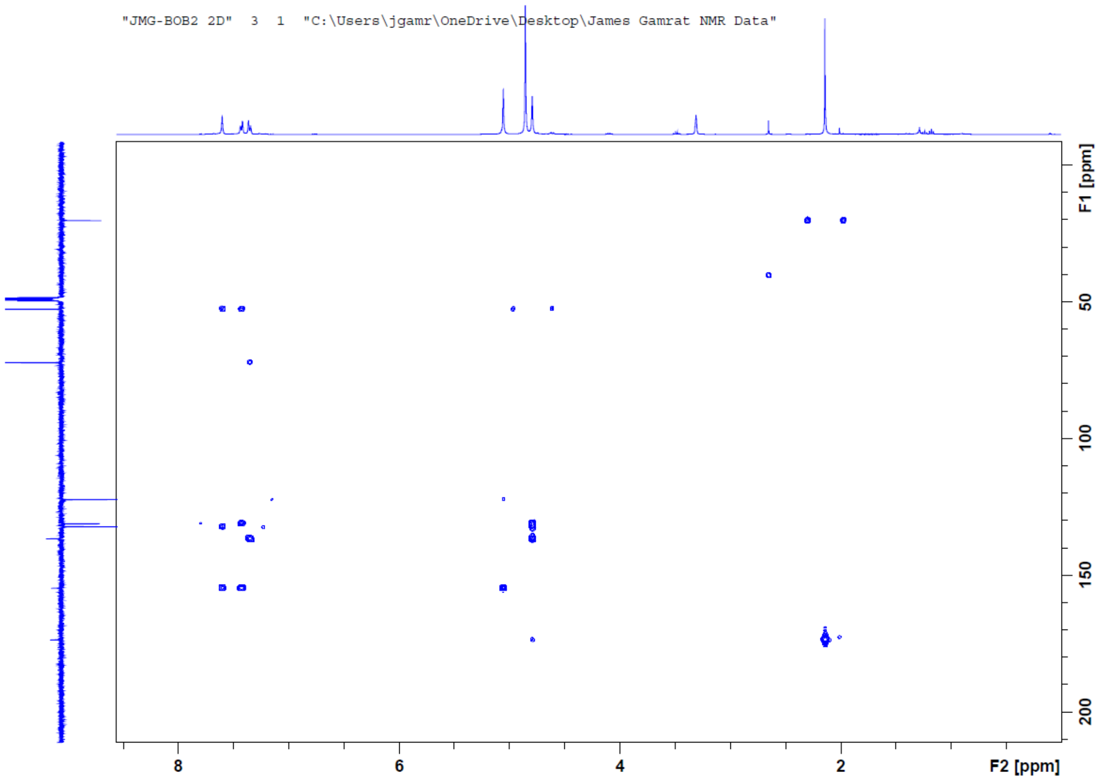

Figure S47. ^{1}H NMR spectrum of **6** in methanol-d4.

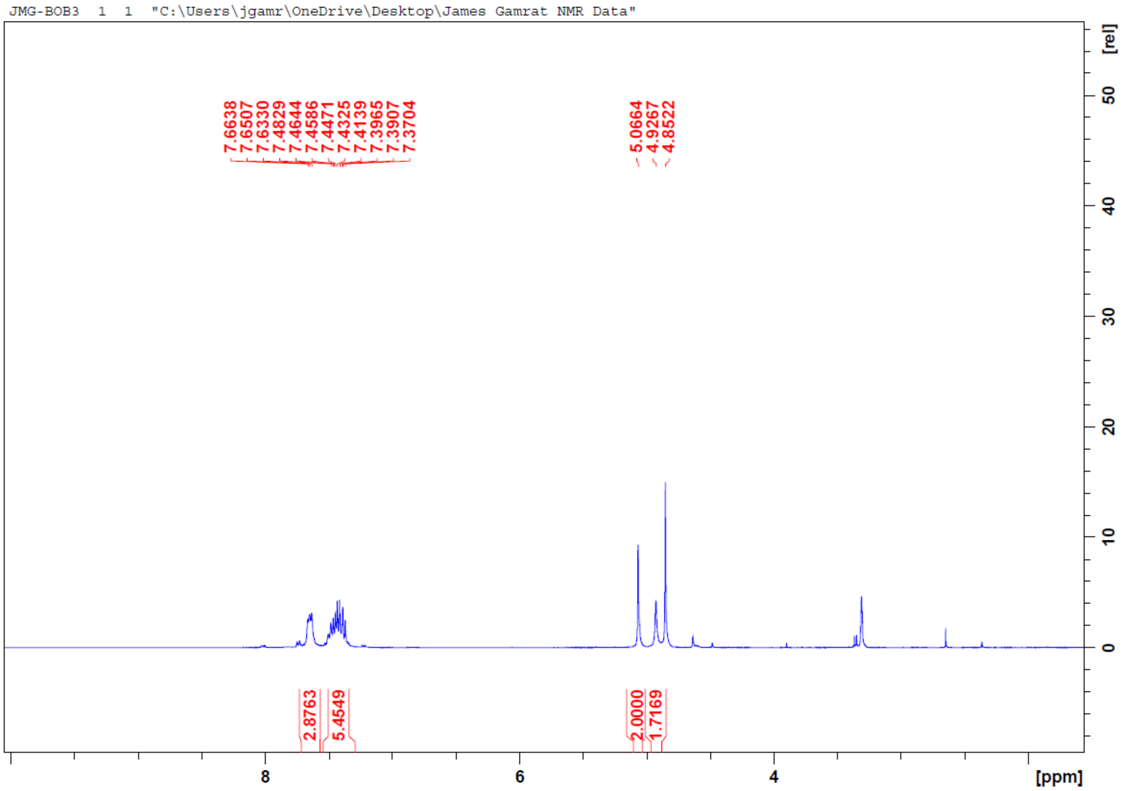

Figure S48. ^{13}C NMR spectrum of **6** in methanol-d$_4$.

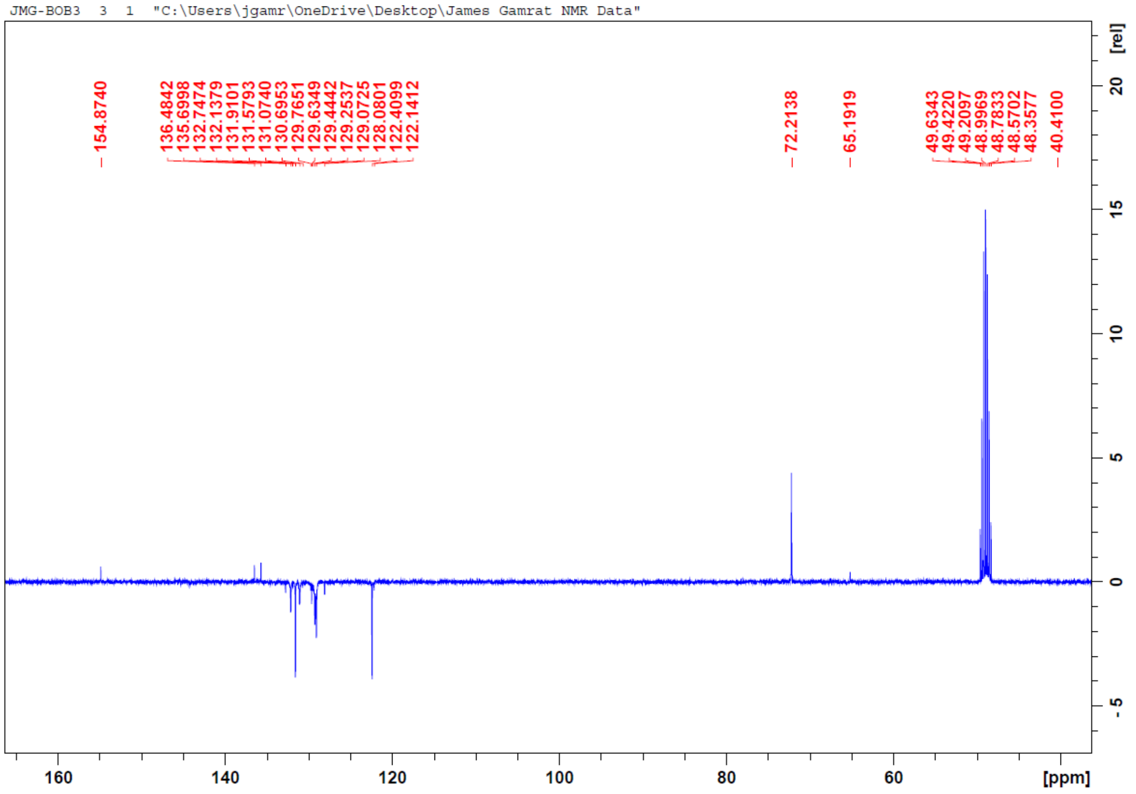

Figure S49. ^{11}B NMR spectrum of **6** in methanol-d_4.

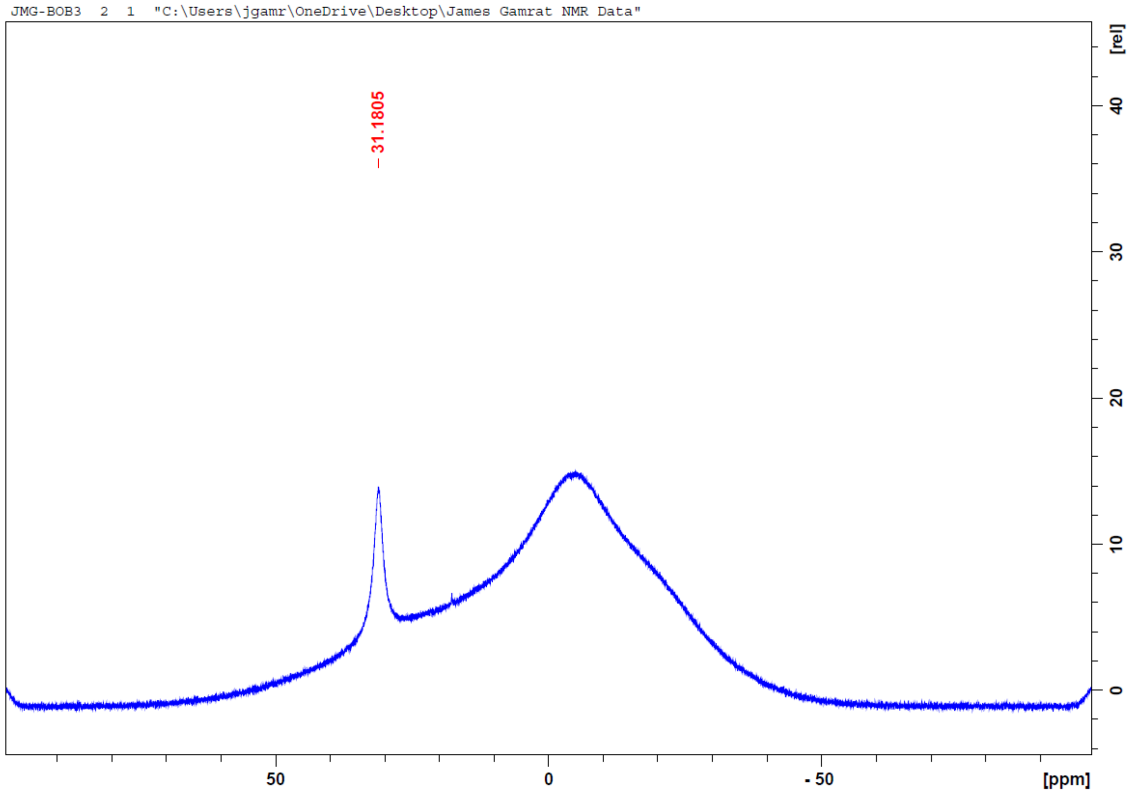

Figure S50. ^{1}H NMR spectrum of **7** in methanol-d_4.

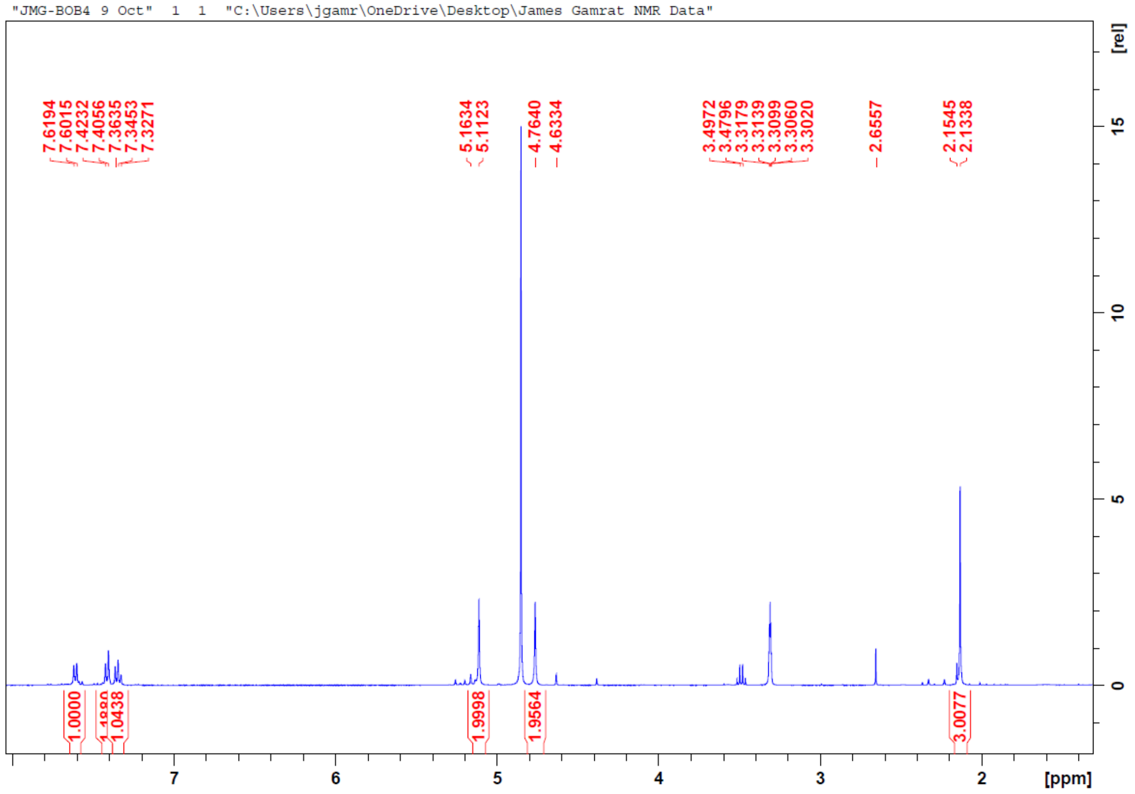

Figure S51. ^{13}C NMR spectrum of **7** in methanol-d$_4$.

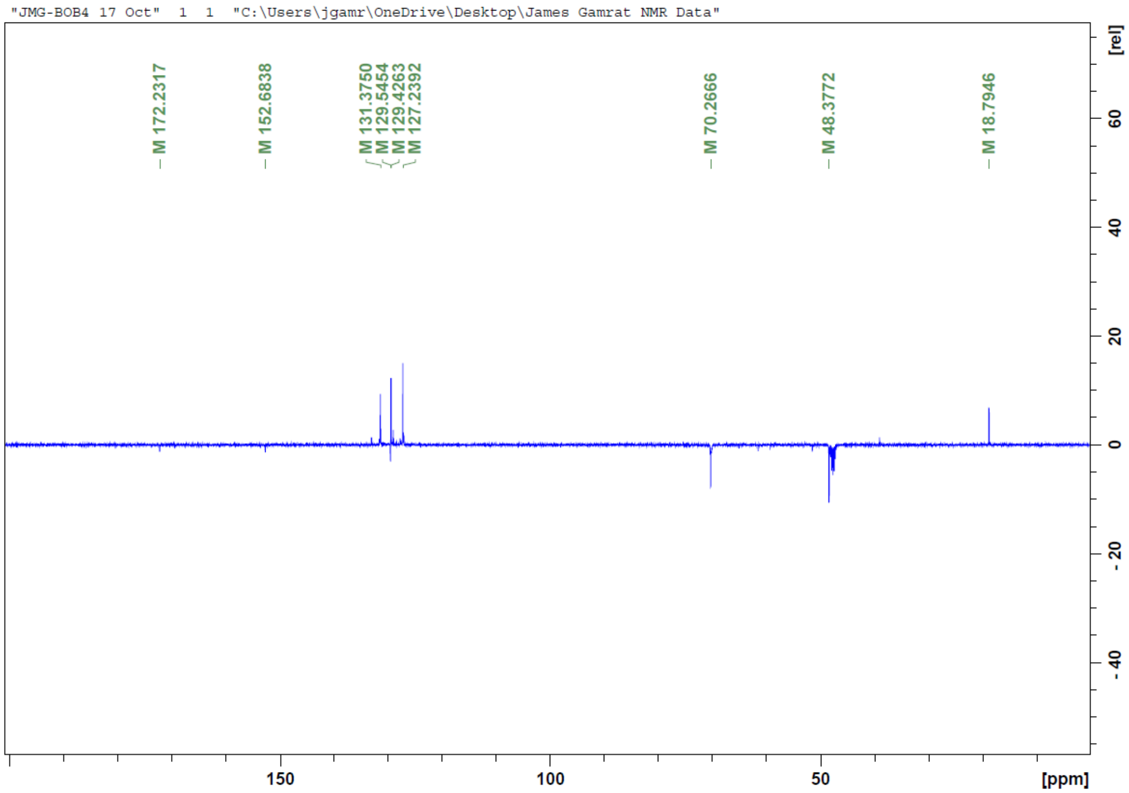

Figure S52. ^{11}B NMR spectrum of **7** in methanol-d$_4$.

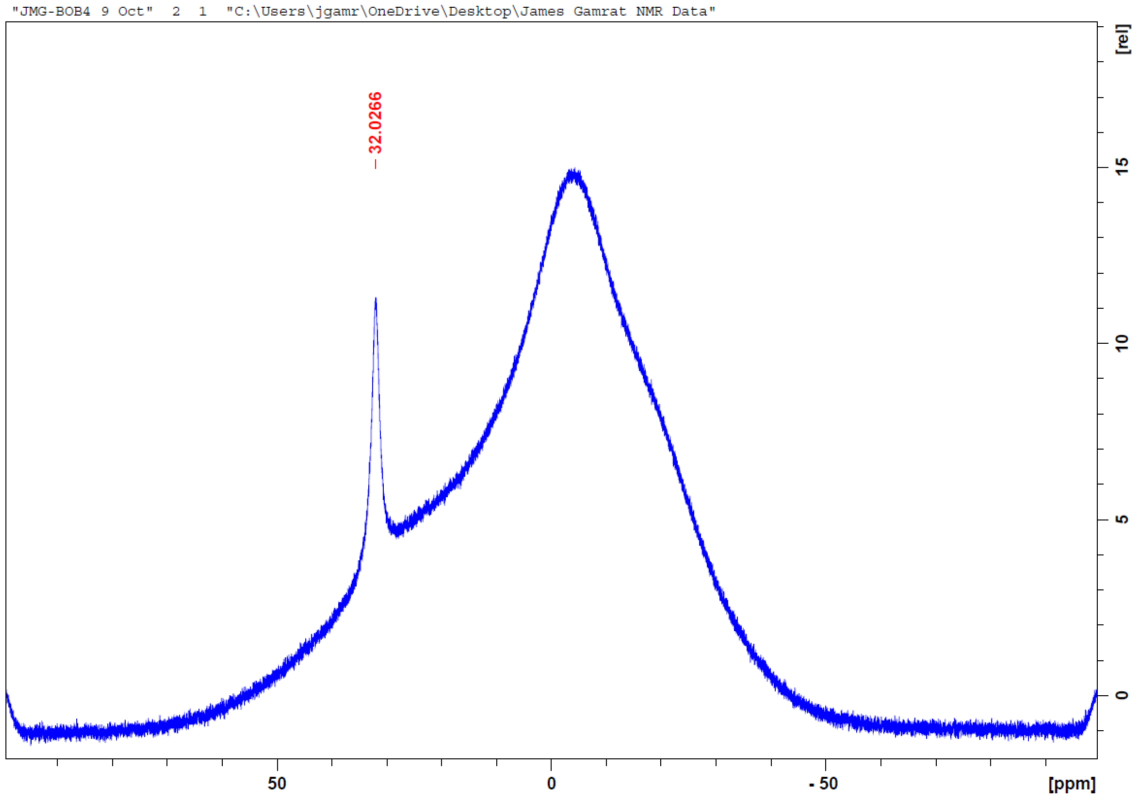

Figure S53. ^{1}H NMR spectrum of **8** in methanol-d$_4$.

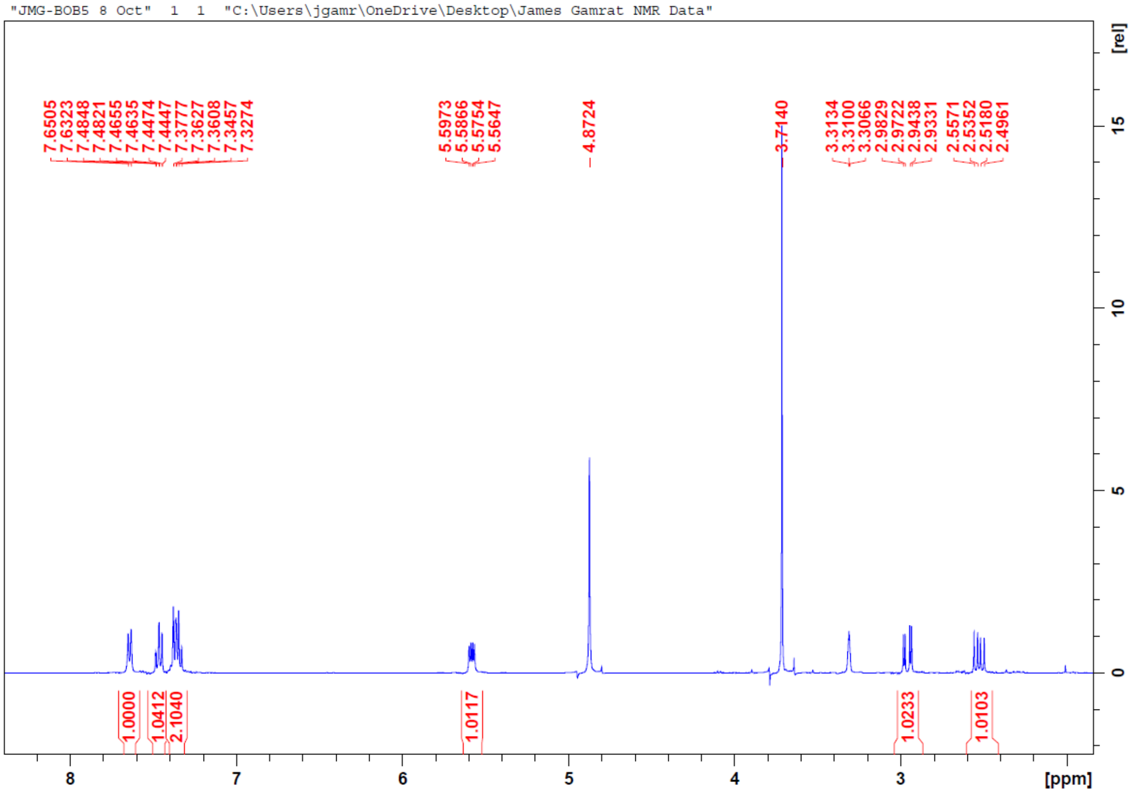

Figure S54. ^{13}C NMR spectrum of **8** in methanol-d_4.

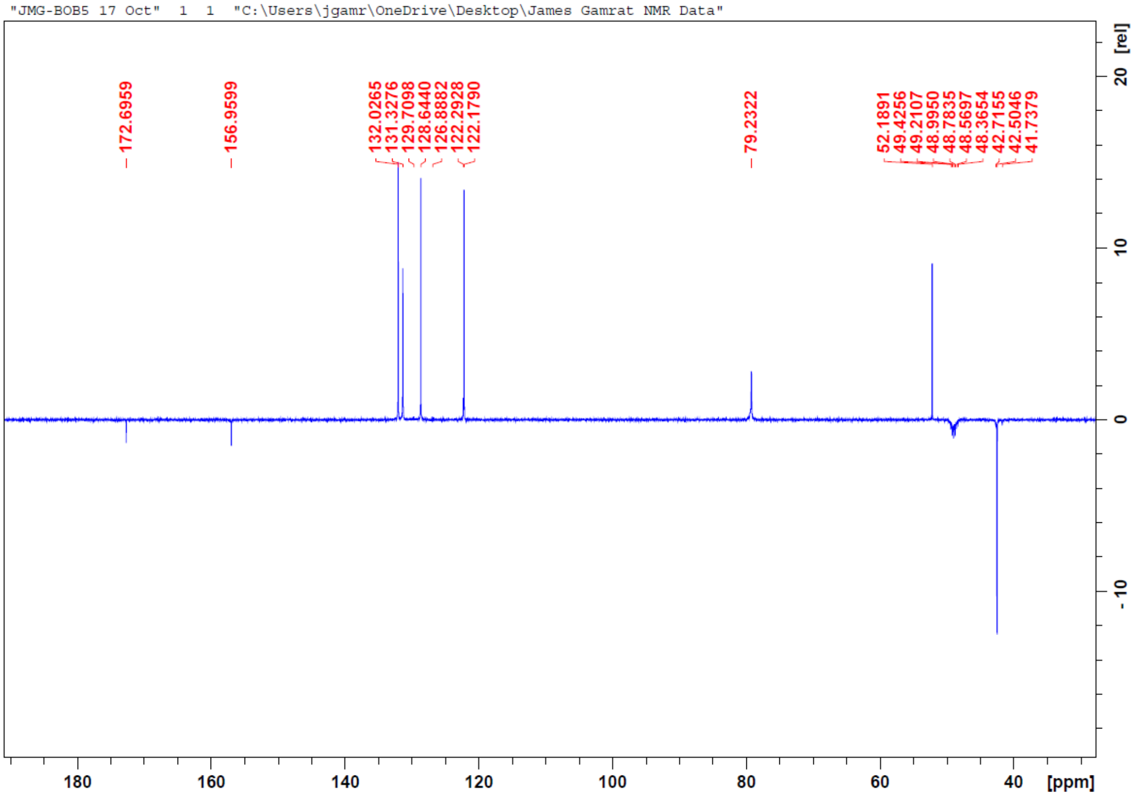

Figure S55. ^{11}B NMR spectrum of **8** in methanol-d_4.

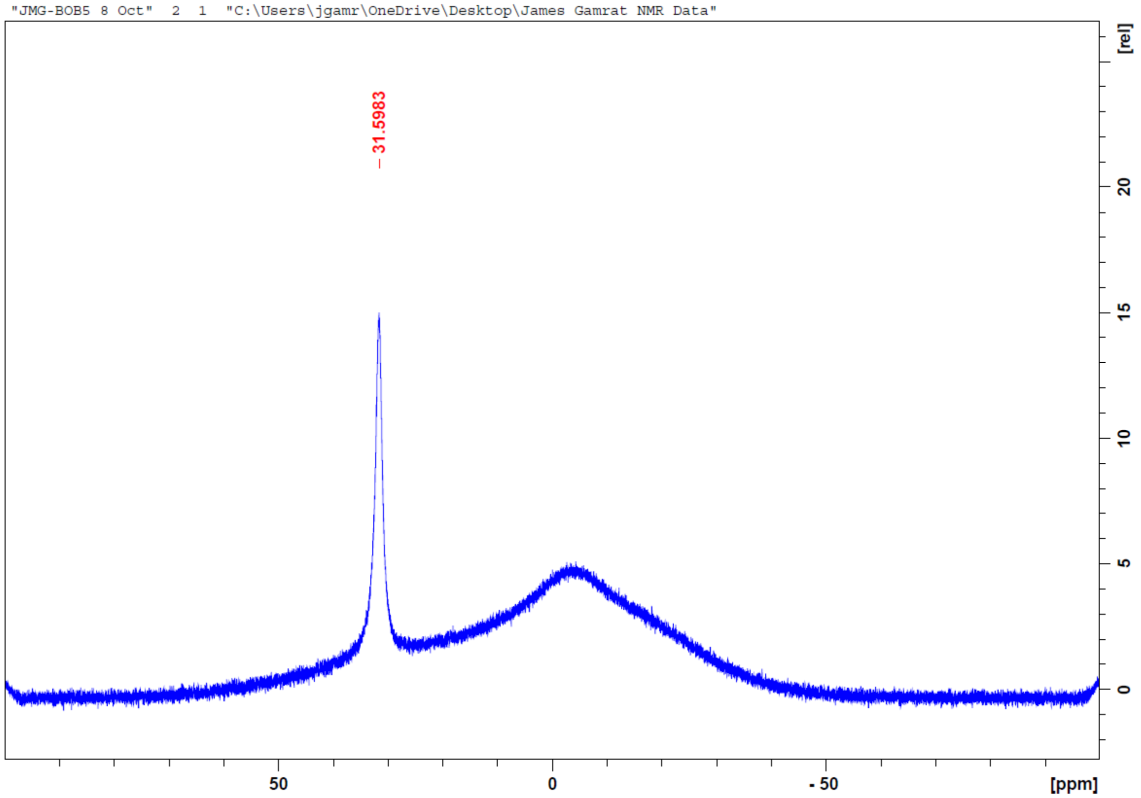

Figure S56. ^{1}H NMR spectrum of **9** in methanol-d$_4$.

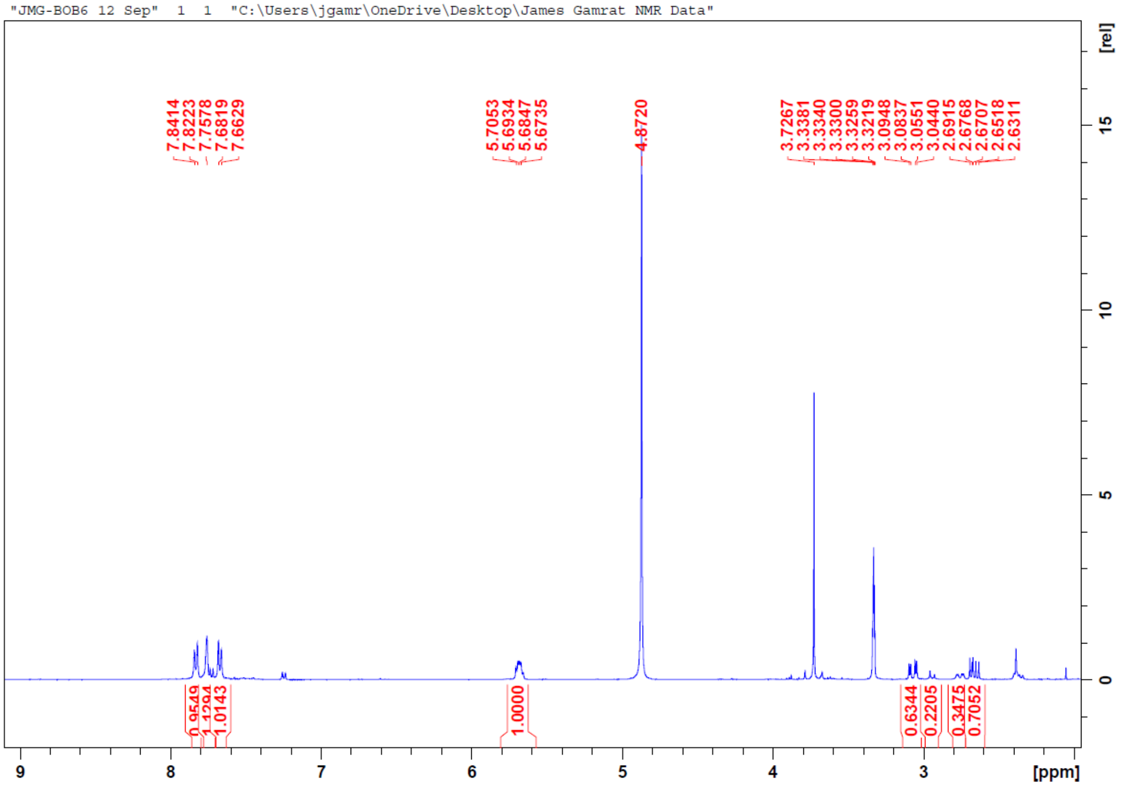

Figure S57. ^{13}C NMR spectrum of **9** in methanol-d_4.

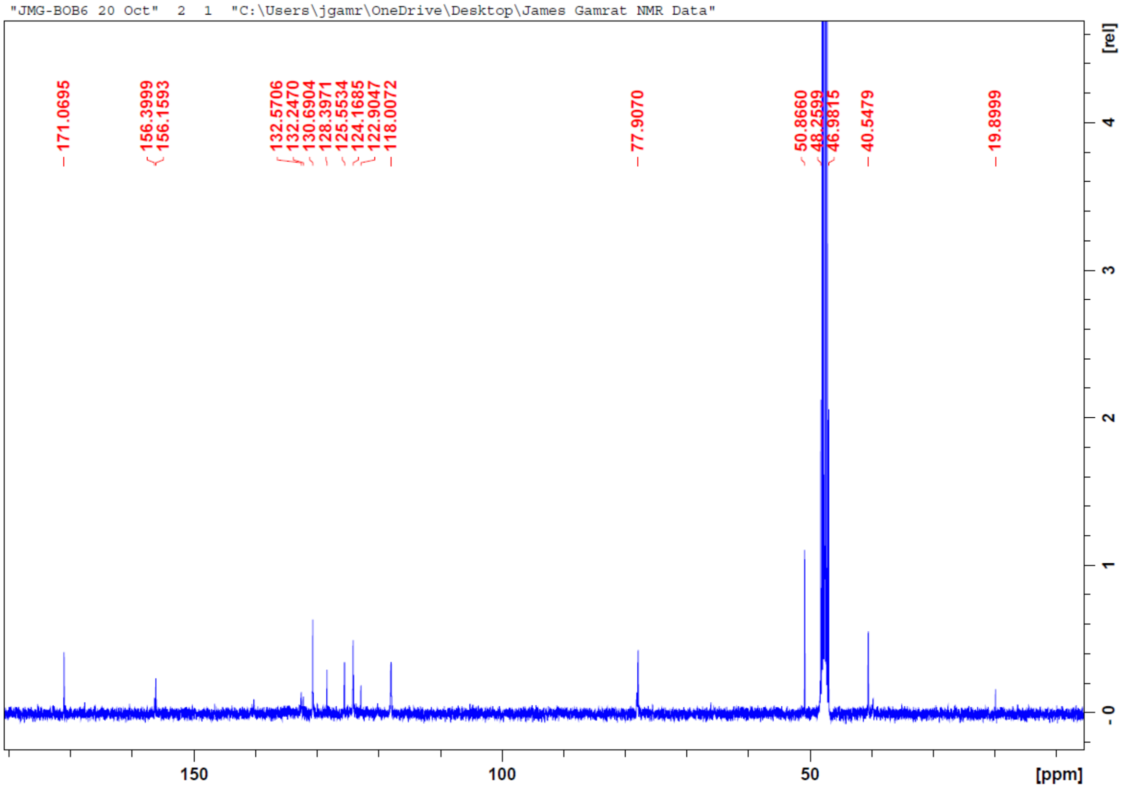

Figure S58. ^{11}B NMR spectrum of **9** in methanol-d$_4$.

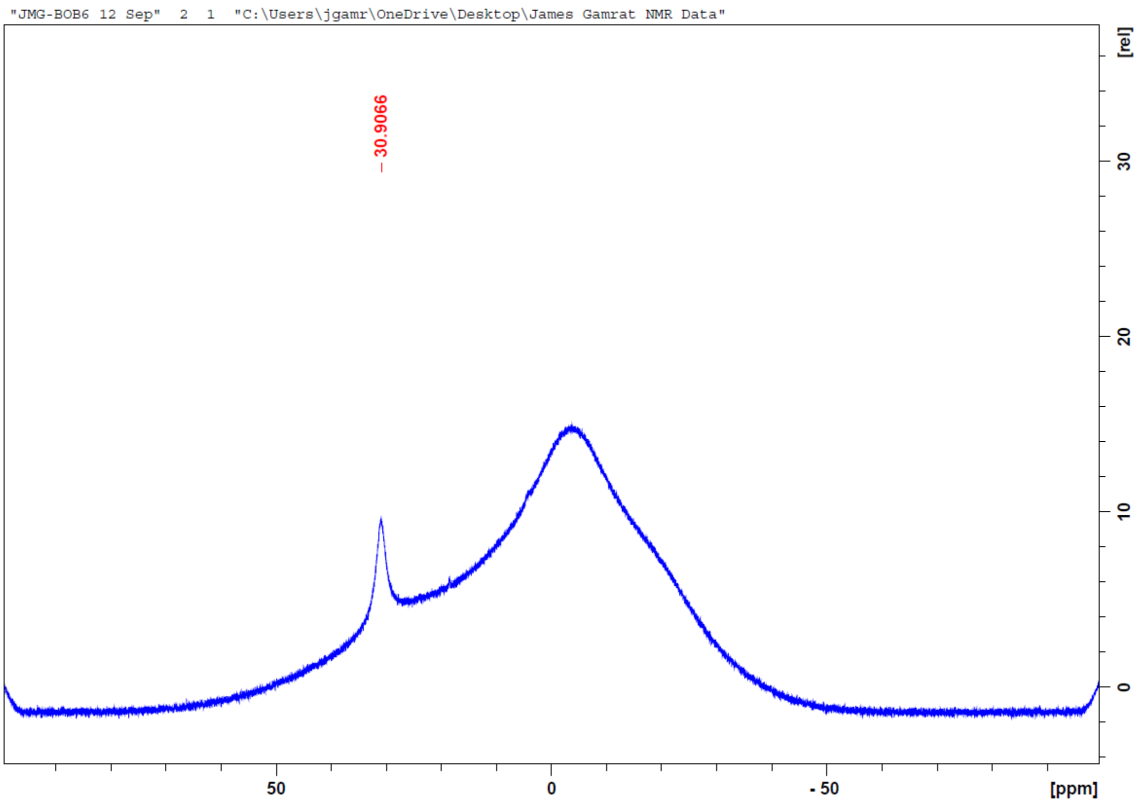

Figure S59. ^{1}H NMR spectrum of **10** in methanol-d4.

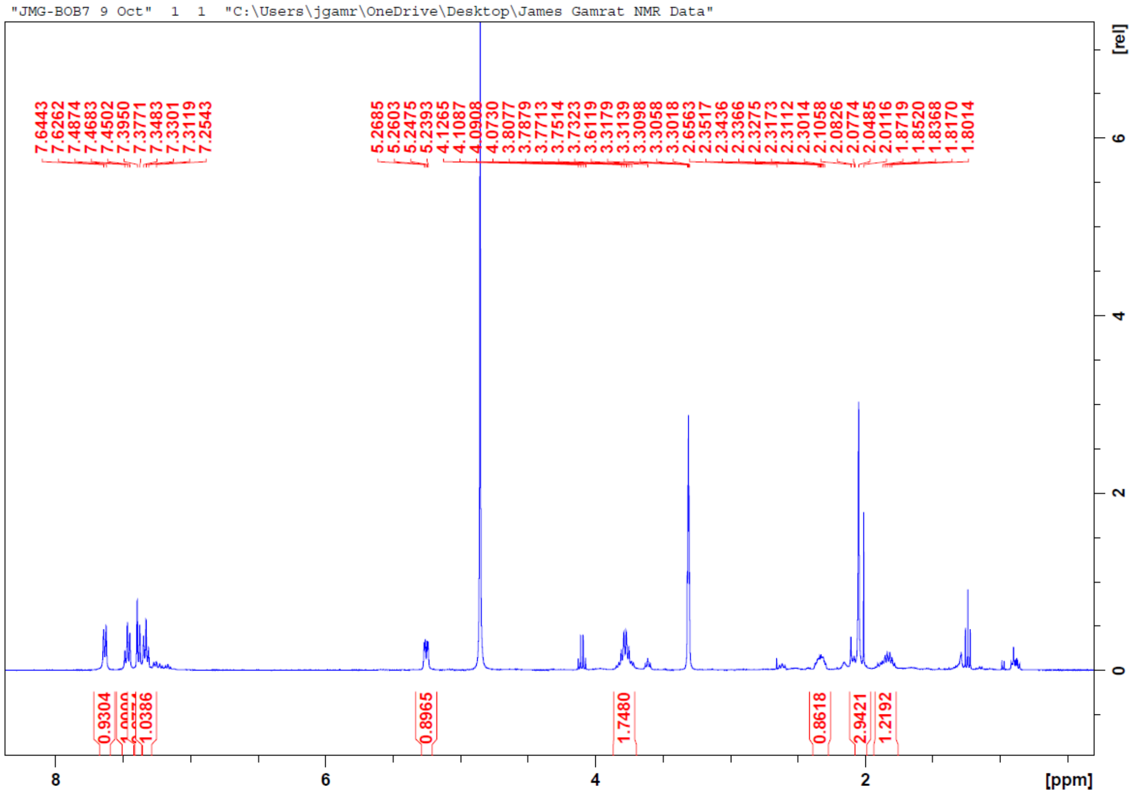

Figure S60. ^{13}C NMR spectrum of **10** in methanol-d$_4$.

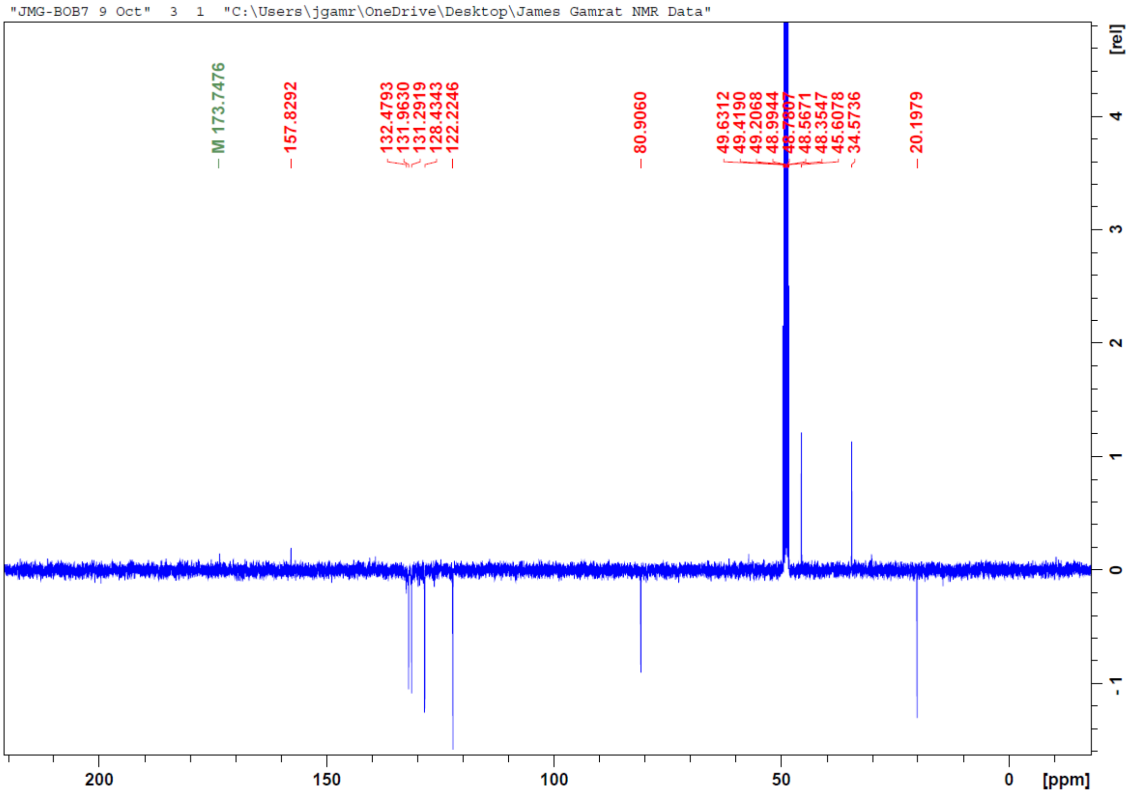

Figure S61. ^{11}B NMR spectrum of **10** in methanol-d_4.

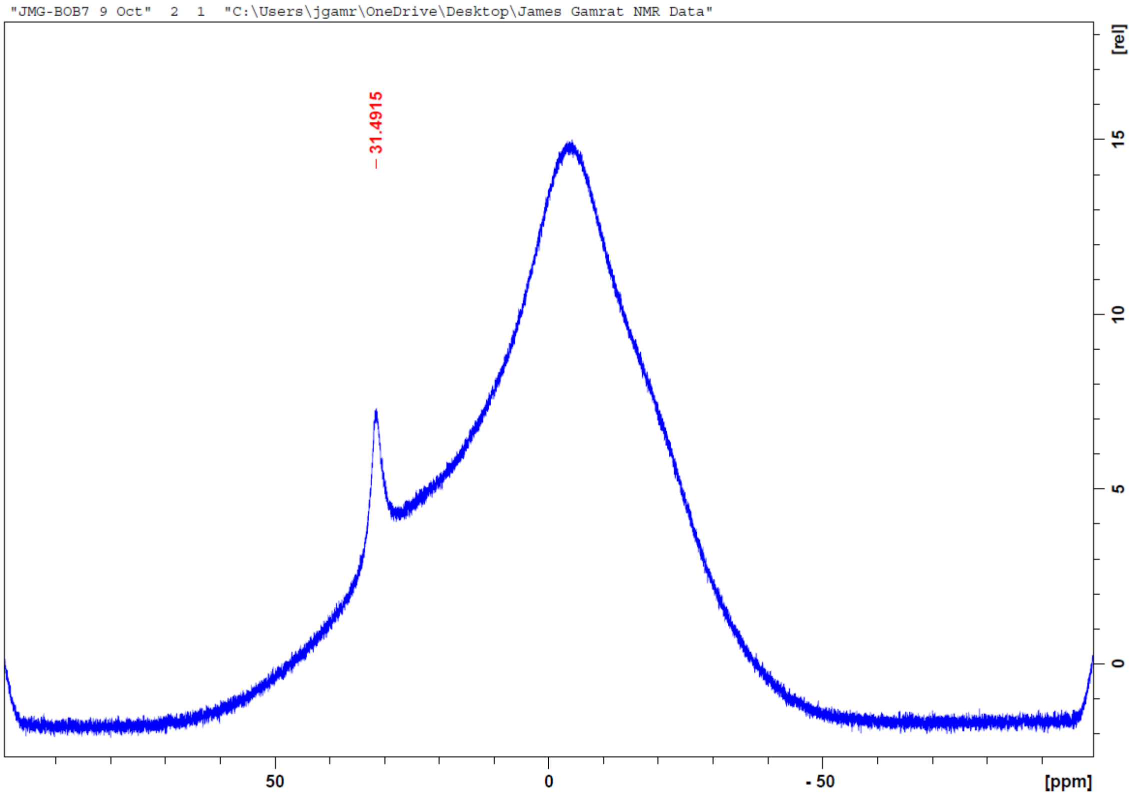

Purification and Kinetic Characterization of *Ec*IspC

Figure S62. SDS-PAGE Gel of *Ec*IspC purification.

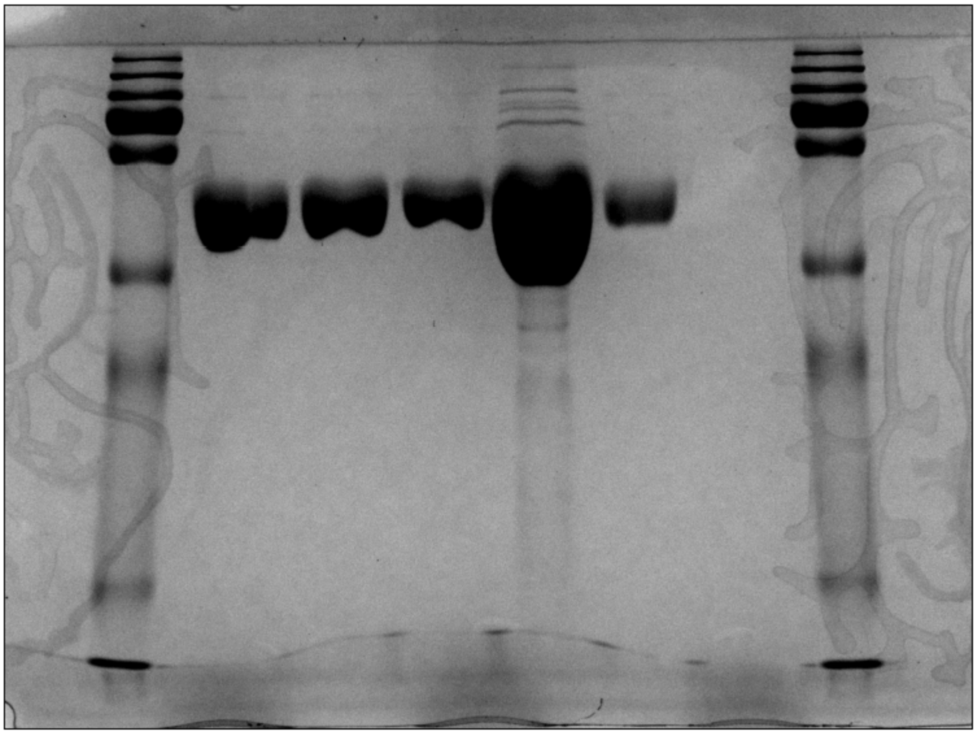

Figure S63. Michaelis-Menten plot of *Ec*IspC kinetic characterization.

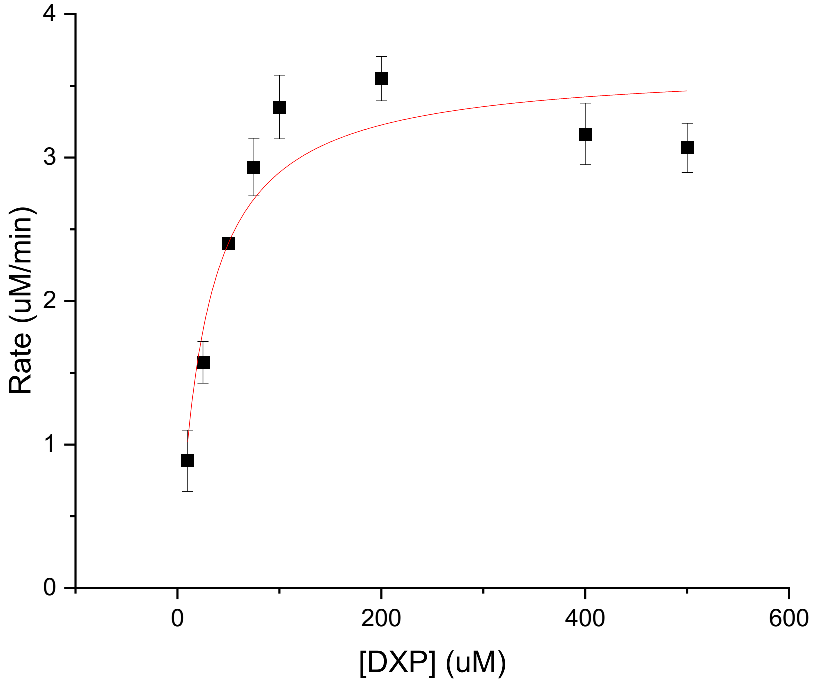

Table S1. Screening and microdilution assays of compound library against *E. coli* WT and *E. coli* ΔGlpT.

Compound	*E. coli* WT[a] % inhibition at 100 μg/mL	MIC ± SEM (μg/mL)	
		E. coli WT[a]	*E. coli* ΔGlpT[b]
**Fos**	N.D.	1.0 ± 0.1	> 400
**1a**	21	> 100	N.D.
**1b**	20	> 100	N.D.
**1c**	17	> 100	N.D.
**2a**	15	> 100	N.D.
**2b**	11	> 100	N.D.
**2c**	8	> 100	N.D.
**3a**	12	> 100	N.D.
**3b**	3	> 100	N.D.
**4**	16	> 100	N.D.
**5**	97	31 ± 2	30 ± 2
**6**	44	> 100	N.D.
**7**	25	> 100	N.D.
**8**	17	> 100	N.D.
**9**	13	> 100	N.D.
**10**	8	> 100	N.D.

[a]BW25113; [b]JW2234-2; N.D. = Not Determined

Table S2. *In vitro* Inhibition assay against *Ec*IspC.

Compound	% Inhibition at 100 μM	IC_{50} (μM ± SEM)
**Fos**	N.D.	0.025 ± 0.004
**1a**	<1	> 100
**1b**	<1	> 100
**1c**	<1	> 100
**2a**	<1	> 100
**2b**	<1	> 100
**2c**	<1	> 100
**3a**	<1	> 100
**3b**	<1	> 100
**4**	<1	> 100
**5**	<1	> 100
**6**	<1	> 100
**7**	3	> 100
**8**	<1	> 100
**9**	2	> 100
**10**	<1	> 100

N.D. = Not Determined

Minimum inhibitory concentration curves of active compounds.

Figure S64. MIC curve of **5** against *E. coli* WT.

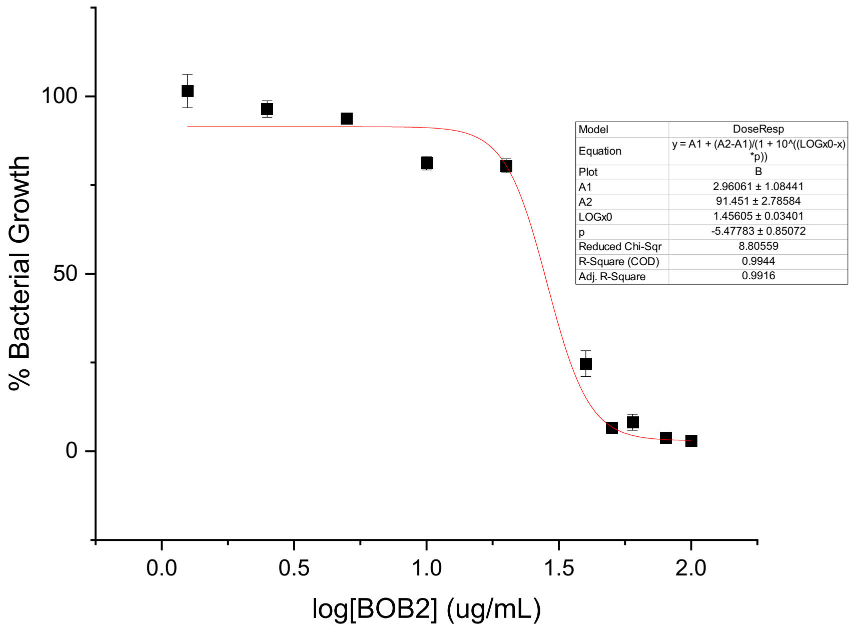

Figure S65. MIC curve of **5** against *E. coli* ΔGlpT.

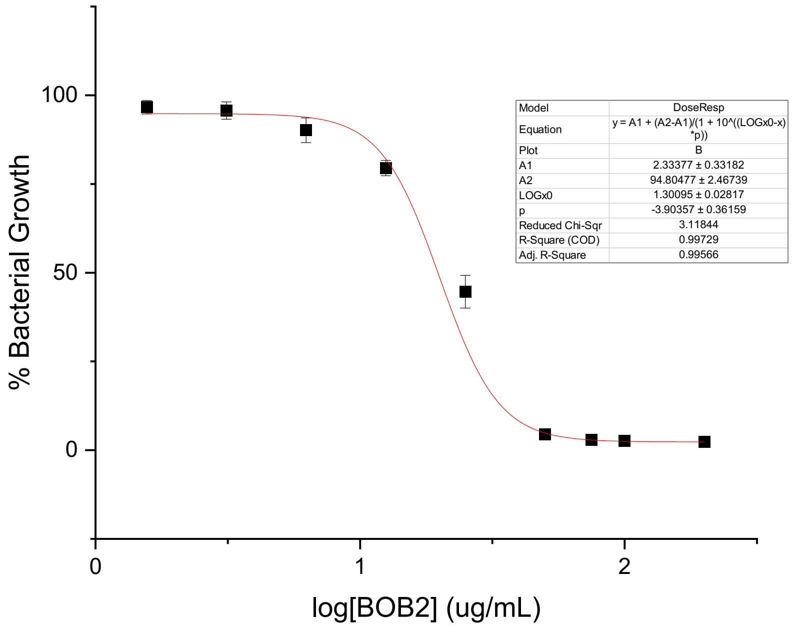

Figure S66. IPP rescue assay with **Fos** against *E. coli* WT.

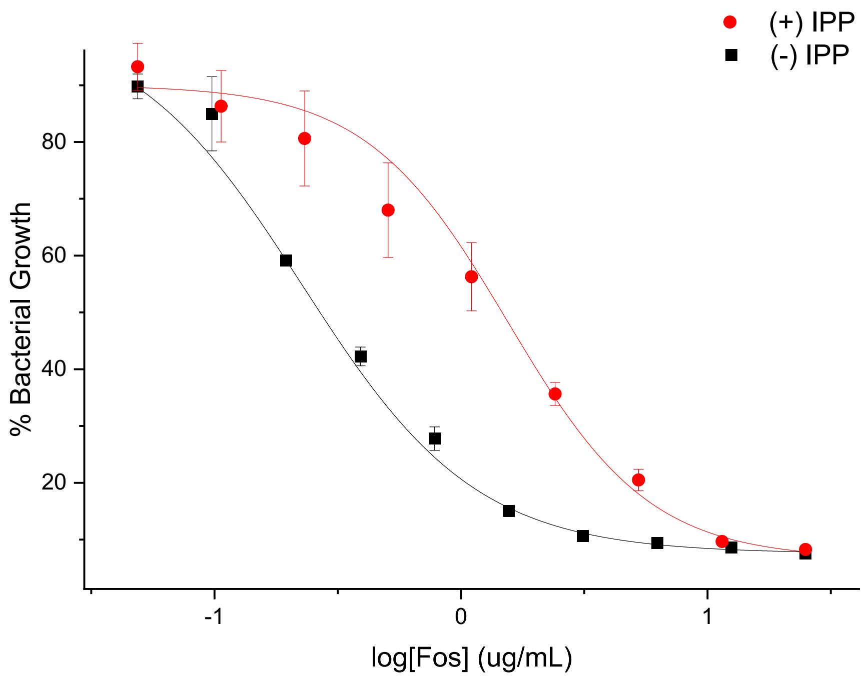

Figure S67. IPP rescue assay with **5** against *E. coli* WT.

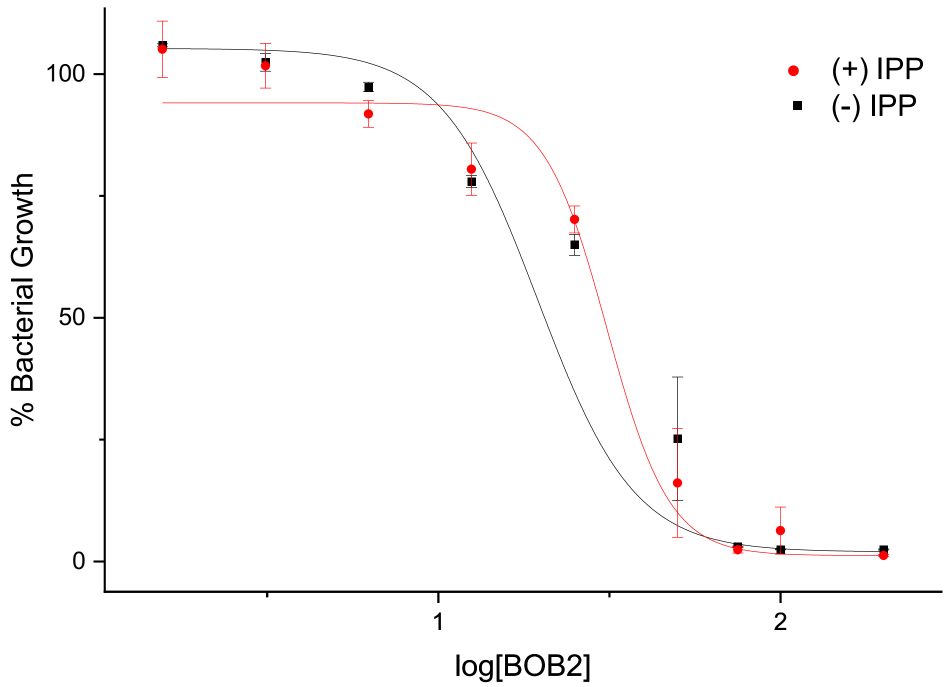

Figure S68. MIC curve of **5** against *S. aureus* Wichita.

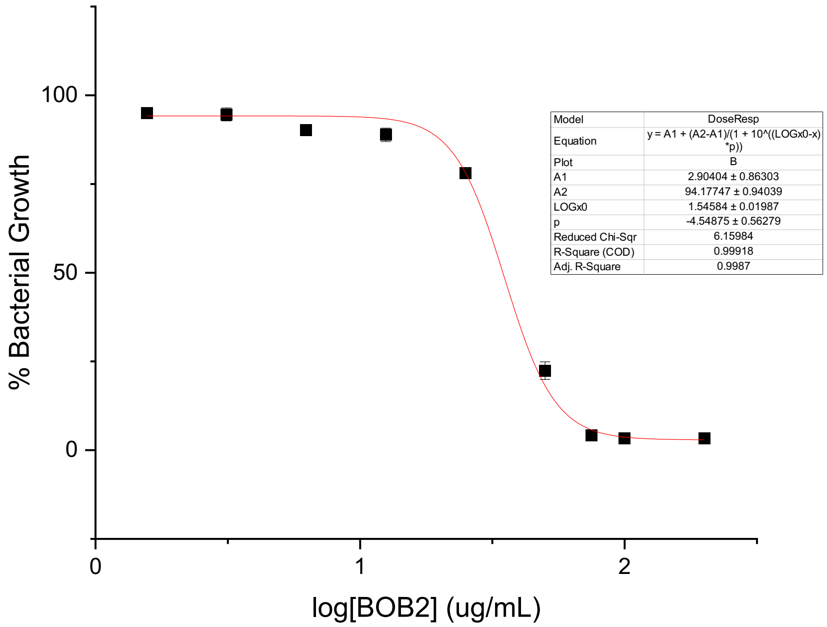

Figure S69. MIC curve of **6** against *S. aureus* Wichita.

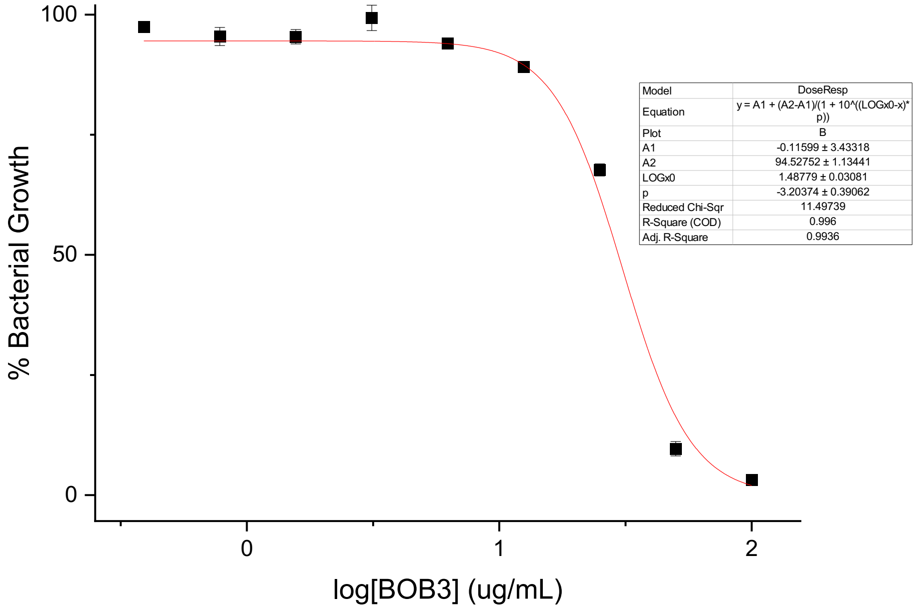

Figure S70. MIC curve of **5** against *S. aureus* ATCC43300.

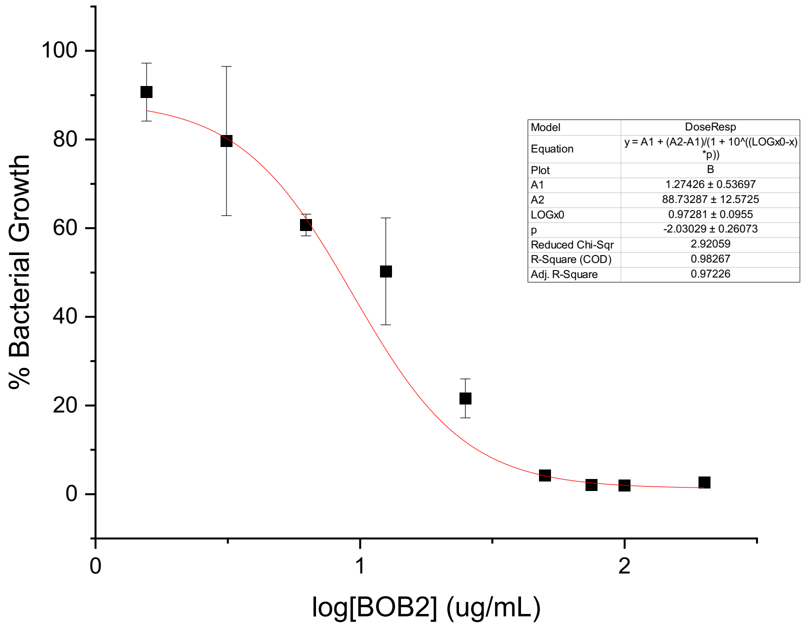

Figure S71. MIC curve of **6** against *S. aureus* ATCC43300.

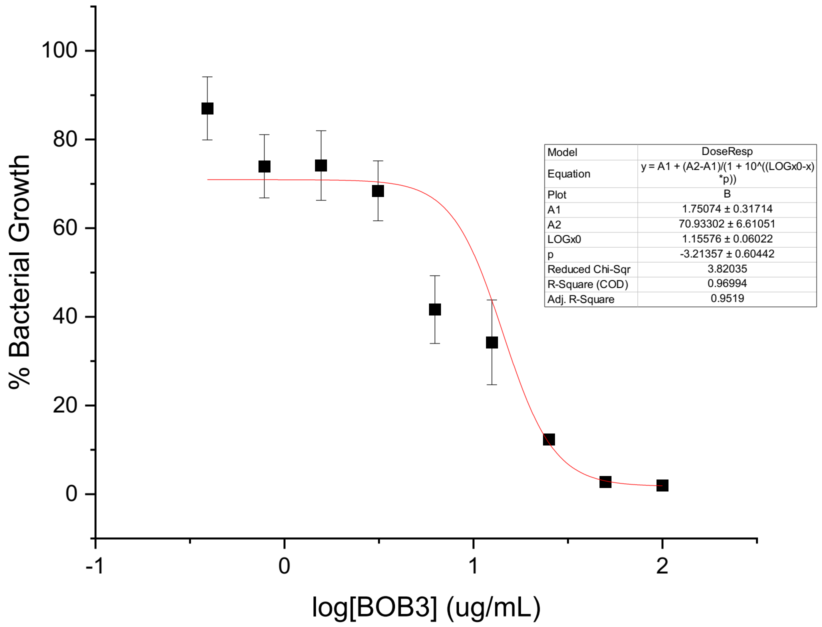

Data Tables from CO-ADD

Primary Screening Results: Tables S3-6

Table S3: Summary for Compounds **4** and **10**

CompoundID	CompoundName	ProjectID	RunID	Sel	Act	Sa	Ec	Kp	Pa	Ab	Ca	Cn	Conc
C0100586	4	P0761	PSR00168	0	0	75	3.2	3.23	6.37	20.19	12.17	22.31	32 ug/mL
C0100587	10	P0761	PSR00168	0	0	14.73	3.04	0.46	15.62	8.62	7.19	10.65	32 ug/mL

Table S4: Details for Compounds **4** and **10**

CompoundID	CompoundName	ProjectID	RunID	Sel	Act
C0100586	4	P0761	PSR00168	0	0
C0100587	10	P0761	PSR00168	0	0

CompoundID	CompoundName	ProjectID	RunID	Act_GP	Sa_Inhibition	Sa_ZScore	Sa_Act
C0100586	4	P0761	PSR00168	0	72.3; 75.0; PCT;	-6.7; -6.9	P; P
C0100587	10	P0761	PSR00168	0	14.7; 9.7; PCT;	-0.2; -0.4	I; I

CompoundID	CompoundName	ProjectID	RunID	Act_GN	Ec_Inhibition	Ec_ZScore	Ec_Act	Kp_Inhibition	Kp_ZScore	Kp_Act	Pa_Inhibition	Pa_ZScore	Pa_Act	Ab_Inhibition	Ab_ZScore	Ab_Act
C0100586	4	P0761	PSR00168	0	0.5; 3.2; PCT;	-0.4; 0.1	I; I	-10.4; 3.2; PCT;	0.7; 1.9	I; I	4.9; 6.4; PCT;	-1.0; 0.7	I; I	15.4; 20.2; PCT;	-0.5; -1.6	I; I
C0100587	10	P0761	PSR00168	0	1.3; 3.0; PCT;	-0.6; 0.1	I; I	-5.5; 0.5; PCT;	0.6; 1.6	I; I	-0.9; 15.6; PCT;	-1.0; 0.2	I; I	6.0; 8.6; PCT;	0.1; 1.1	I; I

CompoundID	CompoundName	ProjectID	RunID	Act_FG	Ca_Inhibition	Ca_ZScore	Ca_Act	Cn_Inhibition	Cn_ZScore	Cn_Act
C0100586	4	P0761	PSR00168	0	11.4; 12.2; PCT;	-1.5; -2.0	I; I	-5.5; 22.3; PCT;	-3.5; 0.7	I; I
C0100587	10	P0761	PSR00168	0	3.5; 7.2; PCT;	-1.0; 0.5	I; I	10.7; 8.1; PCT;	-1.1; -1.7	I; I

Table S5: Inhibition Data for Compounds **4** and **10**

CompoundID	CompoundName	ProjectID	OrgID	OrgCode	Organism	Strain	PSRunID	TestPlateID	TestWellID	Inhibition	Zscore	Conc	ConcUnit
C0100586	4	P0761	FG_001	Ca	Candida albicans	ATCC 90028	PSR00168	TP00701-18F	A02	12.17	-1.54	32.00	ug/mL
C0100586	4	P0761	FG_001	Ca	Candida albicans	ATCC 90028	PSR00168	TP00701-17F	A02	11.37	-1.96	32.00	ug/mL
C0100586	4	P0761	FG_002	Cn H99	Cryptococcus neoformans	ATCC 208821; H99	PSR00168	TP00701-19F	A02	-5.54	0.74	32.00	ug/mL
C0100586	4	P0761	FG_002	Cn H99	Cryptococcus neoformans	ATCC 208821; H99	PSR00168	TP00701-20F	A02	22.31	-3.49	32.00	ug/mL
C0100586	4	P0761	GP_020	Sa MRSa	Staphylococcus aureus	ATCC 43300; MRSA	PSR00168	TP00701-15V	A02	72.34	-6.87	32.00	ug/mL
C0100586	4	P0761	GP_020	Sa MRSa	Staphylococcus aureus	ATCC 43300; MRSA	PSR00168	TP00701-16V	A02	75.00	-6.65	32.00	ug/mL
C0100586	4	P0761	GN_001	Ec	Escherichia coli	ATCC 25922	PSR00168	TP00701-02C	A02	3.20	0.06	32.00	ug/mL
C0100586	4	P0761	GN_001	Ec	Escherichia coli	ATCC 25922	PSR00168	TP00701-01C	A02	0.52	-0.38	32.00	ug/mL
C0100586	4	P0761	GN_042	Pa	Pseudomonas aeruginosa	ATCC 27853	PSR00168	TP00701-08C	A02	6.37	0.67	32.00	ug/mL
C0100586	4	P0761	GN_042	Pa	Pseudomonas aeruginosa	ATCC 27853	PSR00168	TP00701-07C	A02	4.85	-0.96	32.00	ug/mL
C0100586	4	P0761	GN_034	Ab	Acinetobacter baumannii	ATCC 19606	PSR00168	TP00701-06C	A02	15.39	-0.54	32.00	ug/mL
C0100586	4	P0761	GN_034	Ab	Acinetobacter baumannii	ATCC 19606	PSR00168	TP00701-05C	A02	20.19	-1.62	32.00	ug/mL
C0100586	4	P0761	GN_003	Kp MDR	Klebsiella pneumoniae	ATCC 700603; MDR	PSR00168	TP00701-03C	A02	3.23	0.66	32.00	ug/mL
C0100586	4	P0761	GN_003	Kp MDR	Klebsiella pneumoniae	ATCC 700603; MDR	PSR00168	TP00701-04C	A02	-10.40	1.88	32.00	ug/mL
C0100587	10	P0761	FG_001	Ca	Candida albicans	ATCC 90028	PSR00168	TP00701-18F	A04	3.52	0.46	32.00	ug/mL
C0100587	10	P0761	FG_001	Ca	Candida albicans	ATCC 90028	PSR00168	TP00701-17F	A04	7.19	-1.02	32.00	ug/mL
C0100587	10	P0761	FG_002	Cn H99	Cryptococcus neoformans	ATCC 208821; H99	PSR00168	TP00701-19F	A04	8.07	-1.07	32.00	ug/mL
C0100587	10	P0761	FG_002	Cn H99	Cryptococcus neoformans	ATCC 208821; H99	PSR00168	TP00701-20F	A04	10.65	-1.67	32.00	ug/mL
C0100587	10	P0761	GP_020	Sa MRSa	Staphylococcus aureus	ATCC 43300; MRSA	PSR00168	TP00701-15V	A04	14.73	-0.18	32.00	ug/mL
C0100587	10	P0761	GP_020	Sa MRSa	Staphylococcus aureus	ATCC 43300; MRSA	PSR00168	TP00701-16V	A04	9.72	-0.38	32.00	ug/mL
C0100587	10	P0761	GN_001	Ec	Escherichia coli	ATCC 25922	PSR00168	TP00701-02C	A04	3.04	0.10	32.00	ug/mL
C0100587	10	P0761	GN_001	Ec	Escherichia coli	ATCC 25922	PSR00168	TP00701-01C	A04	1.25	-0.56	32.00	ug/mL
C0100587	10	P0761	GN_042	Pa	Pseudomonas aeruginosa	ATCC 27853	PSR00168	TP00701-08C	A04	15.62	-0.98	32.00	ug/mL
C0100587	10	P0761	GN_042	Pa	Pseudomonas aeruginosa	ATCC 27853	PSR00168	TP00701-07C	A04	-0.89	0.18	32.00	ug/mL
C0100587	10	P0761	GN_034	Ab	Acinetobacter baumannii	ATCC 19606	PSR00168	TP00701-06C	A04	8.62	0.11	32.00	ug/mL
C0100587	10	P0761	GN_034	Ab	Acinetobacter baumannii	ATCC 19606	PSR00168	TP00701-05C	A04	6.01	1.10	32.00	ug/mL
C0100587	10	P0761	GN_003	Kp MDR	Klebsiella pneumoniae	ATCC 700603; MDR	PSR00168	TP00701-03C	A04	-5.52	1.55	32.00	ug/mL
C0100587	10	P0761	GN_003	Kp MDR	Klebsiella pneumoniae	ATCC 700603; MDR	PSR00168	TP00701-04C	A04	0.46	0.62	32.00	ug/mL

Table S6: Methodological Details for Tables S3-5

Abbreviation	Code	Name	Description	Strain	Organsim	Type
Sa	GP_020	*Staphylococcus aureus*	MRSA	ATCC 43300	Bacteria	G+ve
Ec	GN_001	*Escherichia coli*	FDA control	ATCC 25922	Bacteria	G-ve
Kp	GN_003	*Klebsiella pneumoniae*	MDR	ATCC 700603	Bacteria	G-ve
Ab	GN_034	*Acinetobacter baumannii*	Type strain	ATCC 19606	Bacteria	G-ve
Pa	GN_042	*Pseudomonas aeruginosa*	Type strain	ATCC 27853	Bacteria	G-ve
Ca	FG_001	*Candida albicans*	CLSI reference	ATCC 90028	Fungi	Yeast
Cn	FG_002	*Cryptococcus neoformans var. grubii*	Type strain	H99; ATCC 208821	Fungi	Yeast

Antibacterial data collection

Inhibition of bacterial growth was determined measuring absorbance at 600 nm (OD_{600}), using a Tecan M1000 Pro monochromator plate reader. The percentage of growth inhibition was calculated for each well, using the negative control (media only) and positive control (bacteria without inhibitors) on the same plate as references.

Antifungal data collection

Growth inhibition of *C. albicans* was determined measuring absorbance at 530 nm (OD_{530}), while the growth inhibition of *C. neoformans* was determined measuring the difference in absorbance between 600 and 570 nm ($OD_{600-570}$), after the addition of resazurin (0.001% final concentration) and incubation at 35 °C for additional 2 h. The absorbance was measured using a Biotek Synergy HTX plate reader. The percentage of growth inhibition was calculated for each well, using the negative control (media only) and positive control (bacteria without inhibitors) on the same plate as references.

Inhibition

Percentage growth inhibition of an individual sample is calculated based on Negative controls (media only) and Positive Controls (bacterial/fungal media without inhibitors). Please note negative inhibition values indicate that the growth rate (or OD600) is higher compared to the Negative Control (Bacteria/fungi only, set to 0% inhibition). The growth rates for all bacteria and fungi has a variation of -/+ 10%, which is within the reported normal distribution of bacterial/fungal growth. Any significant variation (or outliers/hits) is identified by the modified Z-Score, and actives are selected by a combination of inhibition value and Z-Score.

Z-Score

Z-Score analysis is done to investigate outliers or hits among the samples. The Z-Score is calculated based on the sample population using a modified Z-Score method which accounts for possible skewed sample population. The modified method uses median and MAD (median average derviation) instead of average and sd, and a scaling factor [Iglewicz, B. & Hoaglin, D. C. Volume 16: How to Detect and Handle Outliers. The ASQC Basic Reference in Quality Control: Statistical Techniques, 1993]: M(i) = 0.6745 * (x(i) - median(x))/MAD). M(i) values of > |2.5| (absolute) label outliers or hits.

Quality Control

All screening is performed as two replica (n=2), with both replicas on different assay plates, but from single plating and performed in a single screening experiment (microbial incubation). Each individual value is reported in the table (see ..1 and ..2). In addition, two values are used as quality controls for individual plates: Z'-Factor [1 - (3 * (sd(NegCtrl)+sd(PosCtrl))/(average(PosCtrl)-average(NegCtrl)))] and Standard Antibiotic controls at different concentrations (>MIC and < MIC). The plate passes the quality control if Z'-Factor >0.4 and Standards are active and inactive at highest and lowest concentrations, respectively. Data not supplied.

Selection of Actives

A - [Active] Samples with inhibition values equal to or above 80% and abs(Z-Score) above |2.5| for either replicate (n=2 on different plates) were classed as active.
P - [Partial Active] compounds with inhibition values between 50.9% - 79.9% or abs(Z-Sore) below |2.5|.
I - Inactive compounds with inhibition values below 50% and/or abs(Z-Sore) below |2.5|.

Act_XX

Act_XX: Indicates if a compound is active in any of the assays against a specific organism (Sa, Ec, Kp, Pa, Kp, Ca or Cn), or organism classses (GN: Gram-negative, GP: Gram-positive). Please note that the flag indicates single activities even if the average Inhibition values suggests otherwise, in which case a manual adjustment of the flag

Act

Act: Indicates the number of organism-classes (GN,GP and FG) the compound has been found active against, 0 = no activity.

Sel

Sel: Indicates compounds that have been selected for further dose response studies, Hit-Confirmation. The selection includes all active as well as compounds with ambiguous results requiring confirmation of activity or inactivity.

Full Antimicrobial Screening Results: Tables S7-12

Table S7: Summary for Fosmidomycin and Compounds **4**, **5**, **6**, **7**, and **9**

CompoundID	CompoundName	ProjectID	RunID	Hit	Tox	Sa	Ec	Kp	Pa	Ab	Ca	Cn	Hk	Hm	Unit
C0107203	Fosmidomycin	P0822	HCR00168	0	0	>20	>20	>20	>20	>20	>20	>20	>20	>20	uM
C0107192	4	P0822	HCR00168	0	0	>32	>32	>32	>32	>32	>32	>32	>32	>32	ug/mL
C0107194	5	P0822	HCR00168	1	0	<=0.25	>32	>32	>32	>32	>32	>32	>32	>32	ug/mL
C0107193	6	P0822	HCR00168	0	0	>32	>32	>32	>32	>32	>32	>32	>32	>32	ug/mL
C0107195	7	P0822	HCR00168	0	0	>32	>32	>32	>32	>32	>32	>32	>32	>32	ug/mL
C0107191	9	P0822	HCR00168	1	0	>32	>32	>32	>32	>32	8	>32	>32	>32	ug/mL

Table S8: Details for Fosmidomycin and Compounds **4**, **5**, **6**, **7**, and **9**

CompoundID	CompoundName	ProjectID	RunID	Hit	Hit_GP	Hit_GN	Hit_FG	Tox
C0107203	Fosmidomycin	P0822	HCR00168	0	0	0	0	0
C0107192	4	P0822	HCR00168	0	0	0	0	0
C0107194	5	P0822	HCR00168	1	1	0	0	0
C0107193	6	P0822	HCR00168	0	0	0	0	0
C0107195	7	P0822	HCR00168	0	0	0	0	0
C0107191	9	P0822	HCR00168	1	0	0	1	0

CompoundID	CompoundName	ProjectID	RunID	Hit_GP	Sa_MIC	Sa_DMax	Sa_Act
C0107203	Fosmidomycin	P0822	HCR00168	0	>20.0; >20.0	10.6; 8.2; PCT;	I; I
C0107192	4	P0822	HCR00168	0	>32.0; >32.0	62.4; 73.7; PCT;	P; P
C0107194	5	P0822	HCR00168	1	<=0.250; >32.0	79.5; 84.4; PCT;	A; P
C0107193	6	P0822	HCR00168	0	>32.0; >32.0	63.8; 78.1; PCT;	P; P
C0107195	7	P0822	HCR00168	0	>32.0; >32.0	52.6; 63.0; PCT;	P; P
C0107191	9	P0822	HCR00168	0	>32.0; >32.0	-5.9; 6.3; PCT;	I; I

CompoundID	CompoundName	ProjectID	RunID	Hit_GN	Ec_MIC	Ec_DMax	Ec_Act	Kp_MIC	Kp_DMax	Kp_Act	Pa_MIC	Pa_DMax	Pa_Act	Ab_MIC	Ab_DMax	Ab_Act
C0107203	Fosmidomycin	P0822	HCR00168	0	>20.0; >20.0	64.1; 68.8; PCT;	P; P	>20.0; >20.0	35.7; 41.1; PCT;	I; P	>20.0; >20.0	23.9; 28.5; PCT;	I; I	>20.0; >20.0	4.1; 7.6; PCT;	I; I
C0107192	4	P0822	HCR00168	0	>32.0; >32.0	26.3; 30.7; PCT;	I; I	>32.0; >32.0	15.4; 26.0; PCT;	I; I	>32.0; >32.0	-1.3; 18.0; PCT;	I; I	>32.0; >32.0	60.9; 65.1; PCT;	P; P
C0107194	5	P0822	HCR00168	0	>32.0; >32.0	63.3; 66.2; PCT;	P; P	>32.0; >32.0	36.1; 36.3; PCT;	I; I	>32.0; >32.0	-2.5; 23.8; PCT;	I; I	>32.0; >32.0	19.5; 31.7; PCT;	I; I
C0107193	6	P0822	HCR00168	0	>32.0; >32.0	46.8; 49.5; PCT;	P; P	>32.0; >32.0	31.7; 36.7; PCT;	I; I	>32.0; >32.0	14.8; 18.6; PCT;	I; I	>32.0; >32.0	74.5; 78.3; PCT;	P; P
C0107195	7	P0822	HCR00168	0	>32.0; >32.0	-2.0; -8.0; PCT;	I; I	>32.0; >32.0	12.6; 14.1; PCT;	I; I	>32.0; >32.0	23.9; 8.2; PCT;	I; I	>32.0; >32.0	1.4; 7.7; PCT;	I; I
C0107191	9	P0822	HCR00168	0	>32.0; >32.0	-1.6; 0.6; PCT;	I; I	>32.0; >32.0	11.8; 16.5; PCT;	I; I	>32.0; >32.0	-1.2; -4.5; PCT;	I; I	>32.0; >32.0	11.1; 4.8; PCT;	I; I

CompoundID	CompoundName	ProjectID	RunID	Hit_FG	Ca_MIC	Ca_DMax	Ca_Act	Cn_MIC	Cn_DMax	Cn_Act
C0107203	Fosmidomycin	P0822	HCR00168	0	>20.0; >20.0	3.5; 7.8; PCT;	I; I	>20.0; >20.0	-4.6; 0.2; PCT;	I; I
C0107192	4	P0822	HCR00168	0	>32.0; >32.0	13.3; 8.4; PCT;	I; I	>32.0; >32.0	-6.3; -8.4; PCT;	I; I
C0107194	5	P0822	HCR00168	0	>32.0; >32.0	12.7; 17.7; PCT;	I; I	>32.0; >32.0	-0.4; -5.5; PCT;	I; I
C0107193	6	P0822	HCR00168	0	>32.0; >32.0	12.8; 13.9; PCT;	I; I	>32.0; >32.0	-11.0; -11.9; PCT;	I; I
C0107195	7	P0822	HCR00168	0	>32.0; >32.0	11.7; 18.5; PCT;	I; I	>32.0; >32.0	-15.0; 2.3; PCT;	I; I
C0107191	9	P0822	HCR00168	1	16.0; 8.00	95.0; 96.3; PCT;	A; A	>32.0; >32.0	-1.7; 4.0; PCT;	I; I

CompoundID	CompoundName	ProjectID	RunID	Tox	Hk_CC_{50}	Hk_DMax	Hk_Act	Hm_HC_{10}	Hm_DMax	Hm_Act
C0107203	Fosmidomycin	P0822	HCR00168	0	>20.0; >20.0	-2.0; 10.2; PCT;	I; I	>20.0; >20.0	1.1; 3.1; PCT;	I; I
C0107192	4	P0822	HCR00168	0	>32.0; >32.0	-1.2; -17.5; PCT;	I; I	>32.0; >32.0	5.5; 6.3; PCT;	I; I
C0107194	5	P0822	HCR00168	0	>32.0; >32.0	-7.4; 2.2; PCT;	I; I	>32.0; >32.0	2.6; 3.5; PCT;	I; I
C0107193	6	P0822	HCR00168	0	>32.0; >32.0	-3.2; -7.5; PCT;	I; I	>32.0; >32.0	-1.5; 0.1; PCT;	I; I
C0107195	7	P0822	HCR00168	0	>32.0; >32.0	-1.3; -14.5; PCT;	I; I	>32.0; >32.0	4.3; 5.0; PCT;	I; I
C0107191	9	P0822	HCR00168	0	>32.0; >32.0	0.6; 11.5; PCT;	I; I	>32.0; >32.0	6.7; 6.7; PCT;	I; I

Table S9: MIC Data for Fosmidomycin and Compounds **4**, **5**, **6**, **7**, and **9**

CompoundID	CompoundName	ProjectID	OrgID	OrgCode	Organism	Strain	PSRunID	TestPlateID	TestWellID	MIC	MIC_Unit	DMax
C0107203	Fosmidomycin	P0822	FG_002	Cn H99	Cryptococcus neoformans	ATCC 208821; H99	HCR00168	HC168-08-15	A20	>20.0	uM	-4.60
C0107203	Fosmidomycin	P0822	GN_034	Ab	Acinetobacter baumannii	ATCC 19606	HCR00168	HC168-08-05	A20	>20.0	uM	7.60
C0107203	Fosmidomycin	P0822	FG_002	Cn H99	Cryptococcus neoformans	ATCC 208821; H99	HCR00168	HC168-08-16	A20	>20.0	uM	0.20
C0107203	Fosmidomycin	P0822	GP_020	Sa MRSa	Staphylococcus aureus	ATCC 43300; MRSA	HCR00168	HC168-08-11	A20	>20.0	uM	10.60
C0107203	Fosmidomycin	P0822	GN_034	Ab	Acinetobacter baumannii	ATCC 19606	HCR00168	HC168-08-06	A20	>20.0	uM	4.10
C0107203	Fosmidomycin	P0822	GN_003	Kp MDR	Klebsiella pneumoniae	ATCC 700603; MDR	HCR00168	HC168-08-04	A20	>20.0	uM	41.10
C0107203	Fosmidomycin	P0822	GN_042	Pa	Pseudomonas aeruginosa	ATCC 27853	HCR00168	HC168-08-21	A20	>20.0	uM	28.50
C0107203	Fosmidomycin	P0822	GN_001	Ec	Escherichia coli	ATCC 25922	HCR00168	HC168-08-01	A20	>20.0	uM	64.10
C0107203	Fosmidomycin	P0822	GN_042	Pa	Pseudomonas aeruginosa	ATCC 27853	HCR00168	HC168-08-22	A20	>20.0	uM	23.90
C0107203	Fosmidomycin	P0822	FG_001	Ca	Candida albicans	ATCC 90028	HCR00168	HC168-08-13	A20	>20.0	uM	7.80
C0107203	Fosmidomycin	P0822	GN_003	Kp MDR	Klebsiella pneumoniae	ATCC 700603; MDR	HCR00168	HC168-08-03	A20	>20.0	uM	35.70
C0107203	Fosmidomycin	P0822	FG_001	Ca	Candida albicans	ATCC 90028	HCR00168	HC168-08-14	A20	>20.0	uM	3.50
C0107203	Fosmidomycin	P0822	GN_001	Ec	Escherichia coli	ATCC 25922	HCR00168	HC168-08-02	A20	>20.0	uM	68.80
C0107203	Fosmidomycin	P0822	GP_020	Sa MRSa	Staphylococcus aureus	ATCC 43300; MRSA	HCR00168	HC168-08-12	A20	>20.0	uM	8.20
C0107192	4	P0822	GN_003	Kp MDR	Klebsiella pneumoniae	ATCC 700603; MDR	HCR00168	HC168-09-03	A03	>32.0	ug/mL	26.00
C0107192	4	P0822	GN_003	Kp MDR	Klebsiella pneumoniae	ATCC 700603; MDR	HCR00168	HC168-09-04	A03	>32.0	ug/mL	15.40
C0107192	4	P0822	GN_034	Ab	Acinetobacter baumannii	ATCC 19606	HCR00168	HC168-09-06	A03	>32.0	ug/mL	65.10
C0107192	4	P0822	GN_034	Ab	Acinetobacter baumannii	ATCC 19606	HCR00168	HC168-09-05	A03	>32.0	ug/mL	60.90
C0107192	4	P0822	FG_002	Cn H99	Cryptococcus neoformans	ATCC 208821; H99	HCR00168	HC168-09-15	A03	>32.0	ug/mL	-6.30
C0107192	4	P0822	FG_002	Cn H99	Cryptococcus neoformans	ATCC 208821; H99	HCR00168	HC168-09-16	A03	>32.0	ug/mL	-8.40
C0107192	4	P0822	GN_001	Ec	Escherichia coli	ATCC 25922	HCR00168	HC168-09-02	A03	>32.0	ug/mL	26.30
C0107192	4	P0822	GN_042	Pa	Pseudomonas aeruginosa	ATCC 27853	HCR00168	HC168-09-22	A03	>32.0	ug/mL	-1.30
C0107192	4	P0822	GN_042	Pa	Pseudomonas aeruginosa	ATCC 27853	HCR00168	HC168-09-21	A03	>32.0	ug/mL	18.00
C0107192	4	P0822	GP_020	Sa MRSa	Staphylococcus aureus	ATCC 43300; MRSA	HCR00168	HC168-09-12	A03	>32.0	ug/mL	73.70
C0107192	4	P0822	FG_001	Ca	Candida albicans	ATCC 90028	HCR00168	HC168-09-13	A03	>32.0	ug/mL	8.40
C0107192	4	P0822	GN_001	Ec	Escherichia coli	ATCC 25922	HCR00168	HC168-09-01	A03	>32.0	ug/mL	30.70
C0107192	4	P0822	FG_001	Ca	Candida albicans	ATCC 90028	HCR00168	HC168-09-14	A03	>32.0	ug/mL	13.30
C0107192	4	P0822	GP_020	Sa MRSa	Staphylococcus aureus	ATCC 43300; MRSA	HCR00168	HC168-09-11	A03	>32.0	ug/mL	62.40
C0107194	5	P0822	GN_003	Kp MDR	Klebsiella pneumoniae	ATCC 700603; MDR	HCR00168	HC168-09-03	A07	>32.0	ug/mL	36.10
C0107194	5	P0822	GN_003	Kp MDR	Klebsiella pneumoniae	ATCC 700603; MDR	HCR00168	HC168-09-04	A07	>32.0	ug/mL	36.30
C0107194	5	P0822	GN_034	Ab	Acinetobacter baumannii	ATCC 19606	HCR00168	HC168-09-06	A07	>32.0	ug/mL	31.70
C0107194	5	P0822	GN_034	Ab	Acinetobacter baumannii	ATCC 19606	HCR00168	HC168-09-05	A07	>32.0	ug/mL	19.50
C0107194	5	P0822	FG_002	Cn H99	Cryptococcus neoformans	ATCC 208821; H99	HCR00168	HC168-09-15	A07	>32.0	ug/mL	-0.40
C0107194	5	P0822	FG_002	Cn H99	Cryptococcus neoformans	ATCC 208821; H99	HCR00168	HC168-09-16	A07	>32.0	ug/mL	-5.50
C0107194	5	P0822	GN_001	Ec	Escherichia coli	ATCC 25922	HCR00168	HC168-09-02	A07	>32.0	ug/mL	63.30
C0107194	5	P0822	GN_042	Pa	Pseudomonas aeruginosa	ATCC 27853	HCR00168	HC168-09-21	A07	>32.0	ug/mL	23.80
C0107194	5	P0822	GN_042	Pa	Pseudomonas aeruginosa	ATCC 27853	HCR00168	HC168-09-22	A07	>32.0	ug/mL	-2.50
C0107194	5	P0822	GP_020	Sa MRSa	Staphylococcus aureus	ATCC 43300; MRSA	HCR00168	HC168-09-12	A07	<=0.250	ug/mL	84.40
C0107194	5	P0822	FG_001	Ca	Candida albicans	ATCC 90028	HCR00168	HC168-09-13	A07	>32.0	ug/mL	12.70
C0107194	5	P0822	GN_001	Ec	Escherichia coli	ATCC 25922	HCR00168	HC168-09-01	A07	>32.0	ug/mL	66.20
C0107194	5	P0822	FG_001	Ca	Candida albicans	ATCC 90028	HCR00168	HC168-09-14	A07	>32.0	ug/mL	17.70
C0107194	5	P0822	GP_020	Sa MRSa	Staphylococcus aureus	ATCC 43300; MRSA	HCR00168	HC168-09-11	A07	>32.0	ug/mL	79.50
C0107193	6	P0822	GN_003	Kp MDR	Klebsiella pneumoniae	ATCC 700603; MDR	HCR00168	HC168-09-03	A05	>32.0	ug/mL	36.70
C0107193	6	P0822	GN_003	Kp MDR	Klebsiella pneumoniae	ATCC 700603; MDR	HCR00168	HC168-09-04	A05	>32.0	ug/mL	31.70
C0107193	6	P0822	GN_034	Ab	Acinetobacter baumannii	ATCC 19606	HCR00168	HC168-09-06	A05	>32.0	ug/mL	78.30
C0107193	6	P0822	GN_034	Ab	Acinetobacter baumannii	ATCC 19606	HCR00168	HC168-09-05	A05	>32.0	ug/mL	74.50
C0107193	6	P0822	FG_002	Cn H99	Cryptococcus neoformans	ATCC 208821; H99	HCR00168	HC168-09-15	A05	>32.0	ug/mL	-11.00
C0107193	6	P0822	FG_002	Cn H99	Cryptococcus neoformans	ATCC 208821; H99	HCR00168	HC168-09-16	A05	>32.0	ug/mL	-11.90
C0107193	6	P0822	GN_001	Ec	Escherichia coli	ATCC 25922	HCR00168	HC168-09-02	A05	>32.0	ug/mL	46.80
C0107193	6	P0822	GN_042	Pa	Pseudomonas aeruginosa	ATCC 27853	HCR00168	HC168-09-21	A05	>32.0	ug/mL	18.60
C0107193	6	P0822	GN_042	Pa	Pseudomonas aeruginosa	ATCC 27853	HCR00168	HC168-09-22	A05	>32.0	ug/mL	14.80
C0107193	6	P0822	GP_020	Sa MRSa	Staphylococcus aureus	ATCC 43300; MRSA	HCR00168	HC168-09-12	A05	>32.0	ug/mL	78.10
C0107193	6	P0822	FG_001	Ca	Candida albicans	ATCC 90028	HCR00168	HC168-09-13	A05	>32.0	ug/mL	12.80
C0107193	6	P0822	GN_001	Ec	Escherichia coli	ATCC 25922	HCR00168	HC168-09-01	A05	>32.0	ug/mL	49.50
C0107193	6	P0822	FG_001	Ca	Candida albicans	ATCC 90028	HCR00168	HC168-09-14	A05	>32.0	ug/mL	13.90
C0107193	6	P0822	GP_020	Sa MRSa	Staphylococcus aureus	ATCC 43300; MRSA	HCR00168	HC168-09-11	A05	>32.0	ug/mL	63.80
C0107195	7	P0822	GN_003	Kp MDR	Klebsiella pneumoniae	ATCC 700603; MDR	HCR00168	HC168-09-03	A09	>32.0	ug/mL	14.10
C0107195	7	P0822	GN_003	Kp MDR	Klebsiella pneumoniae	ATCC 700603; MDR	HCR00168	HC168-09-04	A09	>32.0	ug/mL	12.60
C0107195	7	P0822	GN_034	Ab	Acinetobacter baumannii	ATCC 19606	HCR00168	HC168-09-06	A09	>32.0	ug/mL	7.70
C0107195	7	P0822	GN_034	Ab	Acinetobacter baumannii	ATCC 19606	HCR00168	HC168-09-05	A09	>32.0	ug/mL	1.40
C0107195	7	P0822	FG_002	Cn H99	Cryptococcus neoformans	ATCC 208821; H99	HCR00168	HC168-09-15	A09	>32.0	ug/mL	2.30
C0107195	7	P0822	FG_002	Cn H99	Cryptococcus neoformans	ATCC 208821; H99	HCR00168	HC168-09-16	A09	>32.0	ug/mL	-15.00
C0107195	7	P0822	GN_001	Ec	Escherichia coli	ATCC 25922	HCR00168	HC168-09-02	A09	>32.0	ug/mL	-2.00
C0107195	7	P0822	GN_042	Pa	Pseudomonas aeruginosa	ATCC 27853	HCR00168	HC168-09-21	A09	>32.0	ug/mL	23.90
C0107195	7	P0822	GN_042	Pa	Pseudomonas aeruginosa	ATCC 27853	HCR00168	HC168-09-22	A09	>32.0	ug/mL	8.20
C0107195	7	P0822	GP_020	Sa MRSa	Staphylococcus aureus	ATCC 43300; MRSA	HCR00168	HC168-09-12	A09	>32.0	ug/mL	63.00
C0107195	7	P0822	FG_001	Ca	Candida albicans	ATCC 90028	HCR00168	HC168-09-13	A09	>32.0	ug/mL	18.50
C0107195	7	P0822	GN_001	Ec	Escherichia coli	ATCC 25922	HCR00168	HC168-09-01	A09	>32.0	ug/mL	-8.00
C0107195	7	P0822	FG_001	Ca	Candida albicans	ATCC 90028	HCR00168	HC168-09-14	A09	>32.0	ug/mL	11.70
C0107195	7	P0822	GP_020	Sa MRSa	Staphylococcus aureus	ATCC 43300; MRSA	HCR00168	HC168-09-11	A09	>32.0	ug/mL	52.60
C0107191	9	P0822	GN_003	Kp MDR	Klebsiella pneumoniae	ATCC 700603; MDR	HCR00168	HC168-09-03	A01	>32.0	ug/mL	11.80
C0107191	9	P0822	GN_003	Kp MDR	Klebsiella pneumoniae	ATCC 700603; MDR	HCR00168	HC168-09-04	A01	>32.0	ug/mL	16.50
C0107191	9	P0822	GN_034	Ab	Acinetobacter baumannii	ATCC 19606	HCR00168	HC168-09-06	A01	>32.0	ug/mL	4.80
C0107191	9	P0822	GN_034	Ab	Acinetobacter baumannii	ATCC 19606	HCR00168	HC168-09-05	A01	>32.0	ug/mL	11.10
C0107191	9	P0822	FG_002	Cn H99	Cryptococcus neoformans	ATCC 208821; H99	HCR00168	HC168-09-15	A01	>32.0	ug/mL	4.00
C0107191	9	P0822	FG_002	Cn H99	Cryptococcus neoformans	ATCC 208821; H99	HCR00168	HC168-09-16	A01	>32.0	ug/mL	-1.70
C0107191	9	P0822	GN_001	Ec	Escherichia coli	ATCC 25922	HCR00168	HC168-09-02	A01	>32.0	ug/mL	-1.60
C0107191	9	P0822	GN_042	Pa	Pseudomonas aeruginosa	ATCC 27853	HCR00168	HC168-09-22	A01	>32.0	ug/mL	-4.50
C0107191	9	P0822	GN_042	Pa	Pseudomonas aeruginosa	ATCC 27853	HCR00168	HC168-09-21	A01	>32.0	ug/mL	-1.20
C0107191	9	P0822	GP_020	Sa MRSa	Staphylococcus aureus	ATCC 43300; MRSA	HCR00168	HC168-09-12	A01	>32.0	ug/mL	6.30
C0107191	9	P0822	FG_001	Ca	Candida albicans	ATCC 90028	HCR00168	HC168-09-13	A01	16	ug/mL	95.00
C0107191	9	P0822	GN_001	Ec	Escherichia coli	ATCC 25922	HCR00168	HC168-09-01	A01	>32.0	ug/mL	0.60
C0107191	9	P0822	FG_001	Ca	Candida albicans	ATCC 90028	HCR00168	HC168-09-14	A01	8	ug/mL	96.30
C0107191	9	P0822	GP_020	Sa MRSa	Staphylococcus aureus	ATCC 43300; MRSA	HCR00168	HC168-09-11	A01	>32.0	ug/mL	-5.90

Table S10: Cytotoxicity Data for Fosmidomycin and Compounds **4**, **5**, **6**, **7**, and **9**

CompoundID	CompoundName	ProjectID	OrgID	OrgCode	Organism	Strain	PSRunID	TestPlateID	TestWellID	CC50	CC50_Unit	DMax
C0107203	Fosmidomycin	P0822	MA_007	Hek	Homo sapiens	HEK293; ATCC CRL1573	HCR00168	HC168-08-17	A20	>20.0	uM	10.20
C0107203	Fosmidomycin	P0822	MA_007	Hek	Homo sapiens	HEK293; ATCC CRL1573	HCR00168	HC168-08-18	A20	>20.0	uM	-2.00
C0107192	4	P0822	MA_007	Hek	Homo sapiens	HEK293; ATCC CRL1573	HCR00168	HC168-09-17	A03	>32.0	ug/mL	-1.20
C0107192	4	P0822	MA_007	Hek	Homo sapiens	HEK293; ATCC CRL1573	HCR00168	HC168-09-18	A03	>32.0	ug/mL	-17.50
C0107194	5	P0822	MA_007	Hek	Homo sapiens	HEK293; ATCC CRL1573	HCR00168	HC168-09-17	A07	>32.0	ug/mL	2.20
C0107194	5	P0822	MA_007	Hek	Homo sapiens	HEK293; ATCC CRL1573	HCR00168	HC168-09-18	A07	>32.0	ug/mL	-7.40
C0107193	6	P0822	MA_007	Hek	Homo sapiens	HEK293; ATCC CRL1573	HCR00168	HC168-09-17	A05	>32.0	ug/mL	-3.20
C0107193	6	P0822	MA_007	Hek	Homo sapiens	HEK293; ATCC CRL1573	HCR00168	HC168-09-18	A05	>32.0	ug/mL	-7.50
C0107195	7	P0822	MA_007	Hek	Homo sapiens	HEK293; ATCC CRL1573	HCR00168	HC168-09-17	A09	>32.0	ug/mL	-1.30
C0107195	7	P0822	MA_007	Hek	Homo sapiens	HEK293; ATCC CRL1573	HCR00168	HC168-09-18	A09	>32.0	ug/mL	-14.50
C0107191	9	P0822	MA_007	Hek	Homo sapiens	HEK293; ATCC CRL1573	HCR00168	HC168-09-17	A01	>32.0	ug/mL	0.60
C0107191	9	P0822	MA_007	Hek	Homo sapiens	HEK293; ATCC CRL1573	HCR00168	HC168-09-18	A01	>32.0	ug/mL	11.50

Table S11: Haemolysis Data for Fosmidomycin and Compounds **4**, **5**, **6**, **7**, and **9**

CompoundID	CompoundName	ProjectID	OrgID	OrgCode	Organism	Strain	PSRunID	TestPlateID	TestWellID	HC10	HC50	Unit	DMax
C0107203	Fosmidomycin	P0822	HA_150	RBC	Homo sapiens	Red blood cell	HCR00168	HC168-08-19	A20	>20.0	>20.0	uM	1.10
C0107203	Fosmidomycin	P0822	HA_150	RBC	Homo sapiens	Red blood cell	HCR00168	HC168-08-20	A20	>20.0	>20.0	uM	3.10
C0107192	4	P0822	HA_150	RBC	Homo sapiens	Red blood cell	HCR00168	HC168-09-19	A03	>32.0	>32.0	ug/mL	5.50
C0107192	4	P0822	HA_150	RBC	Homo sapiens	Red blood cell	HCR00168	HC168-09-20	A03	>32.0	>32.0	ug/mL	6.30
C0107194	5	P0822	HA_150	RBC	Homo sapiens	Red blood cell	HCR00168	HC168-09-19	A07	>32.0	>32.0	ug/mL	3.50
C0107194	5	P0822	HA_150	RBC	Homo sapiens	Red blood cell	HCR00168	HC168-09-20	A07	>32.0	>32.0	ug/mL	2.60
C0107193	6	P0822	HA_150	RBC	Homo sapiens	Red blood cell	HCR00168	HC168-09-19	A05	>32.0	>32.0	ug/mL	0.10
C0107193	6	P0822	HA_150	RBC	Homo sapiens	Red blood cell	HCR00168	HC168-09-20	A05	>32.0	>32.0	ug/mL	-1.50
C0107195	7	P0822	HA_150	RBC	Homo sapiens	Red blood cell	HCR00168	HC168-09-19	A09	>32.0	>32.0	ug/mL	4.30
C0107195	7	P0822	HA_150	RBC	Homo sapiens	Red blood cell	HCR00168	HC168-09-20	A09	>32.0	>32.0	ug/mL	5.00
C0107191	9	P0822	HA_150	RBC	Homo sapiens	Red blood cell	HCR00168	HC168-09-19	A01	>32.0	>32.0	ug/mL	6.70
C0107191	9	P0822	HA_150	RBC	Homo sapiens	Red blood cell	HCR00168	HC168-09-20	A01	>32.0	>32.0	ug/mL	6.70

Table S12: Methodological Details for Tables S7-11

Abbreviation	Code	Name	Description	Strain	Organsim	Type	Media	PlateType
**Sa**	GP_020	*Staphylococcus aureus*	MRSA	ATCC 43300	Bacteria	G+ve	CAMHB	NBS
**Ec**	GN_001	*Escherichia coli*	FDA control	ATCC 25922	Bacteria	G-ve	CAMHB	NBS
**Kp**	GN_003	*Klebsiella pneumoniae*	MDR	ATCC 700603	Bacteria	G-ve	CAMHB	NBS
**Ab**	GN_034	*Acinetobacter baumannii*	Type strain	ATCC 19606	Bacteria	G-ve	CAMHB	NBS
**Pa**	GN_042	*Pseudomonas aeruginosa*	Type strain	ATCC 27853	Bacteria	G-ve	CAMHB	NBS
**Ca**	FG_001	*Candida albicans*	CLSI reference	ATCC 90028	Fungi	Yeast	YNB	NBS
**Cn**	FG_002	*Cryptococcus neoformans var. grubii*	Type strain	H99; ATCC 208821	Fungi	Yeast	YNB	NBS
**Hk**	MA_007	Human embryonic kidney cells	HEK-293	ATCC CRL-1573	Human	Eukaryotes	DMEM 10% FBS	TC-PS
**Hm**	HA_150	Human red blood cells	RBC		Human	Eukaryotes		PS

Summary
Table with Percentile(50%) values of MIC, CC_{50} (cytotoxicity) and HC_{10} (haemolytic activity) for each organism

Detail
Table with individual MIC, CC50 (cytotoxicity) and HC10 (haemolytic activity) values for each organism, as well as Dmax values.

MIC, Cytotox, Haemolysis
Individual data points for import in in-house databases. Haemolysis also reports the HC50 values, which is used to calculate the HC10 values

Compound Concentration
Compounds were plated as a 2-fold dose response from 32 to 0.25 µg/mL (or 20 to 0.156 uM), with a maximum of 0.5% DMSO, final in assay concentration.

Antibacterial data collection
Growth inhibition of all bacteria was determined measuring absorbance at 600 nm (OD_{600}), using a Tecan M1000 Pro monochromator plate reader. The percentage of growth inhibition was calculated for each well, using the negative control (media only) and positive control (bacteria without inhibitors) on the same plate as references.

Antifungal data collection
Growth inhibition of *C. albicans* was determined measuring absorbance at 530 nm (OD_{530}), while the growth inhibition of *C. neoformans* was determined measuring the difference in absorbance between 600 and 570 nm ($OD_{600\text{-}570}$), after the addition of resazurin (0.001% final concentration) and incubation at 35 °C for additional 2 h. The absorbance was measured using a Biotek Synergy HTX plate reader. The percentage of growth inhibition was calculated for each well, using the negative control (media only) and positive control (bacteria without inhibitors) on the same plate as references.

Cytotoxicity data collection
Growth inhibition of HEK293 cells was determined measuring fluorescence at ex:530/10 nm and em:590/10 nm ($F_{560/590}$), after the addition of resazurin (25 ug/mL final concentration) and incubation at 37 °C and 5% CO2, for additional 3 h. The fluorescence was measured using a Tecan M1000 Pro monochromator plate reader. The percentage of growth inhibition was calculated for each well, using the Negative Control (media only) and Positive Control (cell culture without inhibitors) on the same plate as references.

Inhibition
Percentage growth inhibition of individual samples and concentration are calculated based on Negative Controls (media only; 100%) and Positive Controls (bacterial/cell media without inhibitors: 0%). Please note Negative inhibition values indicate that the growth rate is higher compared to the Negative Control (Bacteria/Cell only, set to 0% inhibition). The growth rates for all organims has a variation of -/+ 10%, which lies is within expected normal distribution.

MIC (Minimum Inhibitory Concentration)
The minimum inhibitory concentration (MIC) was determined following the CLSI guidelines, identifying the lowest concentration at which full inhibition of the bacteria or fungi has been detected. Full inhibition of growth has been defined at <= 20% growth (or >80% inhibition), and concentrations have only been selected if the next highest concentration displayed full inhibition (i.e. 80-100%) as well (eliminating 'singlet' active concentration). Please note MIC values are discrete values based on the concentration in a specific well. Any value with > indicates that sample displays no activity (low DMax value) or partial activity at the highest tested concentration (higher DMax value).

CC_{50} (Concentration at 50% Cytotoxicity)
CC_{50} (Concentration at 50% Cytotoxicity) were calculated by curve fitting the inhibition values vs. log(concentration) using Sigmoidal dose-response function, with variable values for bottom, top and slope. The curve fitting is implemented using Pipeline Pilot's dose-response component (giving similar results to similar tools such as GraphPad's Prism and IDBS's XlFit). Any value with > indicates a sample with no activity (low DMax value) or samples with CC_{50} values above the maximum tested concentration (higher DMax value)

HC_{10} (Concentration at 10% Haemolytic activity)
HC_{10} (Concentration at 10% Haemolytic activity) were calculated by curve fitting the inhibition values vs. log(concentration) using Sigmoidal dose-response function, with variable values for bottom, top and slope. The curve fitting is implemented using Pipeline Pilot's dose-response component (giving similar results to similar tools such as GraphPad's Prism and IDBS's XlFit). The curve fitting resulted in HC_{50} (50%) values, which are converted into HC10 by HC_{10}=HC_{50}*(10/90)^(1/Slope); Any value with > indicates a sample with no activity (low DMax value) or samples with HC_{10} values above the maximum tested concentration (higher DMax value)

DMax (Maximum Response)
DMax represents the highest pecentage inhibition response for all concentrations tested. The value helps to indicate if samples are displaying only partial response at the screened concentration, suggesting that the sample might be fully active at a higher concentration or if the sample only exhibits partial inhibition. In addition, the value indicates if samples have been active but curve fitting failed, mostly due to the fact that only the single highest concnetration was active.

Quality Control
All screening is performed as two replica (n=2), with both replicas on different assay plates, but from single plating and performed in a single screening experiment (microbial incubation). Each individual value is reported in the table (see ..1 and ..2). In addition, two values are used as quality controls for individual plates: Z'-Factor=[1 - (3*(sd(NegCtrl)+sd(PosCtrl))/(average(PosCtrl)-average(NegCtrl)))] and Standard Antibiotic controls at different concentrations (>MIC and < MIC). The plate passes the quality control if Z'-Factor >0.4 and Standards are active and inactive at highest and lowest concentrations, respectively. Data not supplied.

Selection of Hits
A - Sample with MIC <= 16 ug/mL or <= 10 uM are declared as a hit. For toxicity (cytotoxicity and haemolytic activity) all sample/s with CC_{50}/HC_{10} <= maximum tested concentration are considered active, or toxic. Since the maximum tested concentration is the same for toxicity and antimicrobial activity, no theraputic index (MIC/CC_{50} or MIC/HC_{10}) can be calculated and all sample/s with toxic activity are flaged.
I - Inactive samples with MIC/CC_{50}/HC_{10} > 16 ug/mL or > 10 uM.

Hit
Hit: Indicates the number of organism-classes (GN,GP and FG) the compound has been found active against, 0 = no activity.

Hit_XX
Hit_XX: Indicates if a compound is active in any of the assays against a specific organism (Sa, Ec, Kp, Pa, Kp, Ca or Cn), or organism classses (GN: Gram-negative, GP: Gram-positive). Please note that the flag indicates single activities even if the average or median MIC value suggests otherwise, in which case a manual adjustment of the flag might be appropiate.

Tox
Tox: Indicates samples with cytotoxicity (Dmax >50%) or haemolytic activity (Dmax >10%). This more stringent threshold is used as the samples were tested at the same concentration range as MIC, but Toxicity assays are usually run at higher sample concentration to evaluate their theraputic index (difference between MIC and CC50). This stringent threshold is used to flag any partial toxicity, which would show well defined toxicity (CC50 or HC10) at higher concentrations.
